# Zika virus produces two isoforms of the NS2A protein that play mutually exclusive roles in viral replication and virion assembly

**DOI:** 10.1371/journal.ppat.1014609

**Published:** 2026-10-12

**Authors:** Breanna N. Landry, Olivia J. Klann, Hannah C.H. Hauch, Cheyanne L. Bemis, Jonathan E. Owen, Jayden Harris, Trisha R. Barnard, Christopher J. Neufeldt, Selena M. Sagan

**Affiliations:** 1 Department of Microbiology & Immunology, University of British Columbia, Vancouver, BC, Canada; 2 Department of Microbiology & Immunology, McGill University, Montreal, QC, Canada; 3 Department of Microbiology and Immunology, School of Medicine, Emory University, Atlanta, Georgia, United States of America; 4 Department of Biochemistry, McGill University, Montreal, QC, Canada; Fundación Instituto Leloir-CONICET, ARGENTINA

## Abstract

Zika virus (ZIKV), dengue virus, and several other orthoflaviviruses cause disease outbreaks in human and animal populations worldwide. The orthoflaviviral nonstructural protein 2A (NS2A) is critical for both viral replication and virion assembly. However, due to its small size, hydrophobicity, and the absence of structural information or protein-specific antibodies, we lack a clear understanding of how NS2A participates in these disparate processes. Here, using endogenously tagged NS2A-containing viral RNAs, we reveal that two NS2A isoforms are present during ZIKV infection, including both full-length NS2A and a C-terminally truncated isoform, termed NS2Aα. Phylogenetic and conservation analyses combined with polyprotein cleavage assays suggest that both NS2A isoforms are generated via the viral protease and are conserved across the *Orthoflavivirus* genus. Finally, using assays for viral replication and infectious particle production, we demonstrate that the two distinct NS2A isoforms function at different stages of the viral life cycle, with full-length NS2A required for viral RNA replication and NS2Aα important for virion assembly. Taken together, our data helps shed light on the form(s) and function(s) of the NS2A protein and suggests that each NS2A isoform plays a mutually exclusive role in the orthoflaviviral life cycle.

## Author summary

The *Orthoflavivirus* NS2A protein is a small, hydrophobic, integral membrane protein implicated in both viral RNA replication and virion assembly. However, how NS2A participates in these two disparate processes remains unclear. In this study, we found that during ZIKV infection there are two NS2A isoforms generated, including full-length NS2A and a distinct, predominant isoform that is truncated in size, termed NS2Aα. We found that NS2Aα biogenesis was mediated by the viral NS3 protease at a unique internal cleavage motif, which is conserved across representative mosquito- and tick-borne orthoflaviviruses. Furthermore, we observed that the two NS2A isoforms play mutually exclusive roles in the viral life cycle with full-length NS2A critical for viral RNA replication and the morphology of viral replication organelles, while the NS2Aα isoform was critical for genome packaging and infectious particle production. These results provide important insights into the role of the NS2A protein and its isoforms in the ZIKV life cycle and reveal a high level of conservation across the *Orthoflavivirus* genus.

## Introduction

Zika virus (ZIKV) belongs to the *Orthoflavivirus* genus of the *Flaviviridae* family. Members of this genus are typically transmitted to vertebrates by mosquitoes or ticks and result in significant human morbidity and mortality, with more than 3 billion people at risk of infection annually [1–4]. Symptoms of orthoflavivirus infection can range from mild febrile illness to devastating outcomes, which may include neurological impairment and/or hemorrhagic fever [5–7]. Importantly, climate change threatens to expand the ecological niche of orthoflavivirus vectors, which is likely to increase the frequency of infection [1,8]. Unfortunately, even with the prospect of this growing disease burden, vaccines against many of these pathogens are lacking and there are currently no approved antivirals for orthoflavivirus infections [9,10]. The rational design of direct-acting antivirals will require a deeper mechanistic understanding of the different stages of the orthoflavivirus life cycle, including the processes of viral RNA replication and virion assembly [11–14].

The ZIKV genome is a positive-sense RNA approximately 11 kb in length that contains a 5´ cap but lacks a 3´ poly(A)-tail [15,16]. The viral RNA encodes a polyprotein that is processed co- and post-translationally by host and viral proteases to yield the 10 mature viral proteins. This includes 3 structural proteins [capsid (C), pre-membrane (prM) and envelope (E)], which form the virion, and 7 nonstructural (NS) proteins (NS1, NS2A, NS2B, NS3, NS4A, NS4B, and NS5), which facilitate viral RNA replication and virion assembly [3,11,13]. While several of the viral NS proteins have been characterized in detail, we still lack a comprehensive understanding of both the form and function of the NS2A protein [11,13,17].

Briefly, NS2A is a small (~22 kDa) hydrophobic protein that has been implicated in both viral RNA replication and virion assembly, although its precise role(s) in these processes remain unclear [13,18–23]. Nonetheless, previous studies in several orthoflaviviruses have indicated that NS2A colocalizes with double-stranded RNA in discrete cytoplasmic foci, which are presumably the sites of viral RNA replication [21,24,25]. Additionally, while NS2A has been implicated in membrane rearrangement, it is not yet clear whether this activity is required for viral replication organelle (vRO) biogenesis [26,27]. In addition to colocalizing with vROs, NS2A has also been shown to interact with several structural (prM and E) and NS proteins (NS2B and NS3) as well as the 3´ untranslated region (UTR) of the viral genomic RNA [20,21,28]. Furthermore, there have been reports from specific orthoflaviviruses that suggest that NS2A can also interact with both the NS4A (tick-borne encephalitis virus, TBEV) and NS5 proteins (Kunjin virus, KUNV) [24,29]. Through these activities, it is thought that NS2A may serve as a hub or scaffold for both viral RNA replication complex formation and virion assembly [20,21,24,30]. While NS2A has been shown to be required *in cis* for viral RNA replication, it can be supplied *in trans* for virion assembly [19,22]. Notably, in yellow fever virus (YFV), Japanese encephalitis virus (JEV), and tembusu virus (TMUV), a truncated isoform of NS2A has also been identified, termed NS2Aα, suggesting the existence of at least two distinct isoforms of NS2A during orthoflavivirus infection [31–34]. However, it remains unclear whether these two NS2A isoforms are conserved across the genus, and whether they play distinct roles in the viral life cycle.

Herein, we sought to further characterize the form(s) and function(s) of the ZIKV NS2A protein by creating endogenously tagged NS2A-containing viral RNAs. We found that, like YFV, JEV, and TMUV, ZIKV also produces an NS2Aα isoform, and we establish that it is the predominant form of the protein present during infection. Moreover, we mapped the NS2Aα cleavage motif and demonstrate that NS2Aα biogenesis is mediated by the viral NS3 protease. Through phylogenetic analyses and ectopic expression, we show that the NS2Aα cleavage motif is conserved and that the viral NS3 protease mediates its biogenesis across a broad range of mosquito- and tick-borne orthoflaviviruses. Finally, we demonstrate that while full-length NS2A is required for viral RNA replication and vRO biogenesis, NS2Aα is critical for genome packaging and infectious particle production. Collectively, our findings indicate that the NS2Aα isoform is conserved across the *Orthoflavivirus* genus, and that the two NS2A isoforms have mutually exclusive roles in the viral life cycle.

## Results

### The NS2Aα isoform is the predominant form of NS2A during ZIKV infection in cell culture

As NS2A has been implicated in both viral RNA replication and virion assembly, we were interested in understanding the features of this protein that allow it to participate in these disparate processes [18–22]. To this end, we inserted a hemagglutinin (HA)-tag onto the N-terminus of the NS2A protein in the context of the ZIKV genome (Fig 1A). As previously shown, HA-NS2A ZIKV has a similar plaque phenotype and generates viral titers comparable to wild-type (WT) ZIKV (S1A Fig) [21]. Furthermore, introduction of the HA-NS2A into a subgenomic replicon also revealed similar replication kinetics to WT (S1B Fig). Interestingly, when we performed an infection time course in Vero cells and probed for NS2A using the HA-tag, we observed two isoforms via Western blot, one that corresponds to full-length NS2A (~21 kDa) and a second smaller isoform, termed NS2Aα (~17 kDa) (Fig 1B). Moreover, NS2Aα was the predominant isoform of the protein generated during infection with a ratio of 6.00 ± 1.98:1 of NS2Aα:NS2A at 24 h post-infection (Fig 1B). Additionally, the NS2Aα isoform was also observed to be the predominant NS2A isoform during infection time courses in both human (A549) and mosquito (C6/36) cells (S2 Fig). These findings are consistent with previous studies in select orthoflaviviruses, including YFV, JEV and TMUV, which also observed the NS2Aα isoform [31,33,35]. Taken together, our results suggest that the ZIKV NS2Aα isoform is the predominant form of the protein during infection in cultured mammalian and mosquito cells.

**Fig 1:**
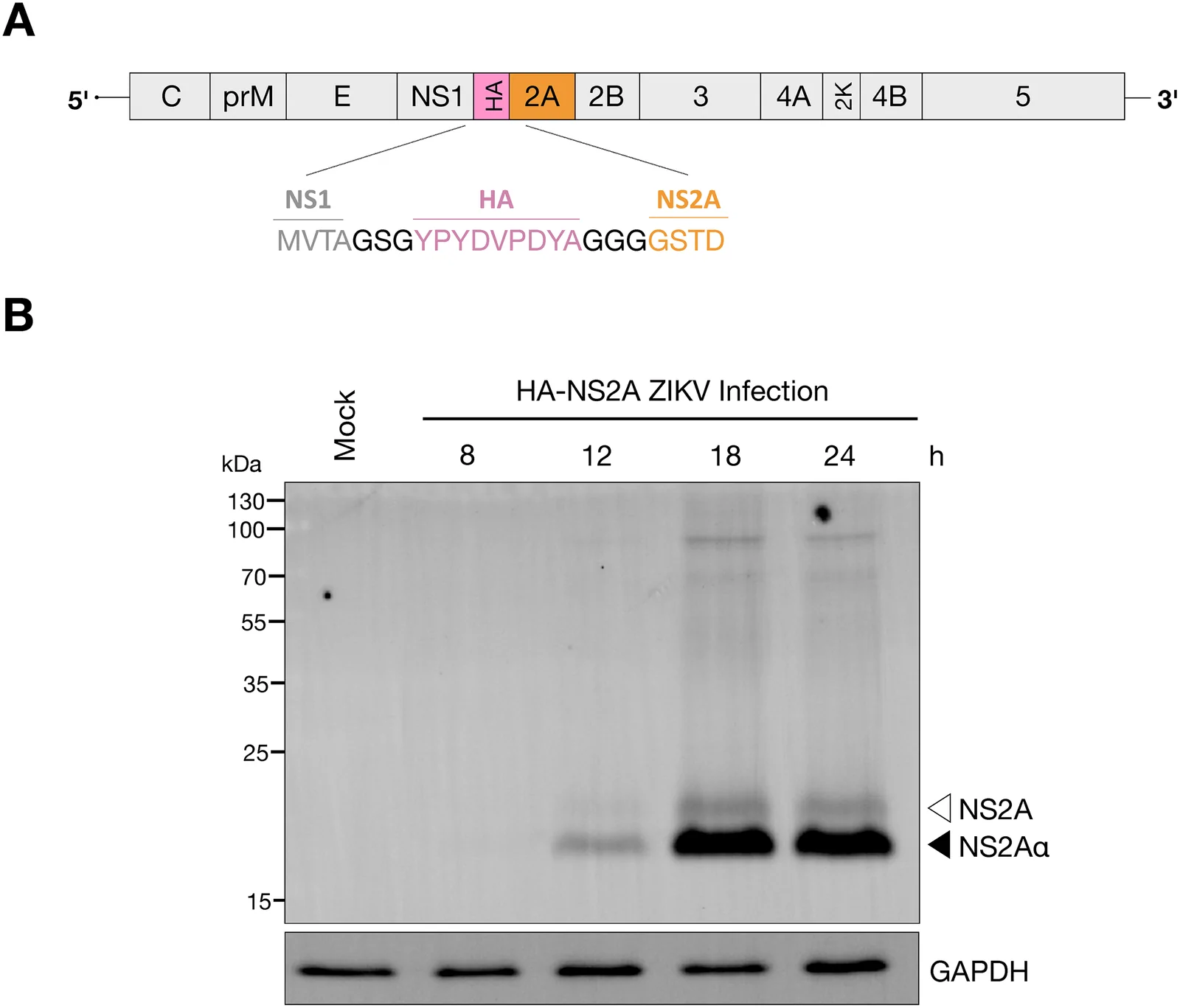
NS2Aα is the predominant isoform of NS2A during ZIKV infection. **(A)** Cartoon diagram of the ZIKV HA-NS2A genome, with inset showing the HA-tag. **(B)** Vero cells were infected with ZIKV (MOI = 1), total cell lysates were harvested at 8 – 24 h post-infection and analyzed by Western blot using anti-HA and GAPDH antibodies. h, hours post-infection.

### NS2Aα biogenesis is mediated by the NS3 protease at an internal protease cleavage motif

Examination of the viral NS3 serine protease cleavage motifs across the ZIKV polyprotein revealed that the consensus protease cleavage motif is [K/R]R↓[S/G] (Fig 2A-B). Inspection of the C-terminal region of NS2A, just upstream of the NS2A-2B cleavage site, allowed us to identify a similar motif, consisting of two positively-charged amino acids followed by a polar uncharged amino acid, _192_KK ↓ N_194_, which we reasoned may represent an NS3 protease cleavage motif (Fig 2B). To test whether this was the site of NS2Aα biogenesis and if cleavage was mediated by the NS3 protease, we generated several HA-tagged NS2A expression constructs, including full-length NS2A, the putative NS2Aα isoform (i.e., an NS2Aα “mimetic” containing a stop codon at position 194), as well as both WT and mutant NS2A-2B-3 polyproteins (Fig 2A). To verify that our *in vitro* expression constructs were of similar size to the NS2A protein species we observed during infection, we expressed WT NS2A-2B-3 in HEK 293T cells. We observed that WT NS2A-2B-3 produced two species, corresponding to full-length NS2A and the putative NS2Aα isoform, with the same molecular weights (~21 and 17 kDa) that we observed during infection (S3 Fig).

**Fig 2:**
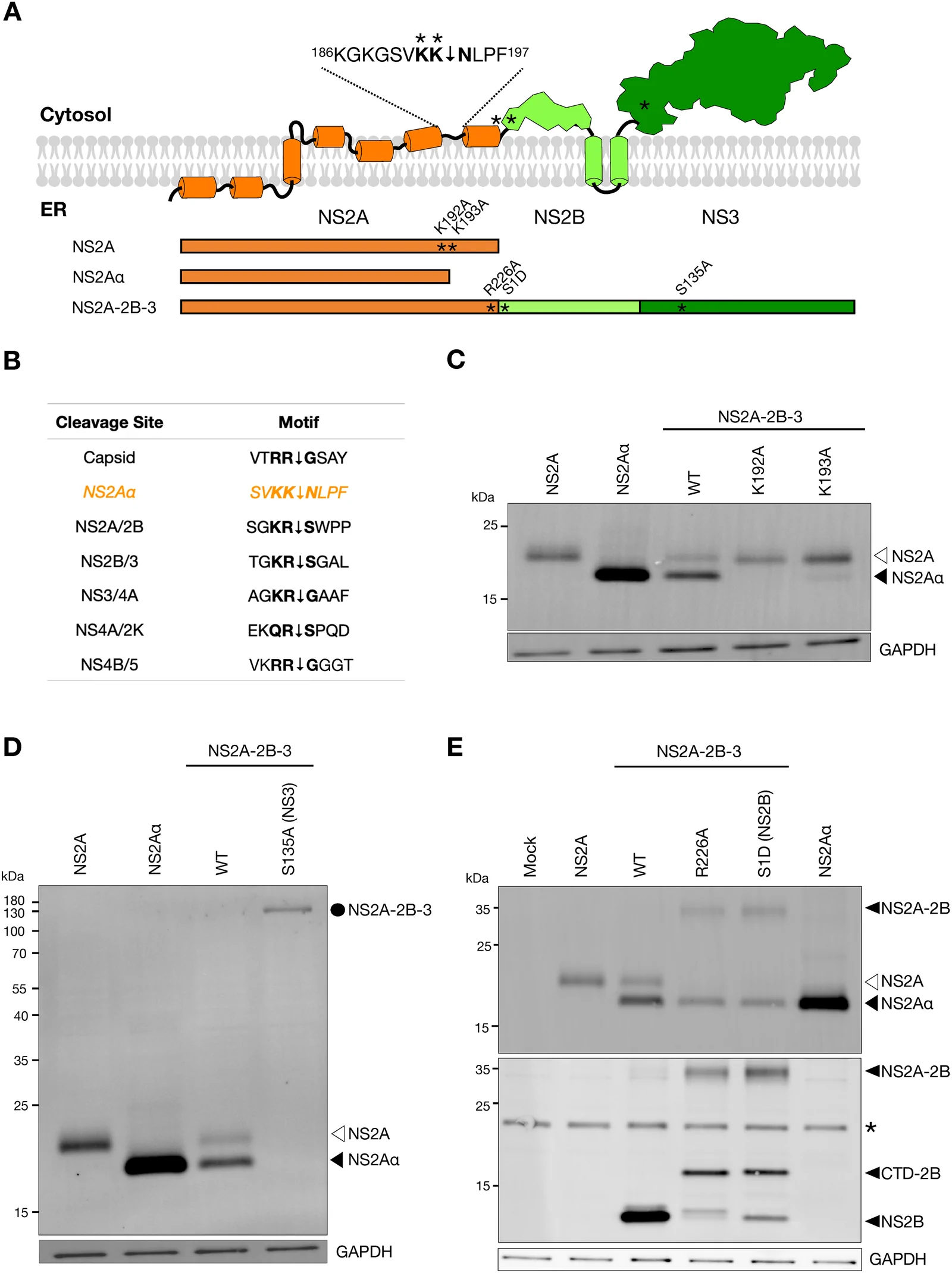
NS2Aα biogenesis is mediated by the NS3 protease at a non-canonical cleavage motif. **(A)** Previously proposed NS2A-2B-3 topology across the ER membrane [22], as well as NS2A, NS2Aα and NS2A-2B-3 expression constructs. Inset, sequence of the linker region of NS2A (amino acids 186-197) between the C-terminal cytosolic amphipathic helices. Asterisks (*) denote the locations of the NS2A (K192A, K193A, R226A), NS2B (S1D), and NS3 (S135A) mutations used herein. **(B)** NS3 protease cleavage motifs across the ZIKV^PR^ polyprotein. **(C-E)** NS2A, NS2Aα mimetic, as well as WT and mutant NS2A-2B-3 polyprotein expression constructs were introduced into HEK 293T cells and analyzed for NS2A expression at 24 h post-transfection via Western blot. Different NS2A and NS2B species (arrowheads) as well as the NS2A-2B-3 polyprotein (closed circle) are indicated. The asterisk (*) indicates a non-specific band. CTD, C-terminal domain.

To establish whether the putative NS2Aα cleavage motif (_192_KK ↓ N_194_) was required for NS2Aα biogenesis, we introduced mutations into the NS2A-2B-3 polyprotein at the two positively-charged residues in the P2 or P1 position of the NS2Aα cleavage site, respectively, i.e., K192A or K193A. In both cases, we observed a significant reduction in NS2Aα biogenesis, with K192A abolishing its production (Fig 2C). These results suggest that _192_KK ↓ N_194_ is likely the ZIKV NS2Aα cleavage motif. Nonetheless, to further implicate NS3 protease activity in NS2Aα biogenesis, we introduced the NS3 S135A mutation into our NS2A-2B-3 polyprotein, disrupting the active site of the protease (Fig 2D) [36,37]. As expected, the NS3 S135A mutation prevented NS3-mediated polyprotein processing and prevented NS2Aα production, suggesting that NS2Aα biogenesis is mediated by NS3 protease activity (Fig 2D).

Finally, we were curious whether NS2Aα biogenesis was dependent upon prior processing of the downstream NS2A-2B cleavage motif or if it could be processed independently. To this end, we introduced mutations into the NS2A-2B-3 polyprotein at the NS2A-2B cleavage motif, at position P1 (i.e., NS2A K226A) or P1´ (S1D NS2B), respectively (Fig 2E). In both cases, while NS2A-2B processing was impaired, we still observed production of NS2Aα (Fig 2E). Collectively, these results suggest that NS2Aα biogenesis occurs at an internal protease cleavage motif (_192_KK ↓ N_194_) in the C-terminal region of the protein, is dependent upon NS3 protease activity, and does not require prior NS2A-2B processing.

### The NS2Aα cleavage motif is conserved across the Orthoflavivirus genus

Since NS2Aα was the predominant isoform of the protein observed during ZIKV infection, and it had previously been observed in YFV, JEV and TMUV, we were curious whether the NS2Aα cleavage motif was conserved across the *Orthoflavivirus* genus [31,33,34,38]. To this end, we created an alignment and phylogenetic tree, using 62 viral polyprotein sequences, including those of the mosquito-borne (*Aedes* and *Culex* species), tick-borne, and no-known vector orthoflaviviruses (Fig 3A, S1 Table). In 61 of 62 orthoflaviviruses, we were able to unambiguously identify a putative NS2Aα cleavage motif (S1 Table). We then used the putative cleavage motifs to generate a sequence logo, which revealed that the NS2Aα cleavage motif has very strong (98.4%, *n* = 61) conservation for a lysine (K) residue at the P1 position (Fig 3B). This was in contrast with the preference of canonical NS3 protease cleavage motifs across the viral polyprotein (i.e., capsid, NS2A-2B, NS2B-3, NS3-4A, NS4A-2K, and NS4B-5) which have a strong preference for an arginine (R) residue at the P1 position (96.1%, *n* = 364) (Fig 3C). Moreover, we observed increased flexibility in the P1′ position of the NS2Aα cleavage motif, which also accommodated asparagine (N), histidine (H) and methionine (M) residues, in addition to the more canonical small hydrophobic amino acids, including glycine (G), threonine (T), alanine (A), and serine (S) (Fig 3B-C). Taken together, our phylogenetic and conservation analyses revealed that the NS2Aα cleavage motif is well-conserved across the mosquito-borne, tick-borne and no-known-vector orthoflaviviruses. Furthermore, the NS2Aα cleavage motif also appears to be somewhat unique with respect to the canonical NS3 cleavage motifs throughout the viral polyprotein.

**Fig 3:**
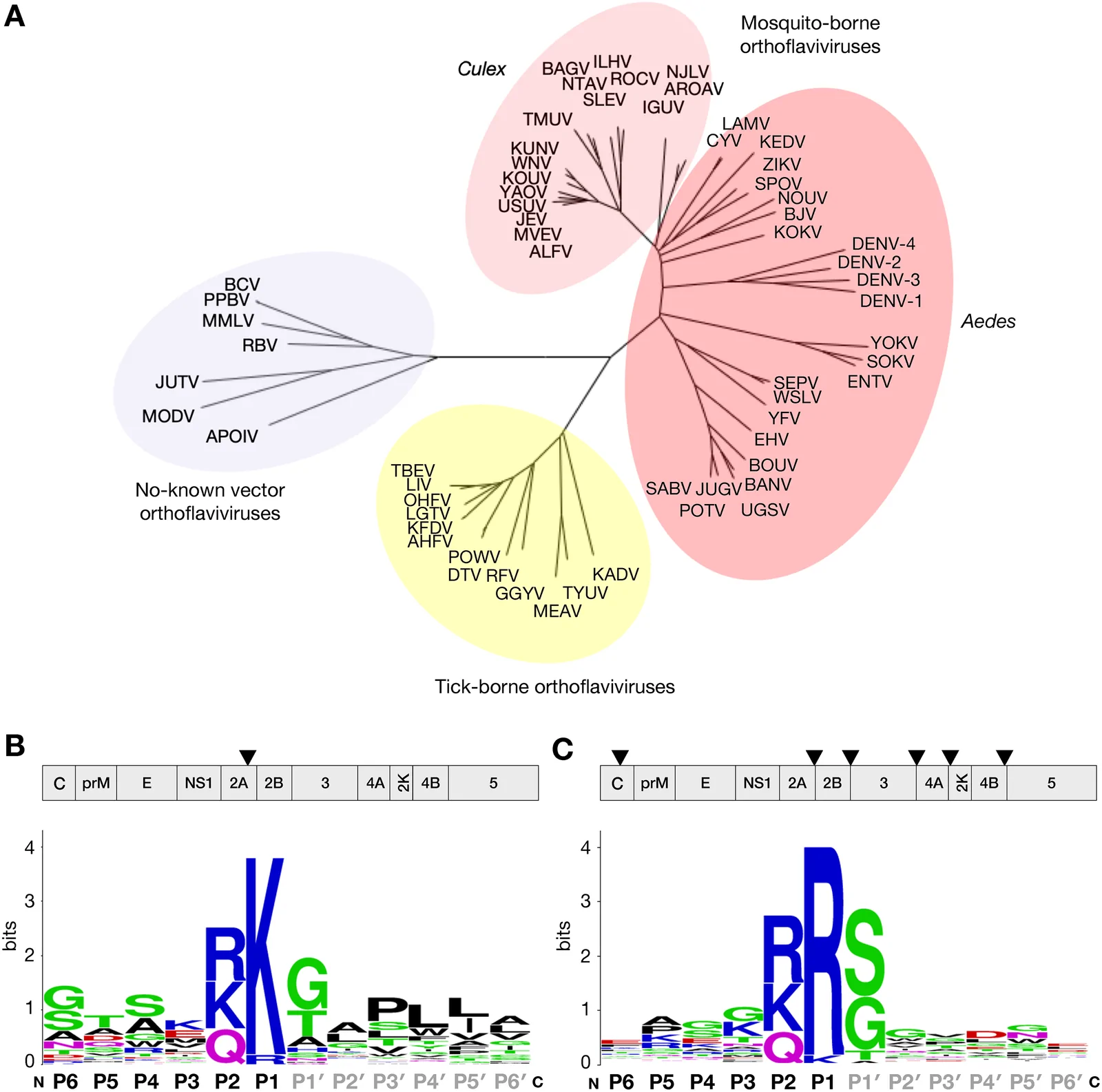
Evolutionary analysis across the *Orthoflavivirus* genus reveals unique nature of NS2Aα cleavage motif. **(A)** Phylogenetic tree based on the NS2A protein across the *Orthoflavivirus* genus. **(B)** Sequence logo of putative NS2Aα cleavage motif sequences (*n* = 61). **(C)** Sequence logo of canonical NS3-protease cleavage motifs throughout the orthoflavivirus polyprotein, including: capsid, NS2A-2B, NS2B-3, NS3-4A, NS4A-2K, and NS4B-5 (*n* = 364).

### NS2Aα biogenesis is NS3-mediated and can be observed across the Orthoflavivirus genus

While our conservation analyses suggested that the NS2Aα cleavage motif was conserved across the *Orthoflavivirus* genus, previous literature had cast doubts on the conservation of NS2Aα biogenesis [31,33,34,38]. For example, an earlier study that explored the topology of the DENV NS2A protein placed the putative NS2Aα cleavage site in the ER lumen [18]. Moreover, NS2Aα biogenesis during JEV infection was reported to be mediated by a host cell protease(s) [33]. Thus, to explore whether NS3-mediated NS2Aα biogenesis is conserved across the *Orthoflavivirus* genus, we generated HA-tagged NS2A as well as NS2A-2B-3 polyproteins for several orthoflaviviruses, including representatives of the mosquito-borne (*Aedes* and *Culex* species) and tick-borne clades. In all cases, we also generated a NS2A-2B-3 polyprotein containing a point mutation at the NS3 serine protease active site (S135A/S138A), creating a catalytically dead protease, to evaluate whether NS3 protease activity was important for NS2Aα biogenesis. The WT and mutant NS2A-2B-3 plasmids, in addition to NS2A alone, were introduced into HEK 293T cells and we assessed NS2Aα biogenesis via Western blot (Fig 4).

**Fig 4:**
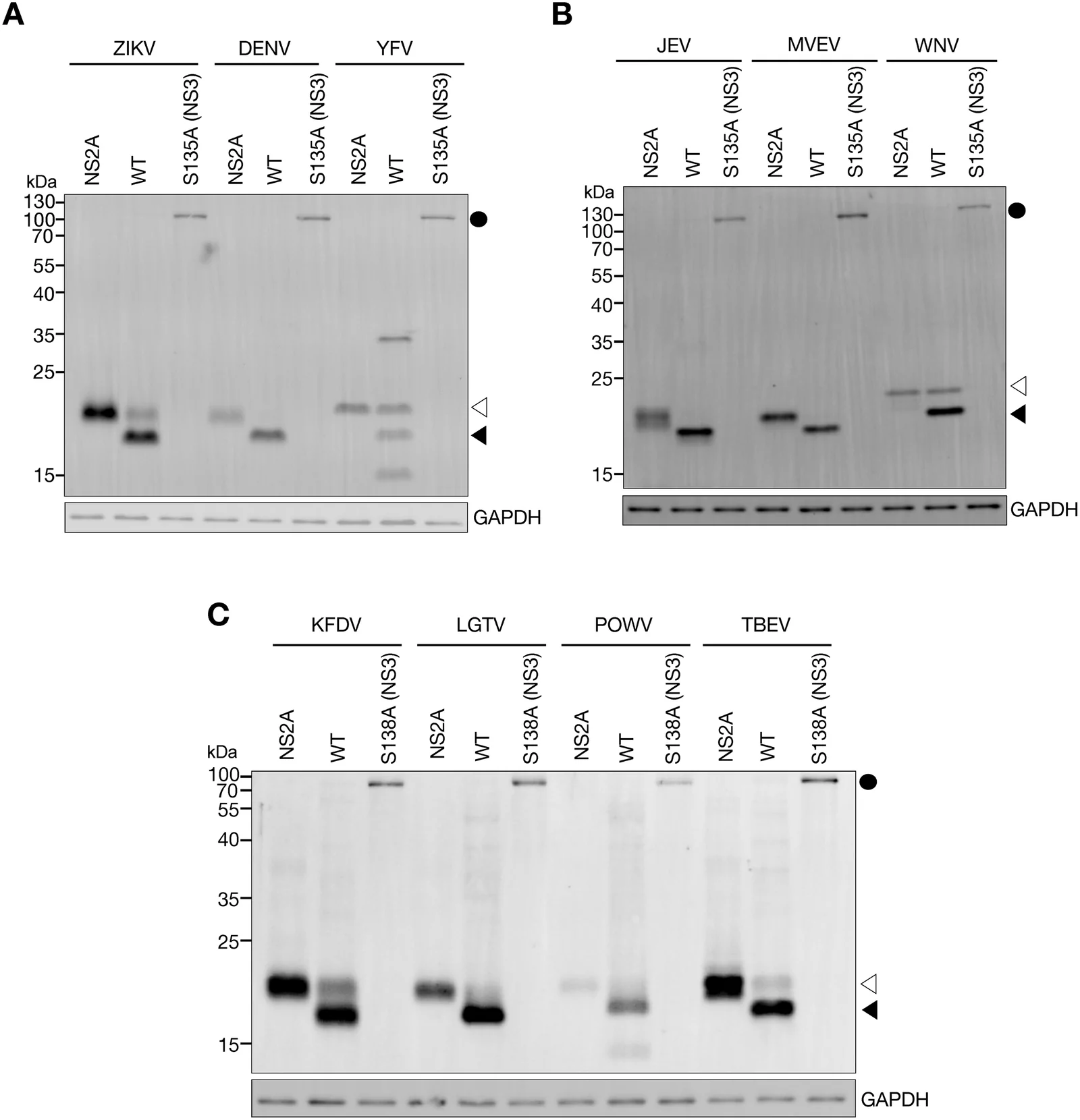
*In vitro* analysis of NS2A-2B-3 polyprotein expression across the *Orthoflavivirus* genus reveals conservation in NS2Aα biogenesis. Analysis of NS2Aα biogenesis in the: **(A)** *Aedes* species mosquito-borne (ZIKV, DENV2, YFV); **(B)** *Culex* species mosquito-borne (JEV, MVEV, WNV); and (C) tick-borne (KFDV, LGTV, POWV, TBEV) orthoflaviviruses. Total cell lysates were harvested 24 h post-transfection and probed for NS2A using an anti-HA antibody. Full-length NS2A (open arrowhead), NS2Aα (closed arrowhead), and the NS2A-2B-3 polyproteins (closed circle), are indicated.

Among the orthoflaviviruses tested, NS2Aα biogenesis was observed in all species transmitted by *Aedes* mosquitoes, despite reports that the DENV NS2A topology would not be conducive to its formation (Fig 4A) [18]. Furthermore, in all cases, NS3 protease activity was necessary for NS2Aα biogenesis (Fig 4A). Next, we explored *Culex* mosquito transmitted viruses, including JEV, Murray Valley Encephalitis virus (MVEV), and West Nile virus (WNV) (Fig 4B). As with the *Aedes*-borne orthoflaviviruses, in all cases we observed NS2Aα biogenesis was dependent upon NS3 protease activity. Interestingly, in the case of JEV and WNV, when NS2A was expressed alone, we also observed the presence of one or more smaller molecular weight species; however, none of these species appeared to be the same molecular weight of the NS2Aα isoform generated during expression of the NS2A-2B-3 polyprotein. As such, we postulate that these may be degradation products that are generated when NS2A is expressed outside the context of the viral polyprotein. Together, these results indicate that NS3-dependent biogenesis of NS2Aα can be observed for all mosquito-borne orthoflaviviruses tested herein.

Finally, we compared several of the more distantly related tick-borne orthoflaviviruses, including Kyasanur forest disease virus (KFDV), Langat virus (LGTV), Powassan virus (POWV), and tick-borne encephalitis virus (TBEV) (Fig 4C). Again, consistent with our previous results, in all cases we observed NS3-mediated NS2Aα biogenesis. However, similarly to what we observed in JEV and WNV, we also noted what appeared to be a decay product when TBEV NS2A was expressed outside of the NS2A-2B-3 polyprotein. Nonetheless, these results indicate that in all tested mosquito- and tick-borne orthoflaviviruses, NS2Aα biogenesis is conserved and is mediated by NS3 protease activity.

### ZIKV viral RNA replication requires an intact NS2A C-terminus

Next, we wanted to investigate the contribution of the different NS2A isoforms to specific stages of the viral life cycle, starting with viral RNA replication. To interrogate this, we generated several point mutations and a C-terminal domain (CTD) deletion in the context of an endogenously HA-tagged NS2A ZIKV subgenomic *Renilla* luciferase (RLuc) replicon system, alongside WT and replication-defective (GNN) positive- and negative-controls, respectively (Fig 5A-B) [39]. Importantly, in all cases, we verified that each of these mutants were able to undergo the expected polyprotein processing in the context of the NS2A-2B-3 polyprotein expression system (S4 Fig).

**Fig 5:**
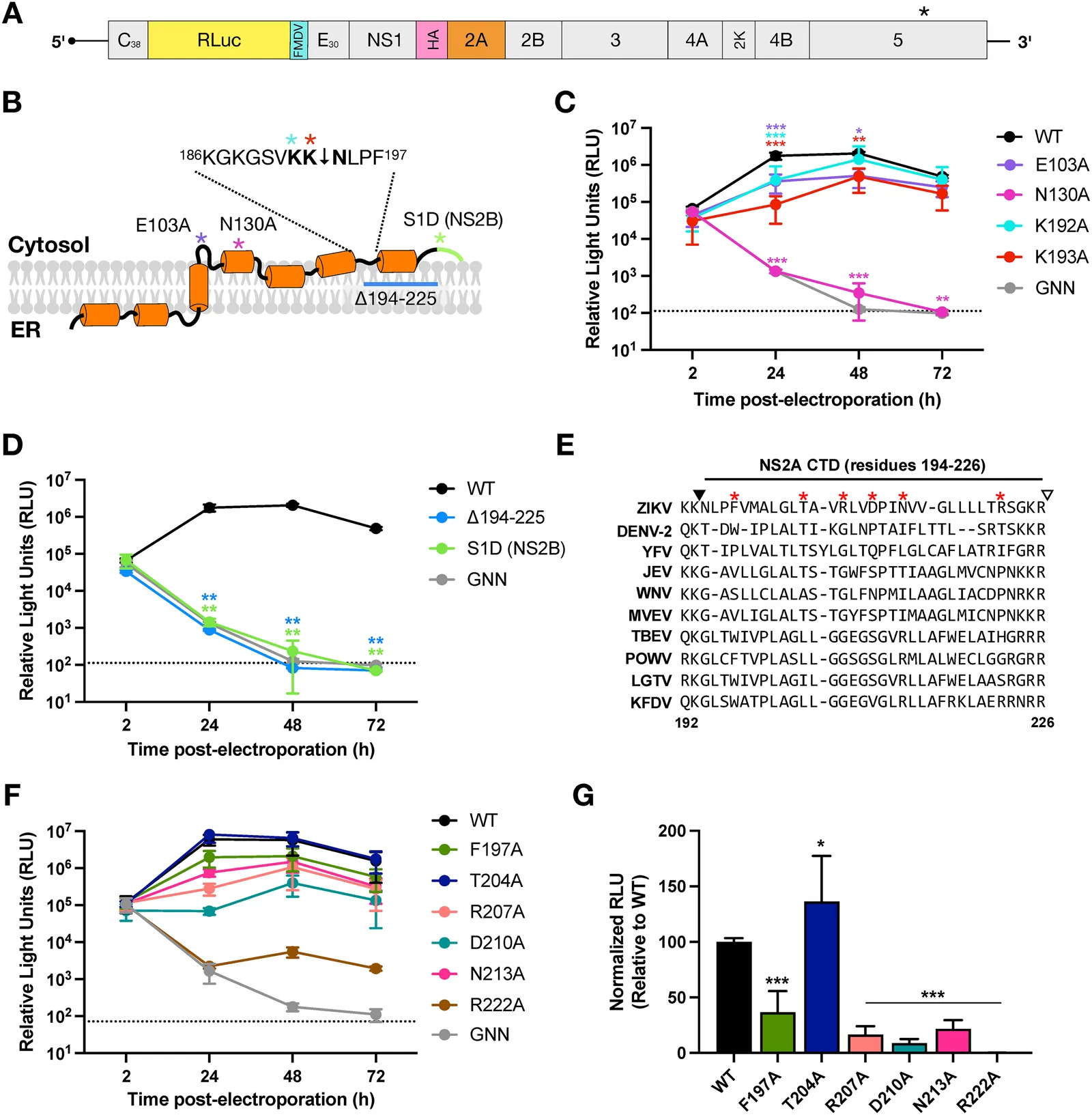
Full-length NS2A is required for viral RNA replication. **(A)** Schematic of HA-NS2A ZIKV^PR^ *Renilla* luciferase subgenomic replicon system. The asterick denotes the inactivating mutations in the polymerase active site residues (i.e., GNN). **(B)** Mutations in NS2A and NS2B shown in the context of the previously published membrane topology [22]. **(C-D)** Wild-type (WT), replication-defective (GNN), NS2A (E103A, N130A, K192A, K193A, Δ194-225) and NS2B (S1D) subgenomic replicon RNAs were electroporated into BHK-21 cells and luciferase activity was monitored between 2-72 h post-electroporation. **(E)** Sequence alignment of the NS2A C-terminal domain (CTD). Amino acids 192-226 are shown including the NS2Aα cleavage motif (_192_KK | N_194_, residues 192-194). The NS2Aα cleavage and NS2A-2B cleavage sites are denoted by closed and open arrowheads, respectively. Amino acids subjected to alanine mutagenesis are denoted by an asterisk. **(F-G)** NS2A C-terminal alanine mutant subgenomic replicon RNAs were electroporated into BHK-21 cells and luciferase activity was monitored between 2-72 h post-electroporation. **(G)** Luciferase activity at 72 h post-electroporation normalized to input (2 h timepoint) and represented as a percentage of WT. Statistical significance was determined using one-way ANOVA, *p ≤ 0.01, ** *p* ≤ 0.001, ***p ≤ 0.0001. Error bars represent standard deviation across three independent biological replicates. Lower limit of detection is indicated (dashed line).

To test the importance of NS2Aα in viral RNA replication, we introduced mutations that impair NS2Aα biogenesis by disrupting the NS3 cleavage motif (K192A and K193A) (Fig 2C and 5B-C). While in both cases we observed a slight delay in viral replication, both K192A and K193A accumulation did not differ significantly from the WT subgenomic replicon by 72 h post-electroporation (Fig 5C). As additional protein-specific controls, we generated NS2A mutants N130A and E103A, which were previously shown to specifically impair viral RNA replication and virion assembly, respectively, and behaved as expected (Fig 5B-C) [21,22]. Taken together, these results suggest that NS2Aα biogenesis is not required for efficient viral RNA replication.

To determine the contribution of the NS2A CTD to viral RNA replication, we generated the NS2B S1D mutation, which impairs NS2A-2B cleavage, but retains the ability to generate NS2Aα, leaving the NS2A CTD (amino acids 194 – 226) fused to NS2B (Fig 5B,D and S4 Fig). This mutation completely abolished viral RNA accumulation, like the replication-defective (GNN) replicon RNA (Fig 5D). However, as this mutant also generates a fusion product between the NS2A CTD and NS2B, we also generated an NS2A CTD deletion (Δ194–225), which removes the majority of the NS2A C-terminus altogether, but retains the ability to undergo polyprotein processing at the NS2Aα-2B junction (Fig 5B,D and S4 Fig). Similarly, we observed that Δ194–225 also completely abolished viral RNA accumulation (Fig 5D). These results indicate that the NS2A CTD is important for viral RNA replication in the ZIKV subgenomic replicon system.

Finally, given that the NS2A CTD has relatively low sequence conservation across orthoflaviviruses, we explored the importance of the amino acid composition of the CTD for viral RNA accumulation (Fig 5E-G). To this end, we selected several residues for alanine mutagenesis, including F197, T204, R207, D210, N213 and R222, while generally omitting aliphatic amino acids and those in close proximity to the NS2A-2Aα and NS2A-2B cleavage motifs (Fig 5E-G). Interestingly, except for the T204A mutation, which replicated slightly better than the WT subgenomic replicon, all other mutations, including F197A, R207A, D210A, N213A and R222A, significantly impaired viral RNA replication (Fig 5F-G). Collectively, this data indicates that several residues in the ZIKV NS2A CTD are important for efficient viral RNA accumulation.

In sum, our results in the subgenomic replicon system imply that internal processing of ZIKV NS2A at the NS2Aα cleavage site are dispensable for viral RNA accumulation. In contrast, we observed that full-length NS2A, and several amino acid residues in the NS2A CTD, are required for efficient viral RNA replication.

### The NS2A CTD is not required for vRO biogenesis, but is important for vRO morphology

Importantly, it is not yet clear whether NS2A is important for vRO biogenesis (a process that occurs before or concurrent with negative-strand RNA synthesis) or if it plays a distinct role in viral RNA replication. To this end, we generated the same NS2A mutations and/or deletion used in the subgenomic replicon in the ZIKV plasmid-induced vRO (pIRO-Z) system. While this system encodes the ZIKV^H/PF/2013^, the amino acid sequence of NS2A is identical to that of ZIKV^PR^ used herein (Fig 6A) [40]. Importantly, this system can be used to evaluate vRO biogenesis in the absence of active viral RNA replication [40–44].

**Fig 6:**
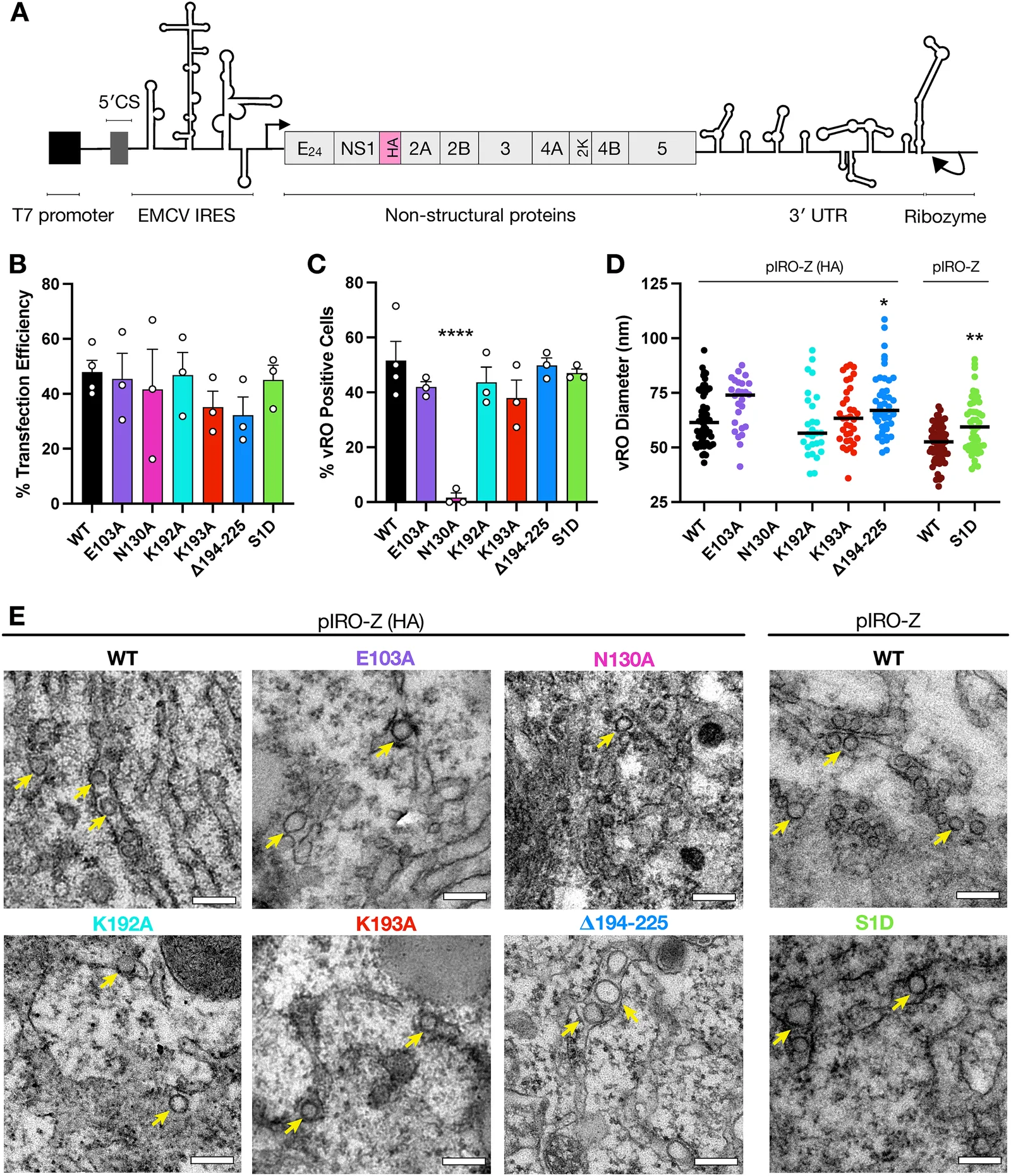
Full-length NS2A is required for efficient vRO biogenesis. **(A)** Cartoon diagram of pIRO-Z system. **(B)** Transfection efficiency, (C) percentage of vRO positive cells, and (D) vRO diameter based on immunofluorescence staining for viral NS3 and transmission electron tomography (TEM) imaging of pIRO-Z-transfected cells. (E) pIRO-Z-transfected cells were fixed at 18 h and processed for TEM analysis. Arrowheads indicate individual ROs. Scale bar = 200 nm. Error bars represent the standard error of the mean (SEM) of three independent biological replicates.

Interestingly, while the addition of the HA-tag to NS2A did not significantly impact transfection efficiency (Fig 6B), or the percentage of vRO positive cells observed via electron microscopy (Fig 6C), we observed that vROs were consistently larger, with an average diameter of 61.5 vs. 59.4 nm, for pIRO-Z (HA) and pIRO-Z (WT), respectively (Fig 6D-E). Consistent with our findings in the subgenomic replicon system, we observed that the HA-tagged K192A and K193A mutants both had a similar percentage of vRO positive cells and vROs were of similar size to their respective pIRO-Z (HA) control (Fig 6B-E). In agreement with previous results, we also observed that the HA-tagged N130A mutant was severely attenuated in vRO biogenesis with only 1.7% vRO positive-cells compared with the control, while vRO-like structures of similar size were still readily observed with the HA-tagged E103A mutant (Fig 6B-E). Importantly, we observed similar transfection efficiencies across the pIRO-Z constructs and the pattern of polyprotein processing was consistent with our previous results using the NS2A-2B-3 polyprotein (Fig 6B and S4-S5 Fig). These results suggest that while NS2A is important for vRO formation, NS2Aα biogenesis is dispensable for this process.

Given that NS2Aα biogenesis was dispensable for vRO formation, we next wanted to understand the contribution of the NS2A CTD to vRO biogenesis. To this end, we used the NS2B S1D mutant, which retains the ability to form NS2Aα, but impairs NS2A-2B processing, as well as the NS2A Δ194–225 CTD deletion (Fig 6B-E). Interestingly, while the NS2B S1D pIRO-Z mutant used herein did not contain an HA-tag, we observed that the vROs had a significantly larger average diameter and appeared more amorphous in shape, when compared with their pIRO-Z (WT) counterpart (Fig 6B-E). Similarly, the NS2A Δ194–225 deletion also resulted in larger and more amorphous vROs when compared to its pIRO-Z (HA) counterpart (Fig 6B-E). In both cases, we observed similar transfection efficiencies and proportion of vRO positive cells, and polyprotein processing patterns were consistent with our previous results using the NS2A-2B-3 polyprotein (Fig 6E and S4-S5 Fig). These results suggest that full-length NS2A is important for maintaining the morphology and organization of vROs.

Taken together, while the N130A mutant impaired vRO biogenesis, all other NS2A mutations and/or truncations were competent in vRO formation, indicating that while NS2A is important for vRO biogenesis, NS2Aα biogenesis is not necessary for this process. However, given that NS2A Δ194–225 and NS2B S1D resulted in vROs with altered morphology, our results suggest that full-length NS2A is important for the organization of vROs.

### Mutations that disrupt NS2Aα biogenesis impair viral spread

Having established that full-length NS2A, but not NS2Aα, is required for vRO biogenesis and viral RNA replication, we next wanted to evaluate the importance of the two different NS2A isoforms in virion assembly. To do so, we introduced our replication-defective (N130A) and assembly-defective (E103A) controls, as well as our NS2Aα biogenesis mutants, K192A and K193A, into the HA-NS2A ZIKV genomic RNA. *In vitro* transcribed full-length genomic RNAs were electroporated into BHK-21 cells and viral spread was assessed via immunofluorescence, while infectious particle production was assessed via plaque-forming unit (PFU) assay on naïve Vero cells (Fig 7). Electroporation of the WT (HA) genomic RNA resulted in accumulation of HA-NS2A positive cells from 24-72 h and we observed titers of 4.93 ± 2.7 × 10^3^ at 96 h post-electroporation. As expected, we did not observe any HA-NS2A positive cells or plaques after introduction of the replication-defective N130A mutant (Fig 7). The E103A mutant displayed HA-NS2A-positive cells at 24 h post-electroporation; however, consistent with a lack of virion assembly and viral spread, we only observed a few positive cells 48–72 h post-electroporation and significant impairment of infectious particle production (<20 PFU/mL, Fig 7). Similarly, the introduction of the NS2Aα cleavage site mutants (K192A and K193A) resulted in a reduction in HA-NS2A-positive cells over time and no viral plaques were detected at 96 h post-electroporation (Fig 7). Taken together, these findings suggest that while NS2Aα biogenesis is dispensable for viral RNA replication, mutations that impair NS2Aα cleavage are defective in infectious particle production and cell spread.

**Fig 7:**
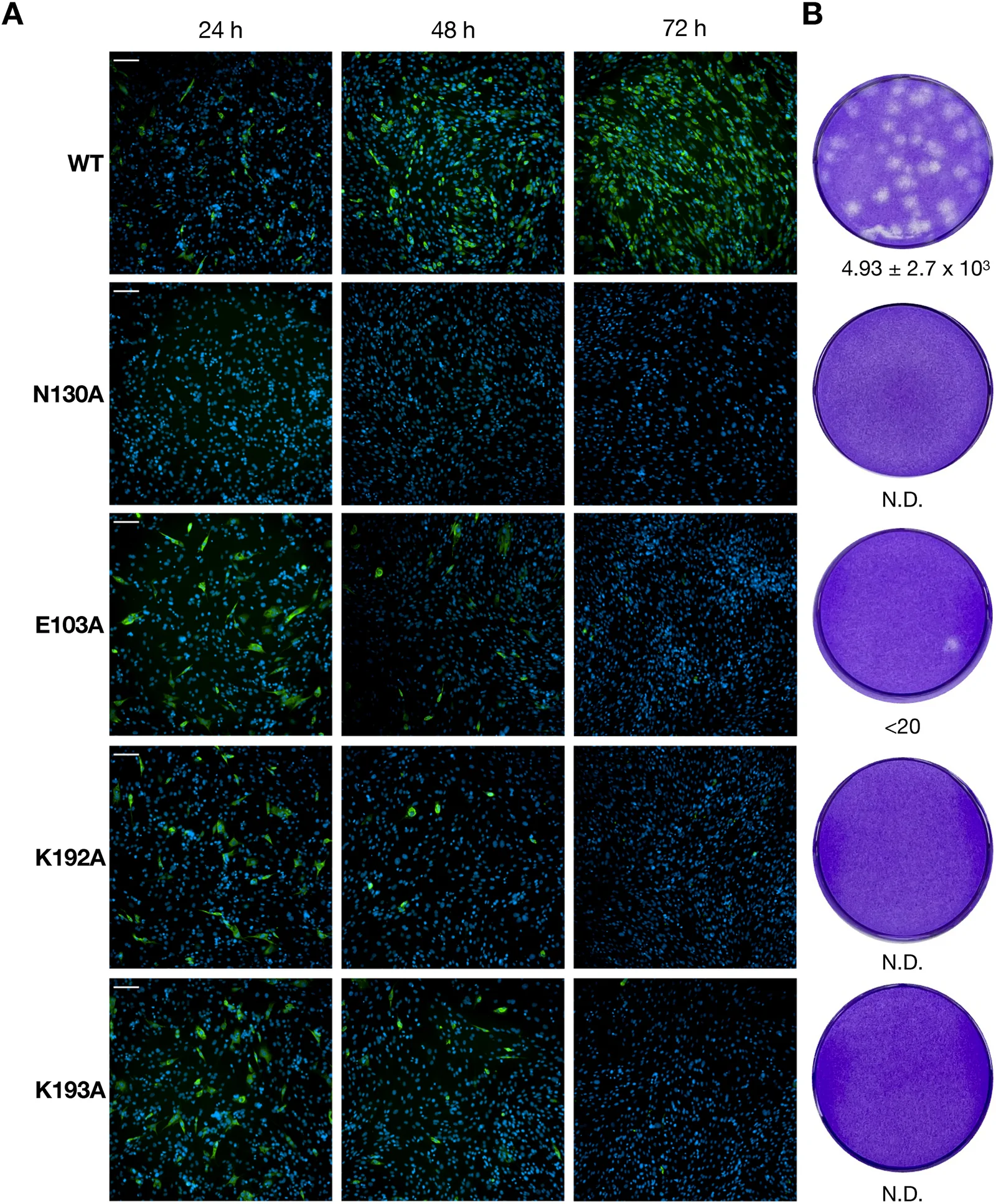
NS2Aα biogenesis is important for viral spread and infectious particle production. **(A)** HA-NS2A ZIKV genomic RNAs containing the indicated mutations were electroporated into BHK-21 cells. Cells were immunostained at the indicated timepoints and monitored for viral spread using an anti-HA antibody. Scale bar = 10 µm. **(B)** Supernatants were harvested at 4 days post-electroporation and PFU assays were performed in Vero cells to monitor infectious particle production. Representative plaque assays from the 10^-2^ dilution are shown. Mean viral titers were calculated across three biological replicates at the 96 h timepoint. The limit of detection (LOD) is 20 PFU/mL. Replicates with no plaques detected are reported as none detected (N.D.).

To further probe whether the defects we observed in infectious particle production were due to a lack of particle production or issues in the specific infectivity of viral particles (i.e., non-infectious particle production), we electroporated viral genomic RNAs into Vero cells and assessed the amount of viral genomic RNA in cell supernatants at 38 h post-electroporation (S6A Fig). While genomic RNA was readily detectable in the cell supernatants of WT electroporated cells, we were unable to detect viral genomic RNAs in any of the mutant cell supernatants at a level that was significantly greater than that detected in the non-replicative control (N130A) (S6A Fig). Furthermore, while envelope and capsid proteins were readily detected in the cell lysates and supernatant of WT electroporated cells, we did not detect either of these proteins in the cell supernatants of any of the NS2A mutants (S6B Fig). However, viral replication was clearly established in these cells as the capsid and envelope proteins were detectable in the cell lysates of all the replication-competent mutants (i.e., E103A, K192A and K193A), but not the sole replication-defective N130A mutant (S6B Fig). Taken together, these results suggest that the defects we observed in cell spread and infectious particle production are likely a result of a defect in viral genome packaging – ultimately resulting in a reduction in infectious particle production, rather than a defect in the specific infectivity of viral particles.

## Discussion

In this study, we sought to provide a detailed characterization of the contributions of the ZIKV NS2A protein to the viral life cycle. Firstly, we demonstrated that both full-length NS2A as well as a C-terminally truncated isoform, NS2Aα, are produced during ZIKV infection, with the latter representing the predominant NS2A isoform. Moreover, we identified the NS2Aα cleavage motif, located in the C-terminus of the protein at position _192_KK ↓ N_194_. Despite the somewhat noncanonical nature of this motif, we found that NS2Aα biogenesis was mediated by the viral NS3 protease. Additionally, NS2Aα biogenesis did not require prior NS2A-2B cleavage, suggesting that this event can occur independently from complete processing of the NS2A-2B-3 polyprotein.

In our efforts to expand our understanding of NS2Aα conservation beyond ZIKV, our phylogenetic analyses indicated that the putative NS2Aα cleavage site is conserved throughout the *Orthoflavivirus* genus. Moreover, this motif appears to be unique with respect to the canonical NS3 protease cleavage motifs throughout the orthoflavivirus polyprotein. More specifically, the P1 position strongly favored a lysine (K) residue rather than an arginine (R), while we also observed more flexibility in the P1´ position, which could include asparagine (N), histidine (H) and methionine (M) residues, in addition to the more canonical glycine (G), threonine (T), alanine (A), and serine (S) residues. This was striking given that previous *in vitro* analyses of the NS3 protease, with its NS2B cofactor, suggested a preference for an arginine at P1, while substitution with a lysine residue led to significantly reduced affinity [30,45]. However, it is possible that the combination of residues in this motif, including the P1´ position, is important for altering enzyme affinity at this site. While the reason for the somewhat noncanonical nature of the NS2Aα cleavage motif is not yet clear, the strong conservation of the P1 position suggests that there may be some importance to this specific combination of residues for NS2Aα’s activities.

To provide experimental support that the NS2Aα isoform was actively generated across the *Orthoflavivirus* genus, we expressed NS2A alone and within the NS2A-2B-3 polyprotein from several of the mosquito-borne (*Aedes* and *Culex*) and tick-borne orthoflaviviruses. While we occasionally observed that expression of NS2A alone resulted in additional decay products not observed during expression of the NS2A-2B-3 polyprotein, in all cases we observed NS3-mediated NS2Aα biogenesis. It is possible that expression of NS2A alone does not result in proper folding and/or topology of NS2A on the ER membrane, resulting in host protease-mediated decay intermediates; however, the precise reason(s) for the biogenesis of these lower molecular weight species will require further research. Furthermore, in accordance with what we observed in ZIKV, NS2Aα tended to be the predominant isoform produced; however, it remains to be seen whether this will hold true during viral infection. Notably, in a few cases, we also observed larger (YFV) or smaller (YFV and POWV) HA-tagged species during expression in the context of the NS2A-2B-3 polyproteins (Fig 4A and C). The larger molecular weight species observed in YFV is consistent with incomplete polyprotein processing at the NS2A-2B junction (~37.9 kDa); while the smaller species are consistent with the observation of a potential upstream NS3 protease cleavage motif in both YFV (_142_RK ↓ A_144_) and POWV (_98_KR ↓ V_100_) NS2A, which would be predicted to generate products consistent with these low molecular weight species (15.4 and 10.9 kDa, respectively). However, whether these NS2A species are observed during infection will require further investigation. Nonetheless, our results suggest that the biogenesis of the NS2Aα isoform is mediated by NS3 protease activity and that its production is conserved across the *Orthoflavivirus* genus.

Beyond characterization of NS2Aα conservation and biogenesis, we were also interested in investigating whether the two NS2A isoforms played disparate roles in the orthoflaviviral life cycle. First, we explored the NS2A mutants in the context of active viral RNA replication, using the subgenomic replicon system. In this system, N130A completely abolished viral replication, while the K192A and K193A mutations had modest effects on viral RNA accumulation, suggesting that NS2Aα biogenesis is dispensable for this process. Conversely, when we precluded the formation of full-length NS2A (NS2B S1D and NS2A Δ194–225), we found that viral RNA replication was completely abolished. Further alanine mutagenesis in the NS2A CTD revealed the importance of several amino acids in this region for efficient viral RNA replication. Interestingly, this finding is supported by previous mutational analyses in both DENV and ZIKV NS2A, where mutation of several residues in the CTD were found to significantly impair viral RNA replication [19,22,23].

Interestingly, while NS2A has been previously implicated in membrane rearrangement, it has yet to be evaluated for a role in vRO formation [26,27]. Thus, we also explored the importance of NS2A in vRO biogenesis using the pIRO-Z system [40,43,44]. Consistent with our results from the subgenomic replicon, we found that the N130A mutation attenuates ZIKV vRO biogenesis, supporting a role for NS2A in this process. However, all other mutations we explored, including those that impair NS2Aα biogenesis, were still capable of vRO formation. This implies that while NS2A is important for vRO formation, NS2Aα biogenesis is dispensable for this process. However, while precluding the formation of full-length NS2A still resulted in vRO formation, both the NS2B S1D mutant and the NS2A Δ194–225 deletion resulted in larger and more amorphous vROs. These results suggest that NS2A plays an important role in vRO architecture, and that the NS2A CTD is meaningful to its organization. It is possible that the NS2A CTD is important for facilitating the proper folding and/or topology of NS2A or for mediating host or viral protein-protein interactions necessary for RO biogenesis. Additionally, given the net positive charge of this region of NS2A, it is also possible that these residues may facilitate NS2A-viral RNA interactions in the context of the vRO. However, further insights into the precise role(s) of these specific residues in viral RNA replication will require further research. Nonetheless, these findings support a role for NS2A in vRO biogenesis and suggest that specific residues within this non-enzymatic orthoflavivirus protein can uncouple vRO biogenesis from active viral RNA replication, indicating that these two processes may have distinct requirements for NS2A during the viral life cycle.

In contrast to our finding that NS2Aα was dispensable for viral RNA replication, we found that inhibition of NS2Aα biogenesis completely abolished viral spread and infectious particle production. Moreover, we were unable to detect significant levels of viral genomic RNAs in the cell supernatants of any of our NS2Aα biogenesis mutants. These finding highlight the importance of NS2Aα biogenesis for virion assembly and suggest a role for NS2Aα in genome packaging given that we were unable to detect genomic RNAs in the cell supernatants of these mutants. Given that NS2A has previously been shown to interact with the 3´ UTR of the viral genome, it is possible that the NS2Aα isoform plays an important role in recognition of the viral genomic RNA for packaging during virion assembly [20,21]. Notably, a similar phenotype was also observed in YFV, where mutations that impaired NS2Aα biogenesis also impaired infectious particle production [31,32]. In this case, NS2Aα biogenesis mutants were associated with an increase in non-infectious subviral particle production (i.e., particles lacking a nucleocapsid), arguing that loss of NS2Aα may uncouple genome packaging (i.e., nucleocapsid formation) and viral envelope protein (prM and E) assemblies [31]. However, herein we were unable to detect viral envelope or capsid proteins in electroporated cell supernatants of any of the NS2Aα biogenesis mutants, suggesting that there may not be a similar global increase in subviral particle production. Nonetheless, it is possible that subviral particles were simply below the level of detection and thus more in-depth analyses will be required to resolve whether changes in NS2Aα biogenesis alter the production of ZIKV subviral particles as previously observed in YFV [31]. Interestingly, a recent study also suggested that DENV and ZIKV NS2A interacts with UFL1, a UFMylation E3 ligase, whose knockdown impairs infectious virion production [46]. It is thus tempting to speculate that this observation may be related to post-translational modification of the NS2Aα isoform; however, further research will be needed to verify whether either of the NS2A isoforms are post-translationally modified and how this impacts viral particle production. Nonetheless, the results described herein support a role for the NS2Aα isoform in orthoflavivirus infectious particle production and suggest a specific role for NS2Aα in viral genome packaging.

In conclusion, we provide an in-depth characterization of the ZIKV NS2A and NS2Aα isoforms and demonstrate that the expression of NS2Aα is conserved across the *Orthoflavivirus* genus. Additionally, we provide evidence that the two NS2A isoforms play disparate and mutually exclusive roles in the viral life cycle, with full-length NS2A and the NS2Aα isoform supporting viral RNA replication and virion assembly, respectively. Additionally, we also provide the first direct evidence that NS2A is important for vRO biogenesis and its architecture. Future studies will be focused on providing more precise mechanistic insight into how NS2A and the NS2Aα isoform facilitate viral RNA replication and virion assembly, respectively.

## Materials and methods

### Cells and viruses

Baby hamster kidney (BHK-21) cells, African green monkey kidney (Vero) cells, human embryonic kidney (293T) cells, and human lung carcinoma (A549) cells were all maintained in tissue-culture treated flasks (Sarstedt) and grown in complete media, consisting of Dulbecco’s modified Eagle’s medium (DMEM; Gibco) supplemented with 10% fetal bovine serum (FBS, Gibco), 1% nonessential amino acids (Gibco), 1% l-glutamine (Gibco) and maintained at 37°C/5% CO_2_. C6/36 cells were maintained in EMEM supplemented with 10% fetal bovine serum (FBS, Gibco), 1% nonessential amino acids (Gibco), 1% l-glutamine (Gibco), 15 mM HEPES and 28°C/5% CO_2_. Huh-7/Lunet-T7 cells were maintained in complete media further supplemented with 100 U/mL penicillin and 100 μg/mL streptomycin. During Huh7/Lunet-T7 maintenance, 5 μg/mL Zeocin was also added to the media to maintain stable T7 expression. Cells were routinely screened for mycoplasma.

The ZIKV PRVABC-59 infectious cDNA (ZIKV^PR^; GenBank accession: KX377337) was kindly provided by Young-Min Lee (Utah State University) [47]. ZIKV^PR^ and HA-NS2A ZIKV^PR^ viral stocks were generated by electroporation of Vero cells with *in vitro* transcribed genomic RNA as previously described [48]. Viral stocks were titrated by plaque forming unit (PFU) assay in Vero cells.

### Viral infections

HA-NS2A ZIKV^PR^ infections were carried out using the indicated multiplicity of infection (MOI) in Eagle’s minimum essential medium (EMEM; Gibco). Virus was added to subconfluent monolayers of the specified cells for 2 hours (h) at 37 °C/5% CO_2_ to promote viral binding. At 2 h post-infection, supernatants were removed and were replaced with fresh DMEM media supplemented with 15 mM HEPES (Wisent, Inc.) and 2% FBS.

### Molecular cloning

The sequences of all primers used for cloning are listed in S2 Table. Using a pCDNA3 plasmid containing nucleotides 3435–5861 of the ZIKV^PR^ infectious cDNA and *ApaLI* and *SfoI* restriction sites, the HA-tag at the N-terminus of NS2A was introduced using Quikchange II XL site-directed mutagenesis kit (Agilent) following the manufacturer’s instructions and using primers ZIKV-HA-NS2A-FOR and ZIKV-HA-NS2A-REV. *ApaLI* and *SfoI* restriction enzymes (NEB) were then used to clone this region back into the ZIKV^PR^ infectious cDNA encoded within a bacterial artificial chromosome (BAC). The specified NS2A and NS2B mutations were introduced into the subcloned plasmid via reverse PCR with Q5 polymerase [New England Biolabs (NEB)]. The mutated region was amplified with BAC-GA-FWD/REV primers and introduced into digested cDNA BAC (*ApaLI* and *SfoI*) via Gibson assembly (Takura Bio). Resulting ZIKV^PR^ cDNA BAC were purified using the QIAGEN Large-Construct kit (Qiagen), and sequences were verified by Oxford Nanopore sequencing (Plasmidsaurus).

The introduction of the HA-tag at the N-terminus of NS2A in the previously described ZIKV^PR^ subgenomic *Renilla* luciferase (RLuc) reporter replicon system was completed in the same manner as described above for the infectious cDNA [39]. Overexpression vectors containing the HA-tagged NS2A-2B-3 polyprotein were created in the pCDNA3.1 backbone. The *Gaussia* luciferase signal sequence (SPG), the C-terminal 16 aa of NS1 (subsequently removed), and the N-terminal HA-tag on NS2A was introduced using a gBlock (S3 Table) and overlapping PCR with the NS2A-NS3 region of the ZIKV^PR^ infectious cDNA, using NheI-MreI-NS2A-FOR and NS3-stop-XhoI-Rev primers. As such, the final construct contained the SPG signal sequence with a GSG linker leading into the HA-tag on the N-terminus of NS2A-NS2B-NS3 (NS2A-2B-3). Mutations in ZIKV NS2A (E103A, N130A, K192A, K193A, Δ194–225), NS2B S1D or NS3 (S135A) were made using reverse PCR with Q5 polymerase (NEB) with the specific primers listed in S2 Table. A version of the overexpression construct was also created that removed NS2B and NS3 to express full-length NS2A on its own using Δ2B-3-FWD and Δ2B-3-REV.

For introduction of NS2A mutants into the pIRO-Z system, a subclone using the pCDNA3.1 backbone was generated using Gibson assembly (Takara Bio), encompassing nucleotides 3716–6571 of the ZIKV^H/PF/2013^ genome. NS2A (E103A, N130A, K192A, K193A, Δ194–225) or S1D NS2B mutants were generated in the subclone using reverse PCR (S2 Table) with Q5 polymerase (NEB). Subcloned fragments were digested using *KpnI* and *BamHI* (NEB) and ligated back into the pIRO-Z backbone vector using T4 DNA ligase (NEB).

To clone the different orthoflaviviral HA-NS2A, HA-NS2A-2B-3, and HA-NS2A-2B-3 (S135A/S138A) overexpression vectors viral templates were obtained: ZIKV PRVABC-59 (KU501215.1), DENV-2 16681 (KU725663.1), YFV 17D-204 (MN106241.1), JEV CNU/LP2 (AY585243.1), WNV NY99 (KC407666.1), MVEV Canada/human/2011 (KX229766.1), TBEV SofjinKSY (JF819648.2), POWV LEIV-5530 (OP037823.1), LGTV attenuated strain E5 (AF253420.1), and KFDV P9605 (JF416958.1). The appropriate NS2A-2B-3 sequences were inserted into the pCDNA3.1 backbone using Gibson assembly (Takara Bio) containing an N-terminal HA-tag. Following generation of the various orthoflaviviral NS2A-2B-3 constructs, we created both HA-NS2A constructs via reverse PCR and introduced the NS3-proteolytically inactivating mutations (S135A/S138A) via site-directed mutagenesis. All sequences were confirmed by Sanger Sequencing (University of British Columbia, Sequencing + Bioinformatics Consortium).

### Transfection of NS2A expression plasmids

One day prior to transfection, 6-well plates were seeded at a density of 1.1 × 10^6^ HEK 293T cells/well. Transfection complexes were prepared in OPTI-MEM (Gibco) using 1–6 µL of Lipofectamine 2000 (Thermo Fisher) per well and transfected according to the manufacturer’s instructions. At 4 h post-transfection, the transfection complexes were removed and replaced with complete DMEM and cells were incubated at 37 °C/5% CO_2_.

### Western blot analyses

Cell lysates for Western blotting were prepared using n-dodecyl ß-D-maltoside (DDM) lysis buffer [0.5% DDM (Sigma Aldrich), 0.05% 2 M NaCl, 0.04% 0.5 M Tris-Cl pH 7.5, with 1 cOmplete mini, EDTA-free protease inhibitor cocktail tablet (Roche Diagnostics)] and spun head-over-tail for 1 h at 4 °C to clear the lysates. Lysates were centrifuged at 12,000 rpm for 15 min at 4 °C and cell supernatants were transferred to a new microfuge tube and stored at -70 °C until use. Whole cell lysates were analysed for protein concentration using the Pierce BCA Protein Assay Kit (Thermo Fisher) according to the manufacturer’s instructions. For each sample, 10 µg of whole cell lysate was run out on a sodium dodecyl sulfate-polyacrylamide (SDS-PAGE) gel with a 5% stacking and 15% resolving gel (ProtoGel, National Diagnostics). Proteins were transferred to an Immobilon-FL polyvinylidene fluoride (PVDF) membrane (Millipore) via wet-transfer (Bio-Rad). Membranes were blocked in 5% skim milk powder in Tris-buffered saline with 0.1% Tween 20 (TBS-T) for 1 h with gentle rocking. Membranes were then incubated overnight at 4 °C with primary antibodies in 5% bovine serum albumin (BSA): monoclonal mouse anti-HA (6E2) antibody (Cell Signaling Technologies, 1: 1500), monoclonal rabbit anti-V5 (D3H8Q) antibody (Cell Signaling Technologies, 1: 2000), monoclonal ZIKV rabbit anti-NS2B antibody (Invitrogen, 1:2000), or polyclonal ZIKV rabbit anti-NS3 antibody (GeneTex, 1:1500), polyclonal rabbit Zika anti-capsid antibody (GeneTex, 1:1000), polyclonal rabbit Zika anti-envelope antibody (GeneTex, 1:1000). Blots were washed and incubated with secondary antibodies conjugated to fluorophores diluted in 5% milk for 2 h: goat anti-mouse 520 (Bio-Rad, 1:2500), goat anti-rabbit 700 (Bio-Rad, 1:2500), and/or an anti-GAPDH rhodamine loading control primary antibody (Bio-Rad, 1:2500) as well as 1:5000 SDS. Membranes were washed 6 times with TBS-T for 5 min on a platform shaker before visualizing at the respective wavelengths using the ChemiDoc MP Imaging System (Bio-Rad).

### Densitometry analysis

Densitometric analysis of the ratio of NS2Aα:NS2A was performed in ImageJ [49]. Each band was normalized to the loading control (GAPDH) and is presented as the mean of three independent biological replicates.

### Evolutionary analysis of NS2A across the *Orthoflavivirus* genus

Amino acid sequences of 62 orthoflavivirus NS2A proteins were obtained from NCBI and input into MEGA12 (S1 Table) [50]. The phylogenetic tree was generated using the Neighbor-Joining method and the Jone-Thorton-Taylor matrix-based method was used to determine evolutionary distance [51,52]. Bootstrap resampling was used (100 replicates).

### Conservation analysis of canonical NS3 and the NS2Aα cleavage motifs

The polyprotein sequences of 62 orthoflaviviruses (S1 Table) were aligned using MUSCLE (Snapgene) [53]. NS3-mediated cleavage motif sequences across the polyprotein, including: the internal capsid, NS2A-2B, NS2B-3, NS3-4A, NS4A-2K, NS4B-5 cleavage motifs, were extracted from positions P6-P6´. This amounted to n = 364 sequences, with the exclusion of 8 sequences where the motifs could not be unambiguously identified in the alignment (S1 Table). Similarly, we extracted the NS2Aα cleavage motifs using the ZIKV, YFV, and JEV NS2Aα cleavage motifs as references. This amounted to n = 61 NS2Aα cleavage motif sequences, with the exclusion of 1 sequence where the motif could not be unambiguously identified in the alignment (S1 Table). To generate canonical and NS2Aα cleavage motif sequence logos, we used WebLogo sequence generator to derive the consensus cleavage motifs from positions P6-P6´ [54].

### vRO biogenesis assays

The Zika virus plasmid-induced vRO system (pIRO-Z) encoding the ZIKV (H/PF/2013) strain was provided by the lab of Ralf Bartenschlager [40,41]. This construct encodes for the viral non-structural proteins with the full 3´ UTR and a truncated 5´ UTR lacking the stem-loop A (SLA) and SLB regions. The construct also contains a T7 promoter for RNA synthesis and an encephalomyocarditis virus (EMCV) internal ribosomal entry site (IRES) for translation initiation. The pIRO-Z system was employed as described previously [40,41]. Briefly, Lunet cells, stably expressing cytosolic T7 RNA polymerase (Lunet-T7), were seeded into 24-well plates at 30,000 cells/well. 24 h after seeding, cells were transfected with wild type or mutant pIRO-Z constructs using TransIT transfection reagent (Mirus Bio). 16 hr after transfection, cells were processed for electron microscopy (EM, described below) or immunofluorescence microscopy (IF, described below). IF was used to evaluate transfection efficiency, as determined by the percentage of cells staining positive for the viral NS3 protein. Western blots were used to verify protein expression level. EM quantification of vROs was performed by systematically surveying cells and evaluating for the presence of vROs, followed by imaging at 8000X magnification. Only cells with >4 vROs were considered positive. For each condition, > 40 cells were surveyed over 3 biological replicates. Fiji Software was used for vRO vesicle diameter measurements. Two perpendicular diameters were measure for each vRO, and the average of these values was reported.

### Electron microscopy (EM)

Cells were fixed for 30 min by incubation with pre-warmed 1.25% glutaraldehyde in 0.2 M HEPES. After 3 washes with 0.2 M HEPES, cells were incubated with 2% osmium tetroxide/0.2 M HEPES for 40 min on ice. Cells were washed with water 3 times and treated with 0.5% uranyl acetate for 30 min. After a 30-min wash with water, cells were progressively dehydrated with increasing concentrations of ethanol (40% to 100%), followed by a wash with propylene oxide and the addition of Epon/araldite resin (Araldite 502/Embed 812 kit; Electron Microscopy Sciences), which was then polymerized at 60°C for 72 h. Embedded cells were sectioned into 70-nm-thick slices by using an Ultracut UCT microtome (Leica) and a diamond knife (Diatome). After counterstaining with 3% uranyl acetate in 70% methanol for 5 min and 2% lead citrate in water for 2 min, cells were imaged with a JEOL JEM-1400 120kV LaB6 transmission electron microscope (TEM) with a Gatan US1000 CCD camera (Jeol Ltd., Tokyo, Japan).

### *In vitro* transcription of ZIKV^PR^ full-length and subgenomic replicon RNAs

To generate full-length genomic or subgenomic replicon RNAs, the BAC templates were linearized with *BarI* or *PsrI* restriction enzymes (Creative Enzymes), respectively, and *in vitro* transcribed using the SP6 RNA polymerase. *In vitro* transcription reactions (60 μL) contained 1 mM of each rNTP (New England BioLabs), 0.8 mM m^7^G(5′)ppp(5′)A Cap Structure Analog (New England BioLabs), 1 U/μL RiboLock RNase inhibitor (Thermo Fisher), 50 mM dithiothreitol (Thermo Fisher), 1 U/μL SP6 RNA polymerase (New England BioLabs) and ~250 ng of linearized DNA template. The reactions were incubated for 2 h at 37 °C, followed by treatment with DNaseI (New England BioLabs) for 25 min at 37 °C. Reactions were cleaned up using RNA Clean and Concentrator Kit (Zymo Research) or ethanol precipitation. Firefly luciferase (FLuc) capped electroporation control mRNAs were generated as previously described [55].

### Electroporation of ZIKV^PR^ subgenomic replicon or full-length RNAs

For electroporation, the indicated cells were trypsinized, washed twice in cold PBS, and re-suspended in cold PBS to a concentration of 1 × 10^7^ cells/mL. In a pre-cooled 4-mm cuvette, 400 μL of the cell solution was added to 5 μg of full-length or subgenomic replicon RNA and 1 μg of capped FLuc mRNA. BHK-21 cells were then electroporated with one pulse of 270 volts, 975 capacitance, and infinite resistance on a Bio-Rad Gene Pulser XCell (BioRad). Cells were resuspended in 8 mL of complete media. Two milliliters was placed in a 15 mL conical tube or was plated (2 h and 24 h timepoints), followed by 1 mL into the 48 h well and 500 µL into the 72 h timepoint. Cells were incubated at 37 °C/5% CO_2_ until harvest. For fluorescence microscopy experiments, cells were maintained and electroporated in the same manner as described above before pipetting 5 × 10^4^ (100 μL), 2.5 × 10^4^ (50 μL), or 2 × 10^4^ (40 μL) cells from the 8 mL stock solution into either 96-well microscopy plates (24–72 h timepoints, respectively, Cellvis) or 6-well plates (96 h timepoint) and incubated at 37 °C/5% CO_2_ until time of fix/harvest. For qRT-PCR and ultracentrifuge experiments, Vero cells were electroporated as previously described with the following modifications: 450 V, 25 uF, infinite resistance, 4 mm gap, and 3 pulses, separated by 3 s. In qRT-PCR studies, 6 μg of genomic RNA was electroporated into 400,000 cells and resuspended in four mililiters of DMEM media supplemented with 15 mM HEPES (Wisent, Inc.) and 2% FBS. The entire cell solution was plated into 2 wells of a 6-well dish. Ultra centrifuge experiments required 12 ug of genomic RNA in 800,000 cells, with the entirety plated into two 10-well plates (DMEM media supplemented with 15 mM HEPES (Wisent, Inc.) and 2% FBS). Cells incubated at 37 °C/5% CO_2_ until time of harvest.

### Luciferase assays

For luciferase assays, electroporated cells were washed with PBS and harvested in 150 μL 1X passive lysis buffer (Promega) and stored at -70 °C until use. Luciferase assays were performed using the Dual Luciferase Assay kit (Promega) according to the manufacturer’s instructions, with the exception that 50 μL of each reagent was used and 10 μL of sample. Luminescence output was measured using the GloMax Explorer plate reader (Promega). Luciferase output was analyzed in technical duplicates and data is representative of at least three biological replicates.

### Immunofluorescence assay

Following electroporation of *in vitro* transcribed full-length ZIKV^PR^ genomic RNA into BHK-21 cells, cells were plated into 96-well microscopy plates for the 24–72 h timepoints. Additionally, for subsequent plaque assays, ~ 300,000 electroporated cells were seeded in 6-well plates (96 h timepoint). Cells in microscopy plates were washed twice with 1X Dulbecco’s PBS supplemented with magnesium chloride and calcium chloride (DPBS + MgCl + CaCl, Gibco) followed by a 20 min incubation with 4% paraformaldehyde (PFA) in PBS at room temperature (RT). The PFA solution was replaced with 0.1 M glycine in PBS solution and cells were incubated for a further 10 min at RT. For permeabilization, cells were treated with 0.1% Triton X-100 (in PBS) for 15 min at RT, before 3 washes with DPBS + MgCl + CaCl for 5 min each. Cells were incubated in blocking buffer containing 1% FBS in PBS for 1 h at RT, before addition of the primary antibody, monoclonal mouse anti-HA (Cell Signaling Technologies, 1: 300). Following incubation with the primary antibody for 1 h at RT, the cells were washed 3 times in wash buffer containing 1% FBS and 0.5% Tween-20 in PBS for 5 min each. Subsequently, the cells were incubated with the secondary goat anti-Mouse IgG Alexa Fluor 488 (ThermoFisher, 1:300) for 1 h at RT. Finally, cells were washed 3 times in wash buffer for 5 min each before counterstaining with Antifade Mounting Media containing DAPI (Vector Laboratories) and left to cure overnight. All fluorescence microscopy images were collected on the Nikon Eclipse Ti2 at 20X magnification.

Staining of Lunet-T7 cells transfected with pIRO-Z constructs was performed using the same protocol with the following exceptions: Lunet-T7 cells were grown on 12 mm glass cover slips (Azer Scientific) and transfected for 16 h with pIRO-Z. Primary rat anti-NS3 antibody was used for detection of transfected cells (kindly provided by Dr. Laurent Chatel-Chaix, INRS-Institut Armand-Frappier). Epifluorescence images were obtained using an Axio Observer Z1 microscope with an Axiocam 506 monochromatic camera (Zeiss) or a BioTek Lionheart FX automated widefield microscope (Agilent). Image analysis to determine percent infection, fluorescent signal intensity, and fluorescent signal distribution was performed using CellProfiler [56].

### Plaque forming unit (PFU) assay

Vero cells were plated in a 12-well plate at 2 × 10^5^ cells/well and incubated at 37 °C/5% CO_2_ overnight. The following day, the cell culture media was removed and replaced with 10-fold serial dilutions of viral supernatants. At 2 h post-infection, the viral inoculum was removed, and cells were overlaid with DMEM containing 1.2% carboxymethyl cellulose (Sigma-Aldrich), 2% FBS, and 1% penicillin/streptomycin. Four days post-infection, cells were fixed with 5% formaldehyde and stained with 0.1% crystal violet (Sigma-Aldrich).

### Quantification and statistical analysis

All statistical analyses were preformed using GraphPad Prism, version 10.

## Supporting information

S1 Table Orthoflaviviruses used in phylogenetic analysis. List of orthoflavivirus protein sequences used in phylogenic analysis.
(DOCX)

S2 Table Primer sequences used in molecular cloning. Sequences of primers used in this study.
(DOCX)

S3 Table gBlock sequence used to clone NS2A-2B-3 overexpression vector. Geneblock sequence that was used to create the NS2A-2B-3 expression vector.
(DOCX)

S4 Table RT-qPCR primer and probe sets. List of primer and probe sequences used in RT-qPCR analysis.
(DOCX)

S1 Fig Endogenous tagging of HA-NS2A in full-length ZIKV and the subgenomic replicon RNA does not affect viral kinetics. (A) Representative plaques and average titer of WT and HA-NS2A ZIKV^PR^. (B) Luciferase activity of replication-competent (WT) and replication-defective (GNN) ZIKV^PR^ and HA-NS2A ZIKV^PR^ subgenomic replicon RNAs were electroporated into BHK-21 cells and luciferase activity was monitored at 2–72 h post-electroporation. Error bars represent standard deviation across three independent biological replicates. Lower limit of detection is indicated.
(TIF)

S2 Fig NS2Aα is the predominant isoform of NS2A during ZIKV infection in A549 and C6/36 cells. (A) A549 and (B) C6/36 cells were infected with ZIKV (MOI = 1), total cell lysates were harvested at 8 – 24 h or 24 – 96 h post-infection and analyzed by Western blot. h, hours post-infection.
(TIF)

S3 Fig Ectopically-expressed HA-NS2A and HA-NS2Aα match the molecular weight of NS2A species observed during infection. HA-tagged NS2A, NS2Aα, NS2A-2B-3 expression constructs were introduced into HEK 293T cells and were harvested at 24 h post-transfection. HA-NS2A ZIKV^PR^ (MOI = 1) was used to infect Vero cells and cell lysates were harvested at 18 h post-infection. Cell lysates were analyzed by Western blot.
(TIF)

S4 Fig Polyprotein processing of NS2A mutants in NS2A-2B-3 polyprotein. HA-tagged NS2A-2B-3 expression constructs were introduced into HEK 293T cells and were harvested at 24 h post-transfection. Cell lysates were analyzed by Western blot using HA (NS2A), NS2B, NS3 and GAPDH antibodies. CTD, C-terminal domain.
(TIF)

S5 Fig Transfection efficiency and polyprotein processing of pIRO-Z constructs. (A) Transfection efficiency of pIRO-Z constructs using immunofluorescence staining against rat anti-NS3 antibody. (B) Transfection efficiency/polyprotein processing of NS2A mutants in pIRO-Z system. Antibodies used to detect the different proteins are as follows: NS5, NS1, NS2B, NS3, HA (NS2A), and GAPDH.
(TIF)

S6 Fig Mutations that impair NS2Aα biogenesis impair genome packaging. (A) Percent of ZIKV genomic RNA in the supernatant relative to WT via RT-qPCR analyses. (B) Western blot analyses of capsid, envelope, and NS2A proteins in supernatant and cell lysates, respectively.
(TIF)

S1 Raw Gel Images Uncropped and annotated Western blots corresponding to the figures presented in the manuscript. Uncropped and annotated Western blot images used to format the main and supplementary figures. The corresponding main/supplementary blots are indicated on the raw images. Molecular weight markers are indicated.
(PDF)

S1 File Supplemental Methods_21Sept2026. (DOCX)
