# Supplementary material for: Zika virus produces two isoforms of the NS2A protein that play mutually exclusive roles in viral replication and virion assembly
Source: PLoS Pathog. 2026 Oct 12;22(10):e1014609. doi: 10.1371/journal.ppat.1014609 (PMC13671652; doi:10.1371/journal.ppat.1014609)
Supplement: Supplementary file 1 [file ppat.1014609.s011.pdf]

Supporting Information – Raw Western Blot Images

Contained in this file are the raw, annotated, western blot images for Landry et al. 2026 (PPATHOGENS-D-26-00062R1) both for the main figure and supplemental:

Main Figures

Western blot images were generated using fluorescence imaging with ChemiDoc MP Imaging System (Bio-Rad). Respective fluorophores/wavelengths were used to generate images: goat anti-mouse 520 (Bio-Rad, 1:2500), goat anti-rabbit 700 (Bio-Rad, 1:2500), and/or an anti-GAPDH rhodamine loading control primary antibody (Bio-Rad, 1:2500). Imaging was exposed according to machine preset of "Optimal Auto-exposure".

Figure 1B (page 1)

Figure 2C, D, and E (page 2-4)

Figure 4A, B and C (page 5-7)

Supporting Information Figures

Unless stated otherwise, Western blot images were generated using fluorescence imaging with ChemiDoc MP Imaging System (Bio-Rad). Respective fluorophores/wavelengths were used to generate images: goat anti-mouse 520 (Bio-Rad, 1:2500), goat anti-rabbit 700 (Bio-Rad, 1:2500), and/or an anti-GAPDH rhodamine loading control primary antibody (Bio-Rad, 1:2500). Imaging was exposed according to machine preset of "Optimal Auto-exposure".

Figure S2A and B (pages 8-9)

Figure S3 (page 10)

Figure S4 (page 11)

Figure S5B (page 12-13)

Blot image was generated using a BioRad ChemiDoc. Secondaries include Goat anti-Rabbit Peroxidase Conjugate (A6154-1ML from Sigma Aldrich), Goat anti-Rat Peroxidase Conjugate (SAB3700541-2MG from Millipore), and Goat anti-Mouse Peroxidase Conjugate (A4416-1ML from Sigma Aldrich) diluted 1:5000. Membranes were incubated with Cytiva Amersham (RPN2235) for 5 minutes, and then imaged.

Figure S6B(1)(2) (page 12-13)

Fig1B

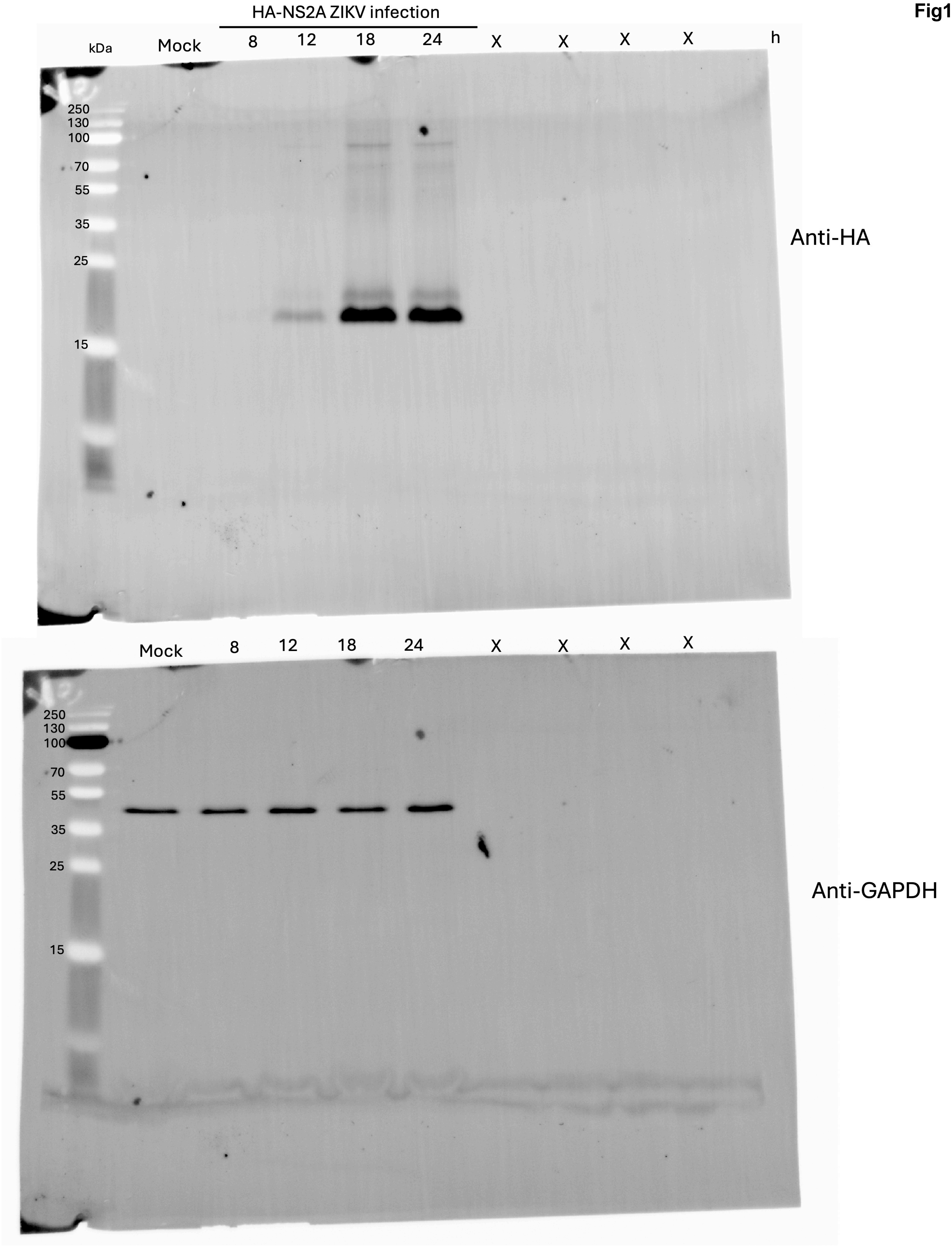
HA-NS2A ZIKV infection
kDa
Mock
8
12
18
24
X
X
X
X
h
250
130
100
70
55
35
25
15
Anti-HA
Mock
8
12
18
24
X
X
X
X
250
130
100
70
55
35
25
15
Anti-GAPDH

Fig2C

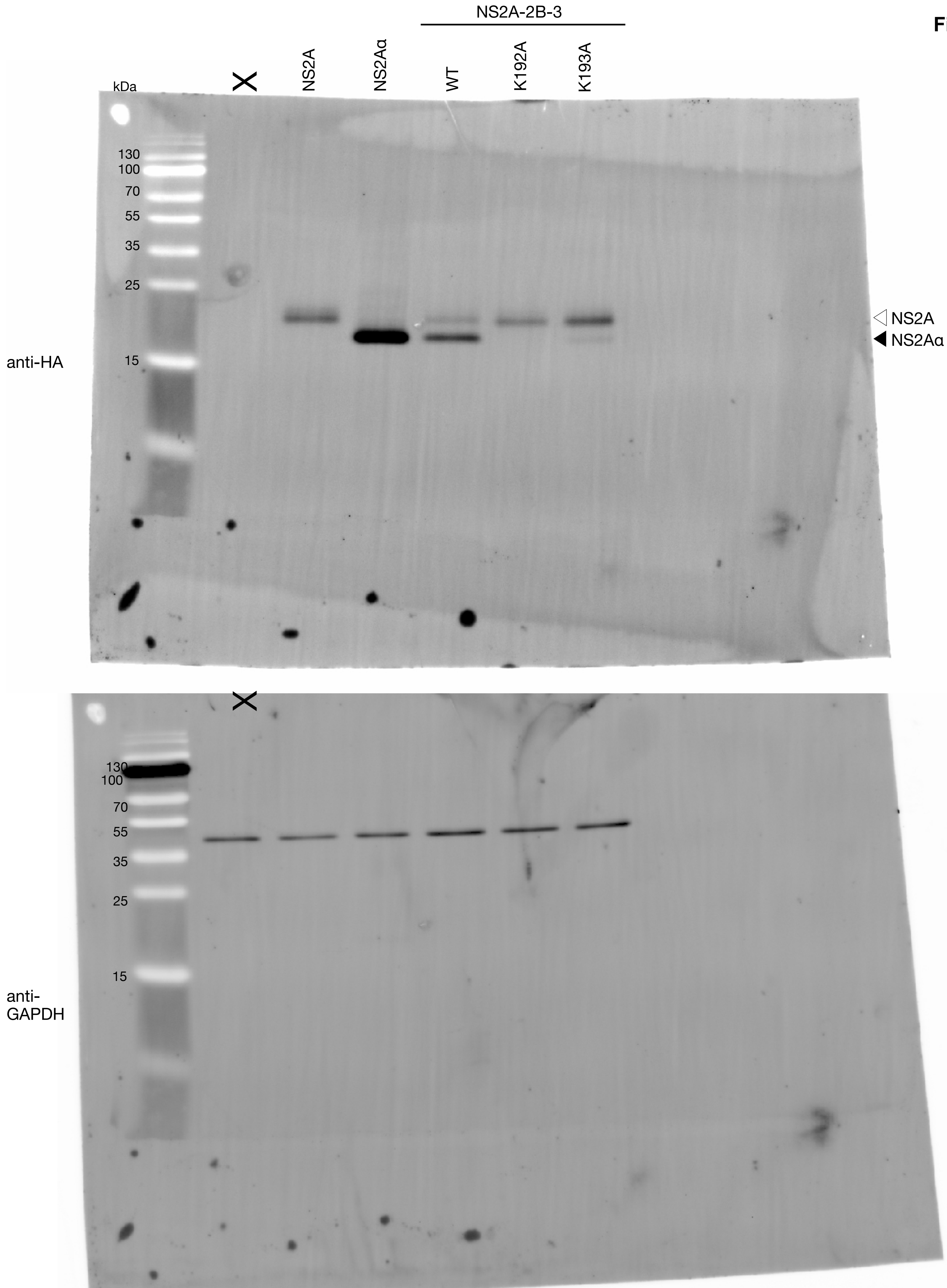
NS2A-2B-3
NS2A
NS2Aα
WT
K192A
K193A
kDa
130
100
70
55
35
25
15
anti-HA
◁ NS2A
◀ NS2Aα
130
100
70
55
35
25
15
anti-
GAPDH

Fig2D

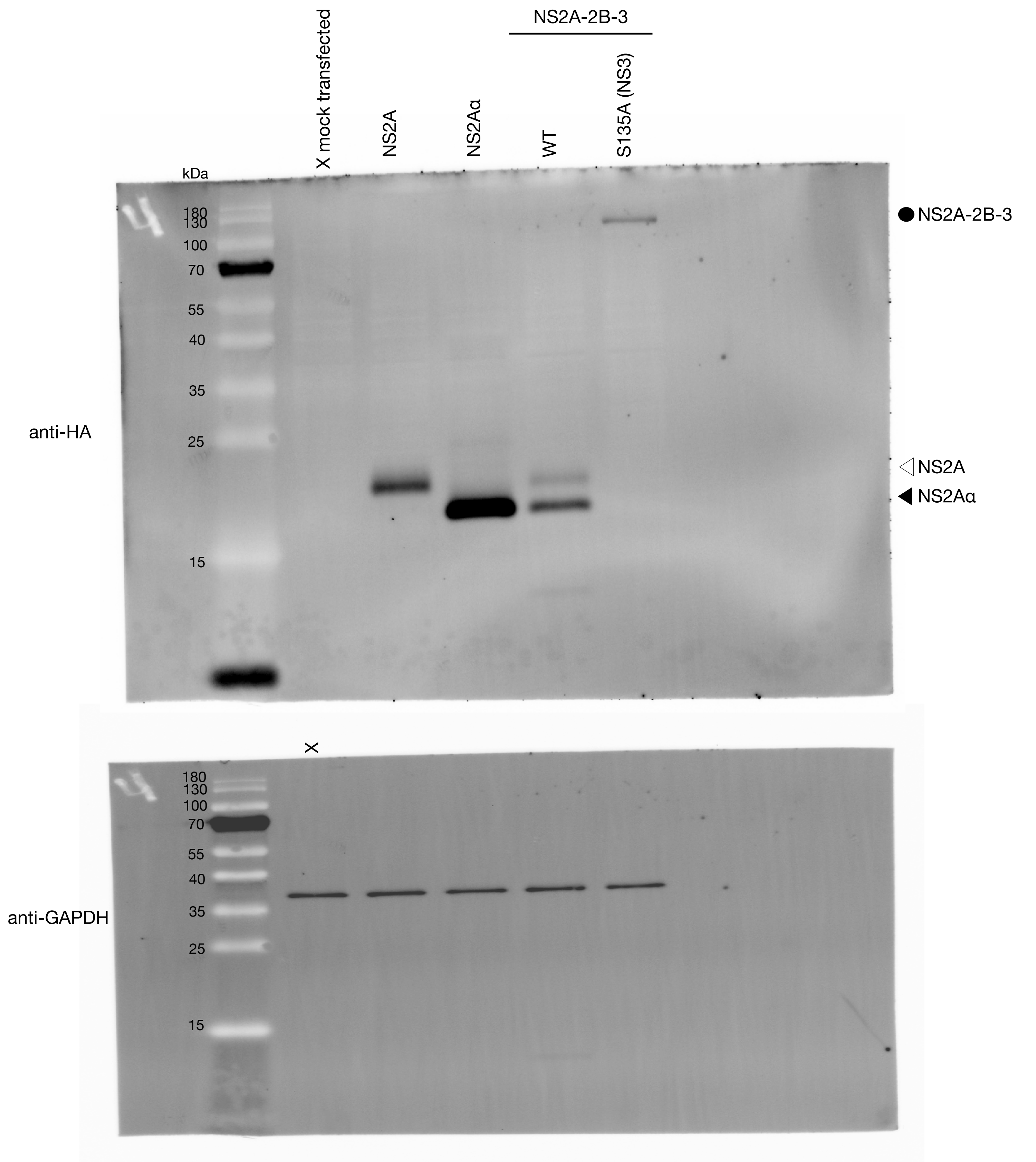
NS2A-2B-3
X mock transfected
NS2A
NS2Aα
WT
S135A (NS3)
kDa
180
130
100
70
55
40
35
25
15
anti-HA
NS2A-2B-3
NS2A
NS2Aα
X
180
130
100
70
55
40
35
25
15
anti-GAPDH

Fig2E

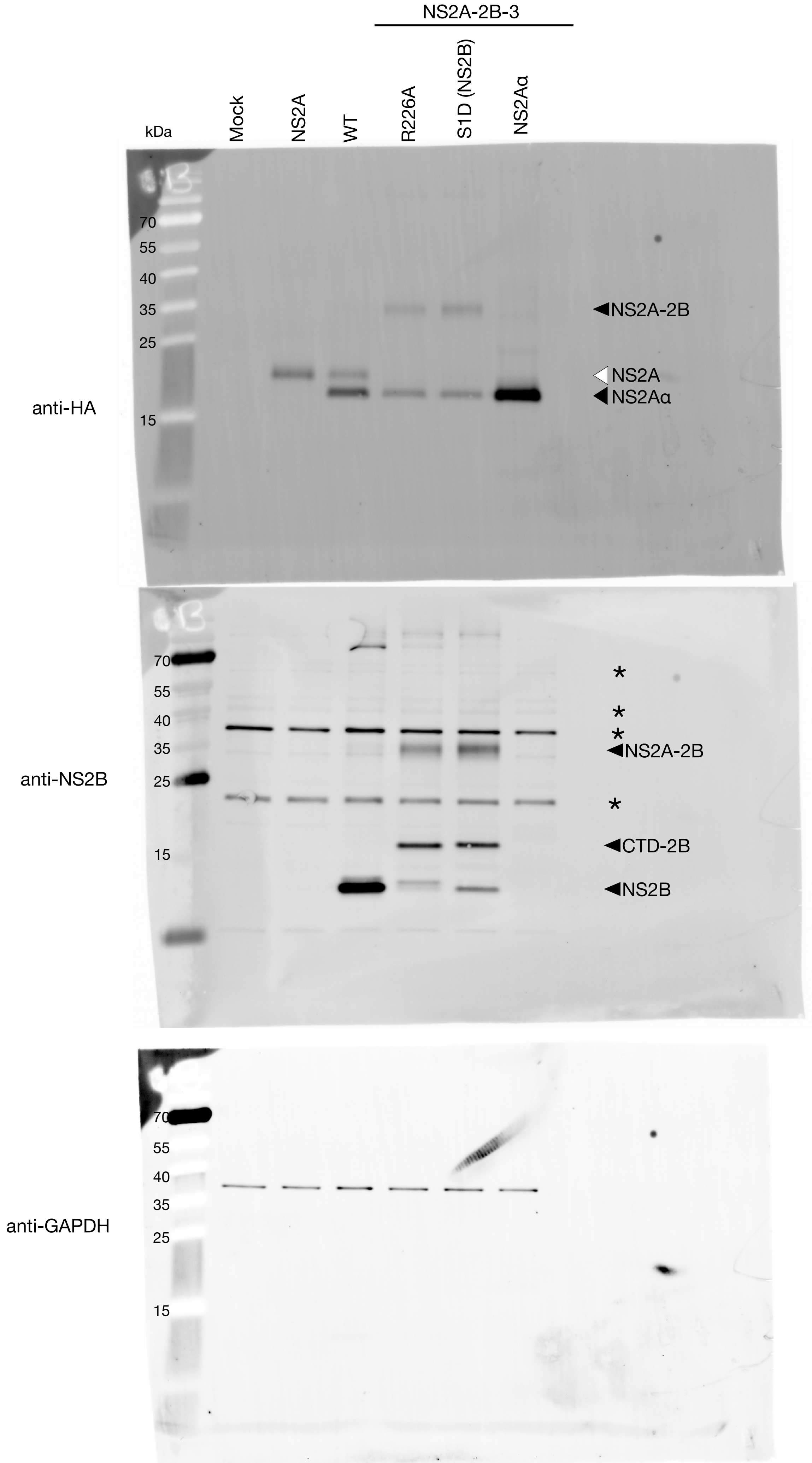
NS2A-2B-3
kDa
Mock
NS2A
WT
R226A
S1D (NS2B)
NS2Aα
70
55
40
35
25
15
NS2A-2B
NS2A
NS2Aα
anti-HA
*
*
*
NS2A-2B
*
CTD-2B
NS2B
anti-NS2B
anti-GAPDH

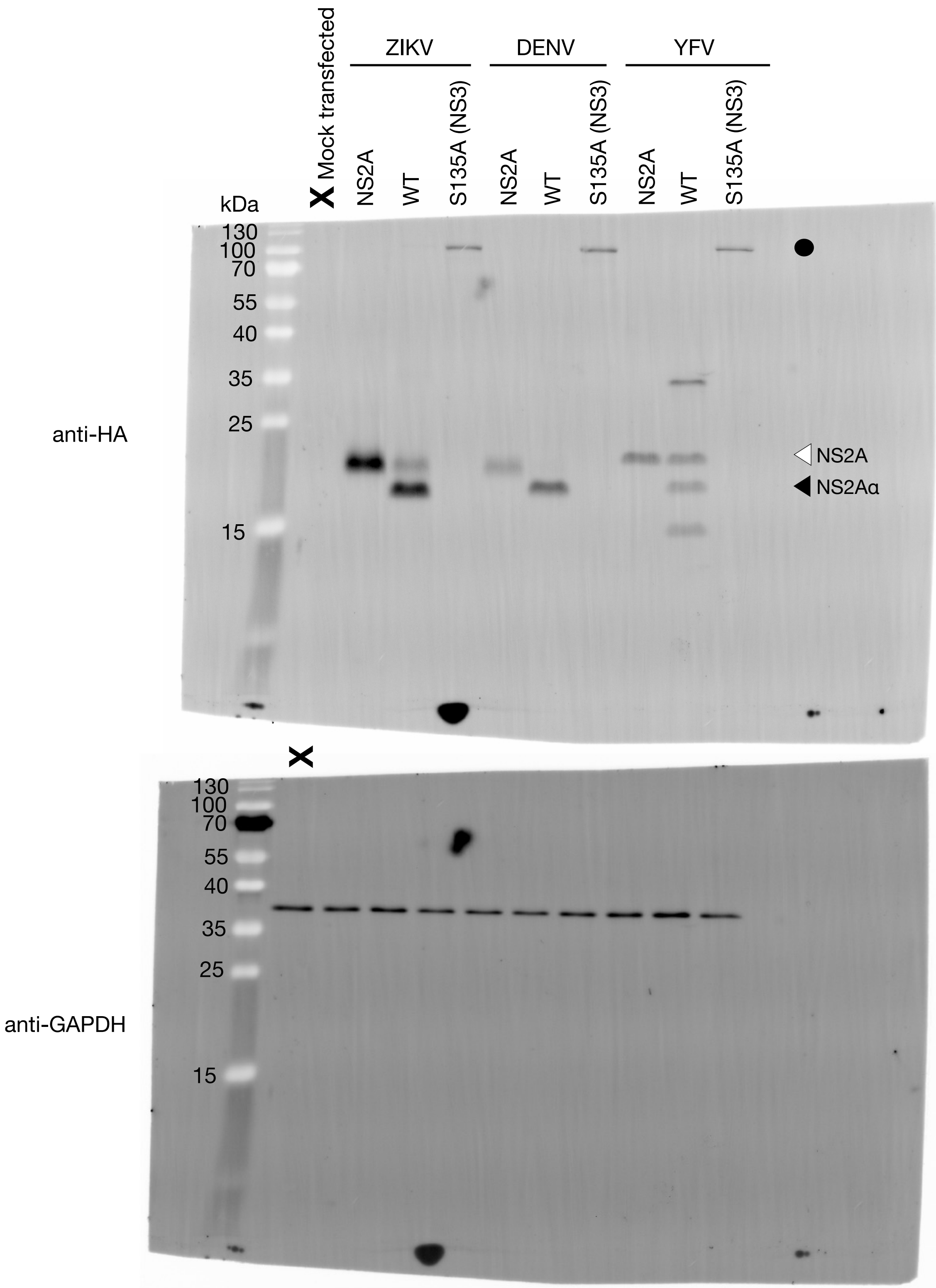
Mock transfected
ZIKV
DENV
YFV
NS2A
WT
S135A (NS3)
NS2A
WT
S135A (NS3)
NS2A
WT
S135A (NS3)
kDa
130
100
70
55
40
35
25
15
anti-HA
NS2A
NS2Aα
130
100
70
55
40
35
25
15
anti-GAPDH

Fig4B

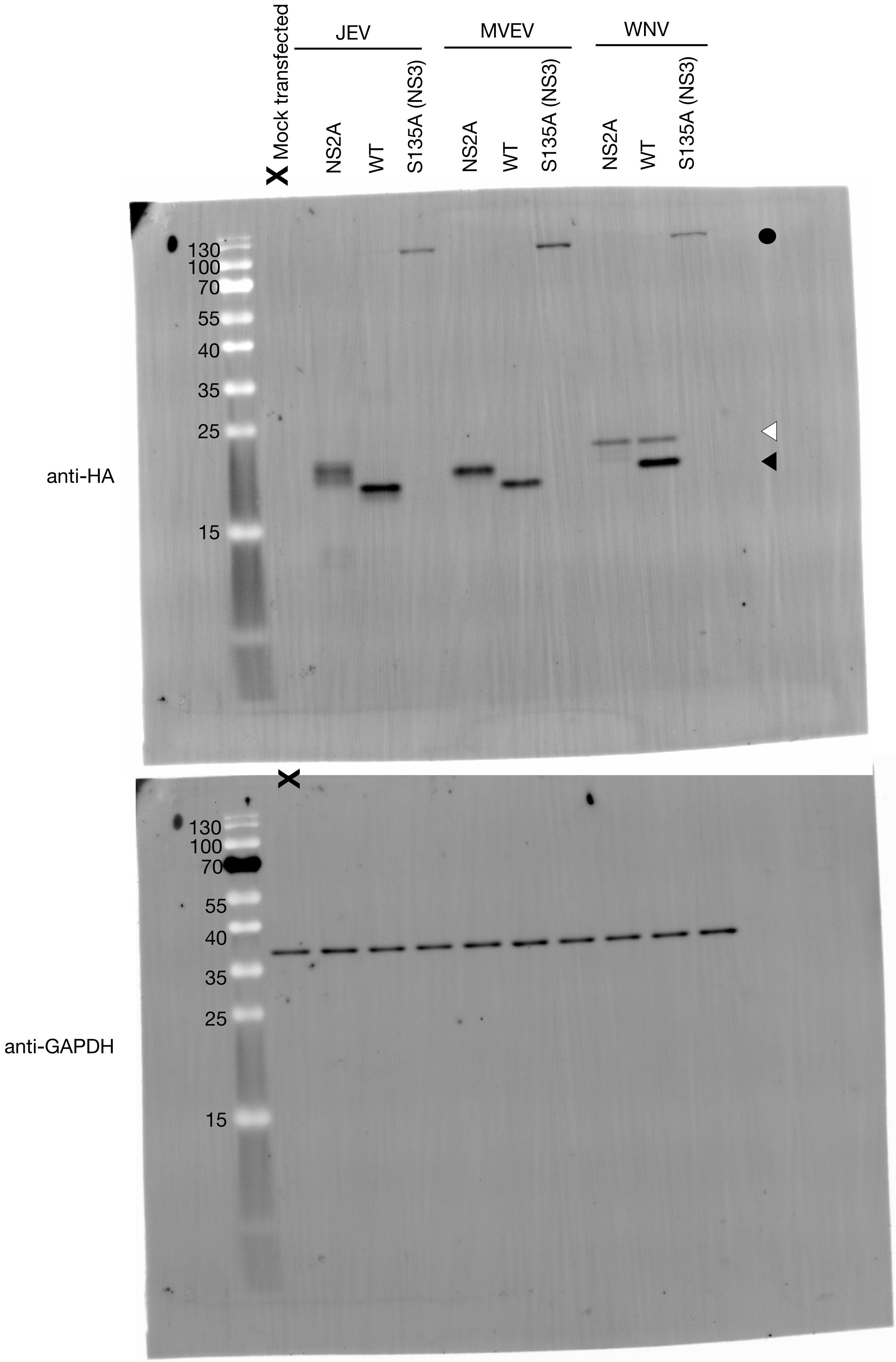
Mock transfected
JEV
MVEV
WNV
NS2A
WT
S135A (NS3)
NS2A
WT
S135A (NS3)
NS2A
WT
S135A (NS3)
130
100
70
55
40
35
25
15
anti-HA
130
100
70
55
40
35
25
15
anti-GAPDH

Fig4C

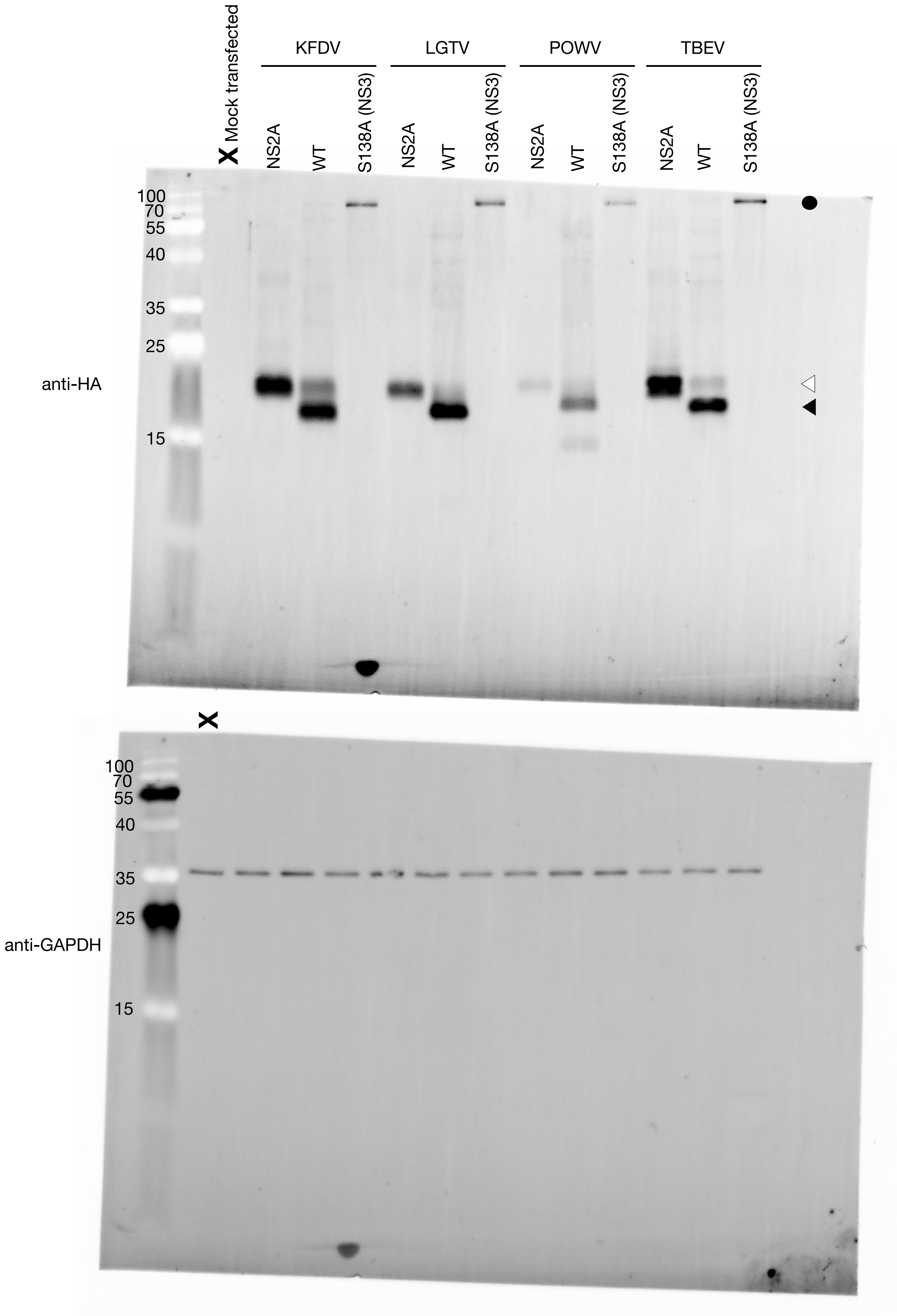

Mock transfected
KFDV
LGTV
POWV
TBEV
NS2A
WT
S138A (NS3)
100
70
55
40
35
25
15
anti-HA
anti-GAPDH

FigS2A

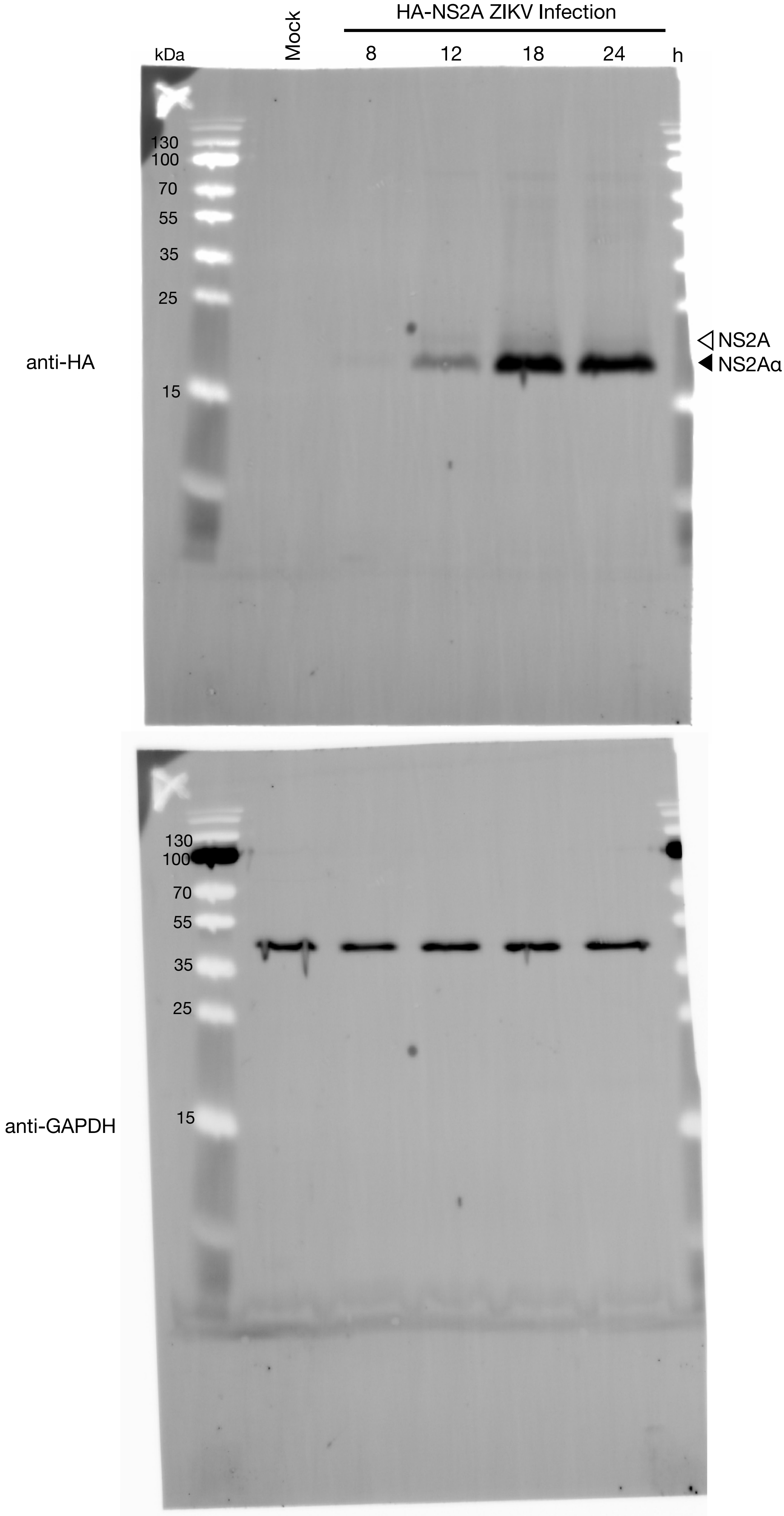

HA-NS2A ZIKV Infection
Mock
8
12
18
24
h
kDa
130
100
70
55
35
25
15
anti-HA
NS2A
NS2Aα
anti-GAPDH

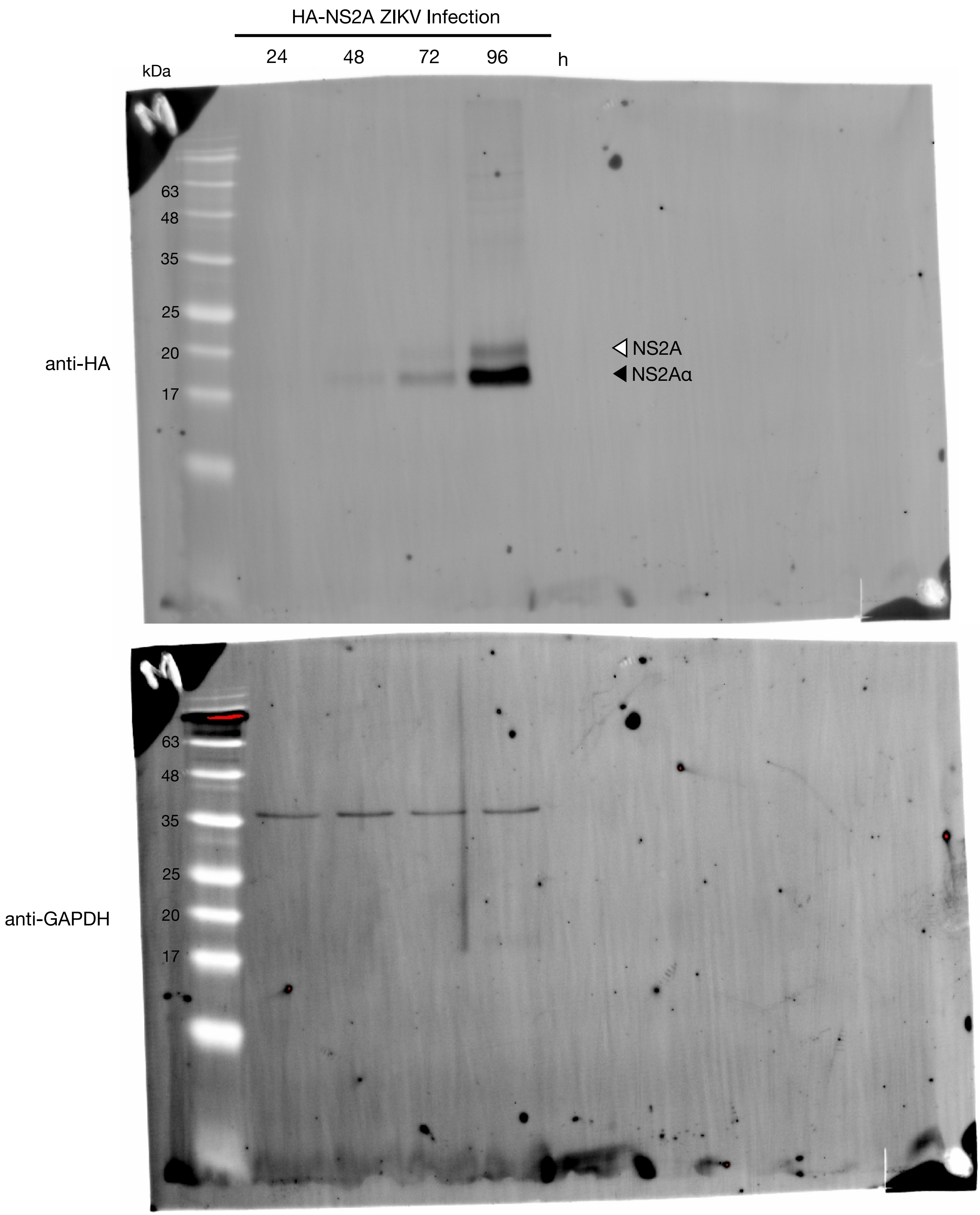
HA-NS2A ZIKV Infection
24
48
72
96
h
kDa
63
48
35
25
20
17
anti-HA
NS2A
NS2Aα
63
48
35
25
20
17
anti-GAPDH

FigS3

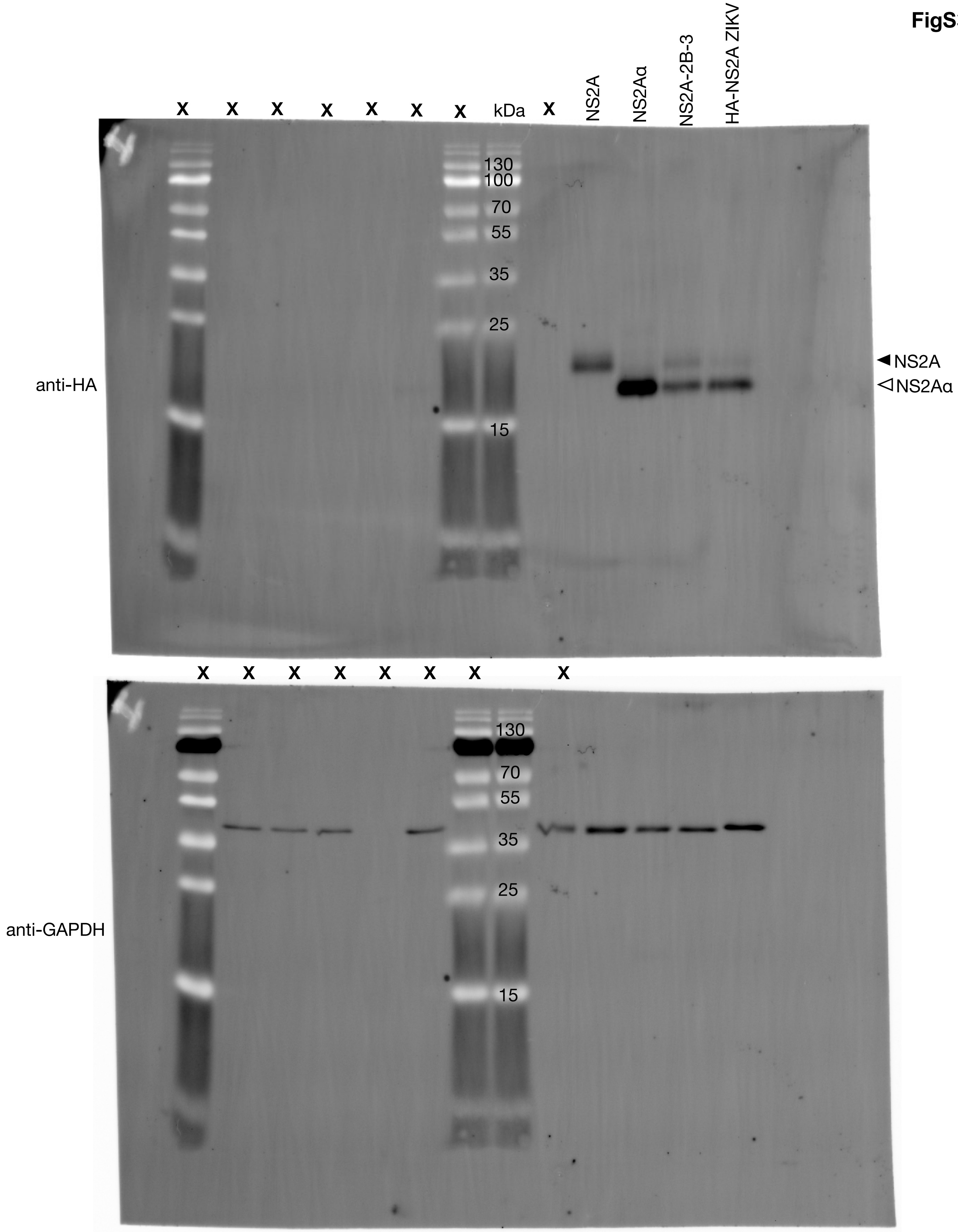
x x x x x x x kDa x
NS2A
NS2Aα
NS2A-2B-3
HA-NS2A ZIKV
130
100
70
55
35
25
15
anti-HA
NS2A
NS2Aα
x x x x x x x x
130
70
55
35
25
15
anti-GAPDH

FigS4

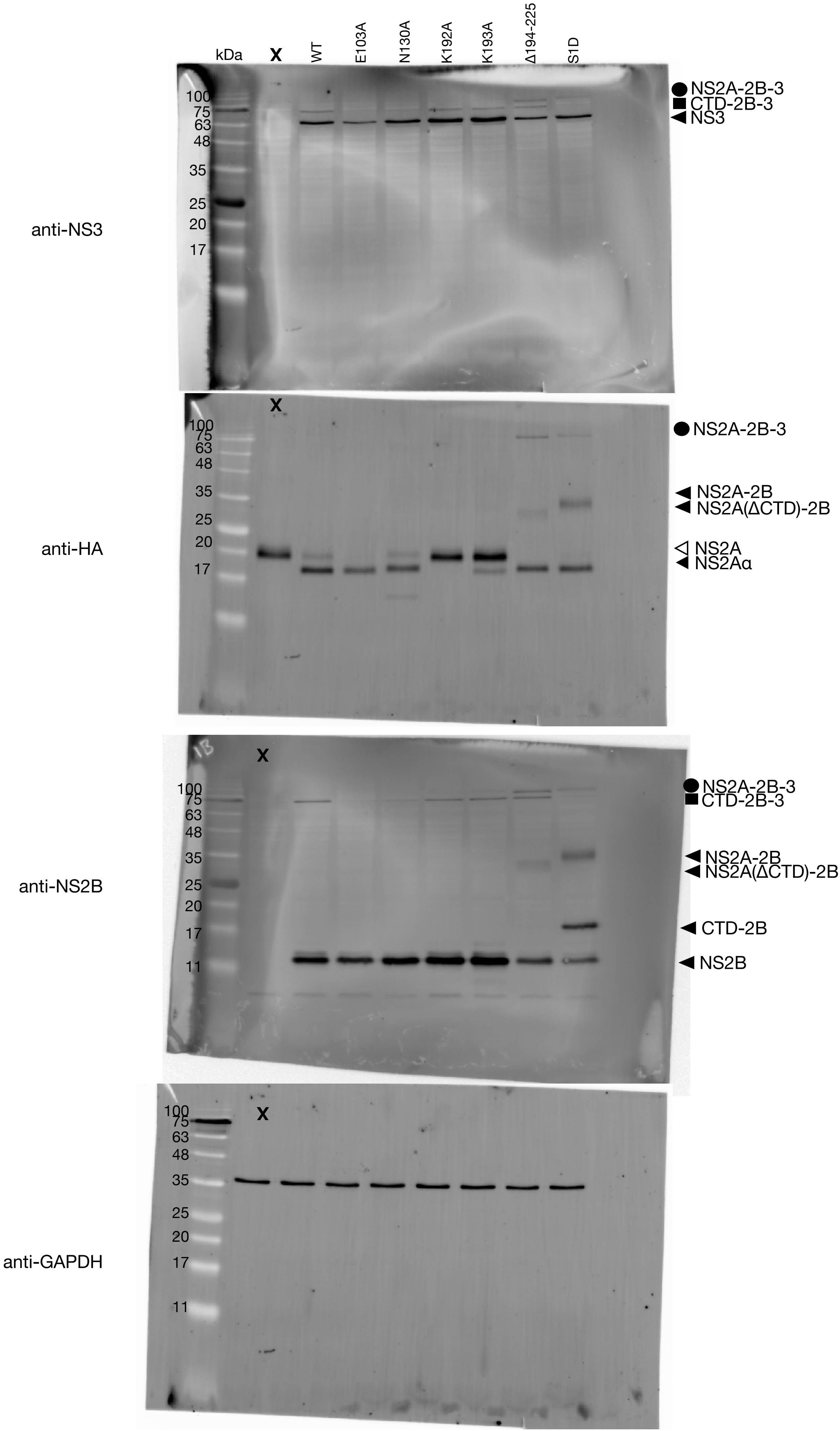

kDa
X
WT
E103A
N130A
K192A
K193A
Δ194-225
S1D
anti-NS3
100
75
63
48
35
25
20
17
NS2A-2B-3
CTD-2B-3
NS3
anti-HA
X
100
75
63
48
35
25
20
17
NS2A-2B-3
NS2A-2B
NS2A(ΔCTD)-2B
NS2A
NS2Aα
anti-NS2B
X
100
75
63
48
35
25
20
17
11
NS2A-2B-3
CTD-2B-3
NS2A-2B
NS2A(ΔCTD)-2B
CTD-2B
NS2B
anti-GAPDH
X
100
75
63
48
35
25
20
17
11

FigS5B

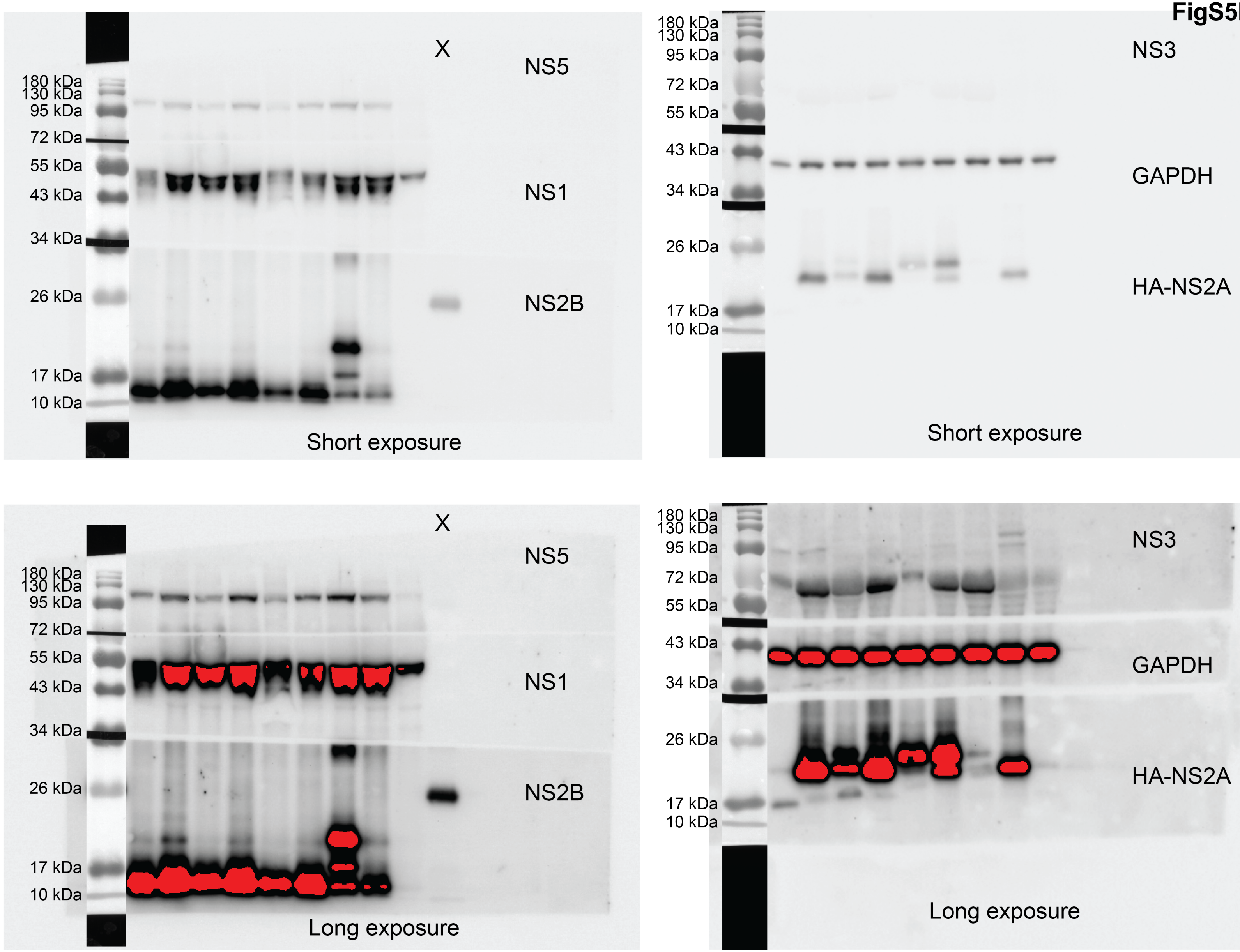
180 kDa
130 kDa
95 kDa
72 kDa
55 kDa
43 kDa
34 kDa
26 kDa
17 kDa
10 kDa
X
NS5
NS1
NS2B
Short exposure
NS3
GAPDH
HA-NS2A
Short exposure
Long exposure
Long exposure

Fig6B(1)

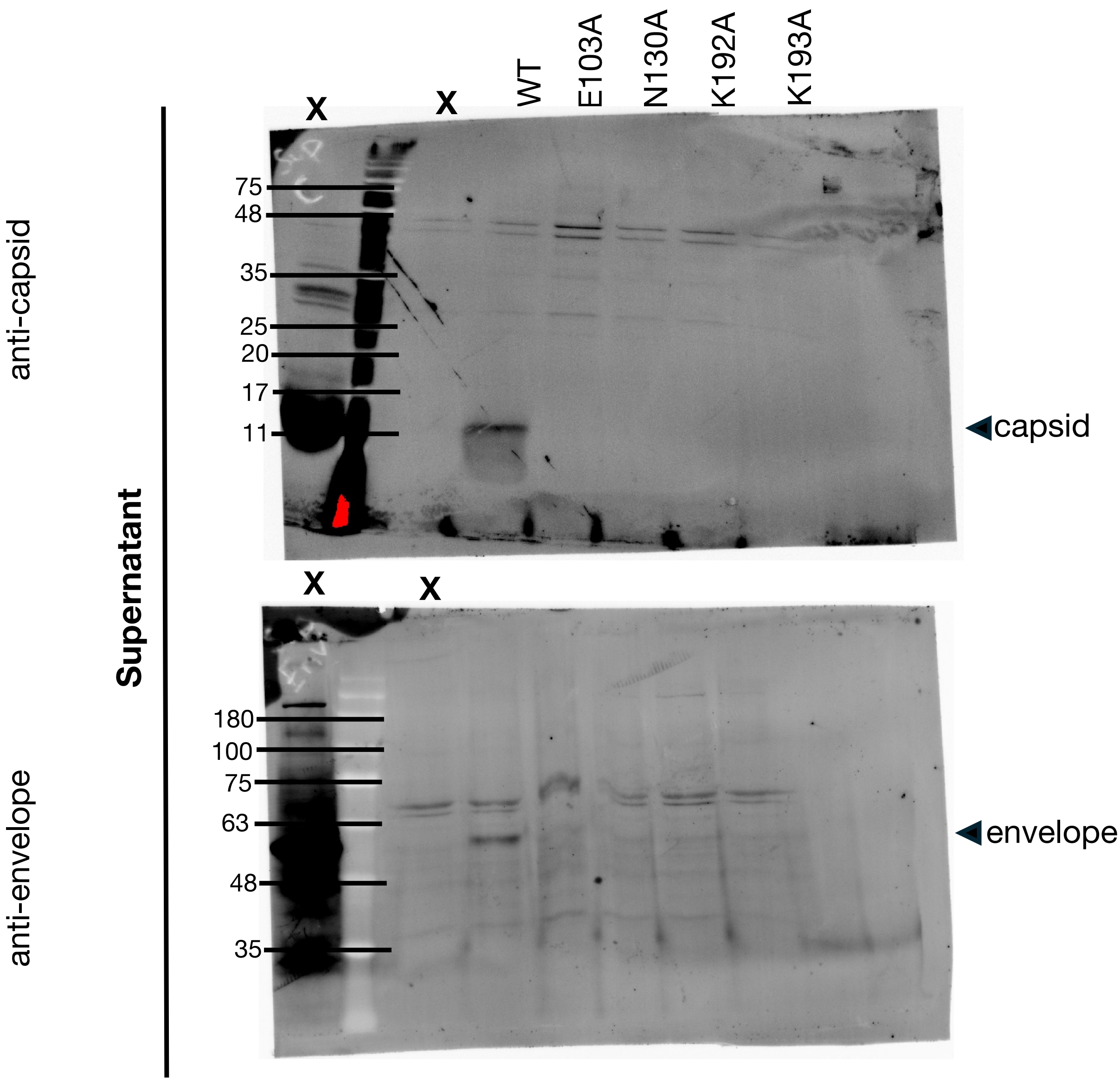
WT
E103A
N130A
K192A
K193A
X
X
anti-capsid
75
48
35
25
20
17
11
capsid
Supernatant
anti-envelope
180
100
75
63
48
35
envelope

Fig6B(2)

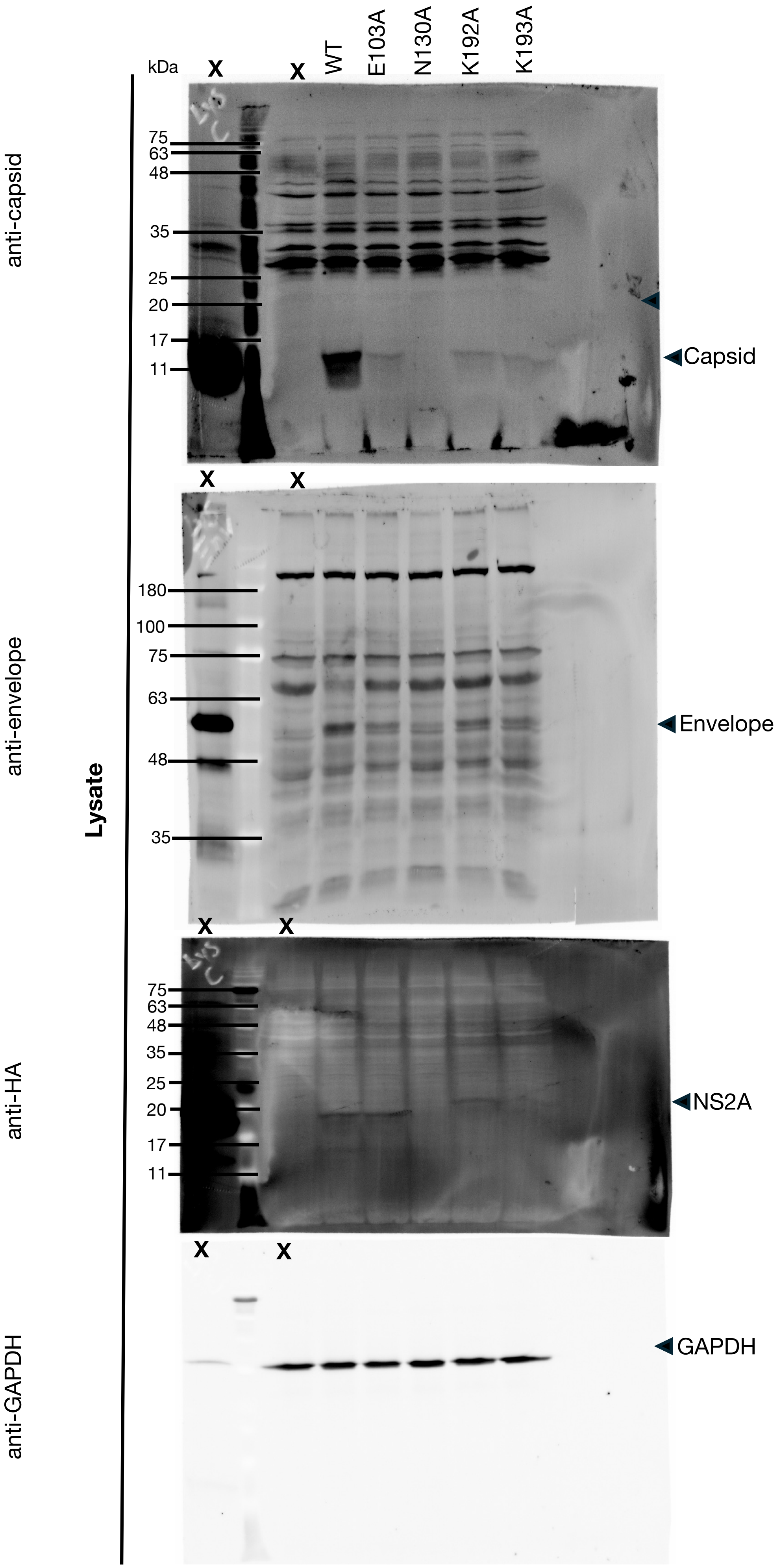

kDa
X
X
WT
E103A
N130A
K192A
K193A
anti-capsid
75
63
48
35
25
20
17
11
Capsid
X
X
anti-envelope
180
100
75
63
48
35
Envelope
Lysate
X
X
anti-HA
75
63
48
35
25
20
17
11
NS2A
X
X
anti-GAPDH
GAPDH
