# Supplementary material for: Zika virus produces two isoforms of the NS2A protein that play mutually exclusive roles in viral replication and virion assembly
Source: PLoS Pathog. 2026 Oct 12;22(10):e1014609. doi: 10.1371/journal.ppat.1014609 (PMC13671652; doi:10.1371/journal.ppat.1014609)
Supplement: Supplementary file 2 [file ppat.1014609.s001.docx]

**Table S1. Orthoflaviviruses used in phylogenetic analysis.**

| **Name** | **Genbank #** | **Name** | **Genbank #** |
| --- | --- | --- | --- |
| Alfuy virus (ALFV) | AY898809.1 | Louping ill virus (LOUV) | NC_001809.1 |
| Alkhumra hemorrhagic fever virus (AHFV) | JF416957.1 | Meaban virus (MEAV) | NC_033721.1 |
| Apoi virus (APOIV) | NC_003676.1 | Modoc virus (MODV)^a^ | NC_003635.1 |
| Aroa virus (AROAV) | YP_001040004.1 | Montana myotis leuko-encephalitis virus (MMLV) | NC_004119.1 |
| Bagaza virus (BAGV) | NC_012534.1 | Murray Valley encephalitis virus (MVEV) | KX229766.1 |
| Banzi virus (BANV) | NC_043110.1 | Naranjal virus (NJLV) | KF917538.1 |
| Barkedji virus (BJV) | LC497470.1 | Nounane virus (NOUV) | NC_033715.1 |
| Batu cave virus (BCV) | KJ469370.1 | Ntaya virus (NTAV) | NC_018705.3 |
| Bouboui virus (BOUV)^a^ | NC_033693.1 | Omsk hemorrhagic fever virus (OHFV) | NC_005062.1 |
| Chaoyang virus (CYV) | NC_017086.1 | Phnom Penh virus (PPBV) | NC_034007.1 |
| Deer tick virus (DTV) | AF311056.1 | Potiskum virus (POTV) | NC_029054.2 |
| Dengue virus 1 (DENV-1) | NC_001477.1 | Powassan virus (POWV) | OP037823.1 |
| Dengue virus 2 (DENV-2) | KU725663.1 | Rio Bravo virus (RBV) | NC_003675.1 |
| Dengue virus 3 (DENV-3) | NC_001475.2 | Rocio virus (ROCV) | NC_040776.1 |
| Dengue virus 4 (DENV-4) | NC_002640.1 | Royal farm virus (RFV) | NC_039219.1 |
| Edge Hill virus (EHV)^a^ | NC_030289.1 | Saboya virus (SABV) | NC_033697.1 |
| Entebbe bat virus (ENTV)^a^ | NC_008718.1 | Sepik virus (SEPV)^a^ | NC_008719.1 |
| Gadgets Gully virus (GGYV) | NC_033723.1 | Sokoluk virus (SOKV)^a^ | NC_026624.1 |
| Iguape virus (IGUV) | AY632538.4 | Spondweni virus (SPOV) | MG182017.2 |
| Ilheus virus (ILHV) | NC_009028.2 | St. Louis encephalitis virus (SLEV) | NC_007580.2 |
| Japanese encephalitis virus (JEV) | AY585243.1 | Tembusu virus (TMUV) | NC_015843.2 |
| Jugra virus (JUGV) | NC_033699.1 | Tick-borne encephalitis virus (TBEV) | JF819648.2 |
| Jutiapa virus (JUTV) | NC_026620.1 | Tyuleniy virus (TYUV) | NC_023424.1 |
| Kadam virus (KADV) | NC_033724.1 | Uganda S virus (UGSV) | NC_033698.1 |
| Kedougou virus (KEDV) | NC_012533.1 | Usutu virus (USUV) | NC_006551.1 |
| Kokobera virus (KOKV | NC_009029.2 | West Nile virus (WNV) | KC407666.1 |
| Koutango virus (KOUV) | PP885725.1 | Wesselsbron virus (WSLV)^a^ | NC_012735.1 |
| Kunjin virus (KUNV) | KX394389.1 | Yellow fever virus (YFV) | MN106241.1 |
| Kyasanur forest disease virus (KFDV) | JF416958.1 | Yaounde virus (YAOV) | NC_034018.1 |
| Lammi virus (LAMV) | NC_024806.1 | Yokose virus (YOKV)^a^ | NC_005039.1 |
| Langat virus (LGTV) | AAF75260.1 | Zika virus (ZIKV) | KU501215.1 |

^a^One or more NS3 cleavage motif(s) could not be unambiguously identified in the alignment.
