# Supplementary material for: Zika virus produces two isoforms of the NS2A protein that play mutually exclusive roles in viral replication and virion assembly
Source: PLoS Pathog. 2026 Oct 12;22(10):e1014609. doi: 10.1371/journal.ppat.1014609 (PMC13671652; doi:10.1371/journal.ppat.1014609)
Supplement: Supplementary file 3 [file ppat.1014609.s002.docx]

**Table S2. Primer sequences used in cloning**

| **Primer Name** | **Sequence (5’-3’)** |
| --- | --- |
| ZIKV-HA-NS2A FOR | TAC CCA TAC GAC GTG CCA GAC TAC GCC GGC GGC GGC GGA TCA ACT GAT CAC ATG GAC |
| ZIKV-HA-NS2A-REV | GGC GTA GTC TGG CAC GTC GTA TGG GTA GCC GCT GCC TGC AGT CAC CAT TGA CCT TAC |
| E103A-NS2A-FWD | ACA CCC CGT GCT AGC ATG CTG C |
| E103A-NS2A-REV | CCA ATT AGC TCT GAA GAT GAA AGA TAC |
| N130A-NS2A-FWD | GGT TCT CAT CGC TGG TTT TGC TTT GGC |
| N130A-NS2A-REV | ATC AGG TCG CCT TCC AAG |
| K192A-NS2A-FWD | AGG CAG TGT GGC GAA GAA CTT AC |
| K192A-NS2A-REV | TTT CCC TTC AGA GAG AGG |
| K193A-NS2A-FWD | CAG TGT GAA GGC GAA CTT ACC ATT TG |
| K193A-NS2A-REV | CCT TTT CCC TTC AGA GAG |
| S1D-NS2B-FWD | TGG GAA GCG GGA CTG GCC CCC TA |
| S1D-NS2B-FWD | CCT TTT CCC TTC AGA GAG |
| BAC-GA-FWD | ATG GTG CTG CAG GGA GTG CAC AAT GCC CCC ACT G |
| BAC-GA-REV | GTC AGC TTT AAA GTT GGC GCC CAT CTC TGA AAT GTC |
| NheI-MreI-NS2A-FOR | ATA TGC TAG CAG ACT ACG CCG GCG GCG GCG GAT CAA CTG ATC ACA TGG ACC |
| NS3-stop-XhoI-Rev | ACT ACC TCG AGT TAT CTT TTC CCA GCG GCA AAC TC |
| Δ2ApeptideFMDV-FWD | GGC AGC GGC TAC CCA TAC G |
| Δ2ApeptideFMDV-REV | GGC CTC GGC CAC GGC GAT |
| Δ194-225-NS2A-FWD | CGG AGC TGG CCC CCT |
| Δ194-225-NS2A-REV | CTT CTT CAC ACT GCC TTT TCC CTT CAG AGA G |
| S135A-NS3-FWD | AGC AGG AAC TGC AGG ATC TCC |
| S135A-NS3-REV | GGG TAA TCC AGC GCA ACC |
| Δ2B-3-FWD | TAACTCGAGTCTAGAGGGC |
| Δ2B-3-REV | CCGCTTCCCACTCCTTGT |
| Lin-HA-NS2ApcDNA-FWD | TAA CTC GAG TCT AGA GGG CCC GTT TAA ACC C |
| Lin-HA-NS2ApcDNA-REV | GCC GCC GCC GGC GTA GTC |
| DENV-2-Gibamp-FWD | TAC GCC GGC GGC GGC GGA CAT GGG CAG GTA GAC AA |
| DENV-2-Gibamp-REV | TCT AGA CTC GAG TTA CTT TCT TCC GGC TGC AAA TTC C |
| YFV-Gibamp-FWD | TAC GCC GGC GGC GGC GGA GAA ATA CAT GCT GTC CC |
| YFV-Gibamp-REV | TCT AGA CTC GAG TTA CCT CCT ACC TTC AGC AAA CT |
| JEV-Gibamp-FWD | TAC GCC GGC GGC GGC TTC AAT GGT GAA ATG GTT GAC C |
| JEV-Gibamp-REV | TCT AGA CTC GAG TTA TCT CTT TCC TGC TGC AAA GTC |
| MVEV-Gibamp-FWD | TAC GCC GGC GGC GGC TTT AAT GGA GAT ATG ATT GAT CCT TTT CAG TTA G |
| MVEV-Gibamp-REV | TCT AGA CTC GAG TTA CCG CTT CCC AGC TGC AAA G |
| WNV-Gibamp-FWD | TAC GCC GGC GGC GGC TAT AAT GCT GAT ATG ATT GAC CCT TTT CAG |
| WNV-Gibamp-REV | TCT AGA CTC GAG TTA ACG TTT TCC CGA GGC GAA G |
| KFDV-Gibamp-FWD | TAC GCC GGC GGC GGC GAC AAC GGA GCC ATG CTA AGT GA |
| KFDV-Gibamp-REV | TCT AGA CTC GAG TTA GCG ACG CCC GGA GG |
| LGTV-Gibamp-FWD | TAC GCC GGC GGC GGC GAC AAC GGG GCT CTA CTC AG |
| LGTV-Gibamp-REV | TCT AGA CTC GAG TTA GCG TCT TCC AGA AGC ATA GG |
| POWV-Gibamp-FWD | TAC GCC GGC GGC GGC GAC AAT GGT GCC TTG CTT AG |
| POWV-Gibamp-REV | TCT AGA CTC GAG TTA CCT ACG ACC GGA AGC GTAC |
| TBEV-Gibamp-FWD | TAC GCC GGC GGC GGC GAC AAT GGA GAG CTG CTC AG |
| TBEV-Gibamp-REV | TCT AGA CTC GAG TTA GCG TCT CCC TGA GGC ATA C |
| DENV-NS2A-B-3-S135A-FWD | TCC TGG AAC GGC CGG ATC TCC AA |
| DENV-NS2A-B-3-S135A-REV | GAA AAG TCC AGA GAT ACA GC |
| YFV-NS2A-B-3-S138A-FWD | GAG TGG CAC TGC CGG ATC TCC TAT TG |
| YFV-NS2A-B-3-S138A-REV | GGA TAG TCA AGA GCG ACA G |
| JEV-NS2A-B-3-S135A-FWD | GCG AGG AAC AGC AGG CTC ACC CA |
| JEV-NS2A-B-3-S135A-REV | GGG TAA TCC AGG CTA ACA GC |
| MVEV-NS2A-B-3-S135A-FWD | GAT TGG AAC TGC CGG ATC CCC AAT AG |
| MVEV-NS2A-B-3-S135A-REV | GGG TAA TCC AAG CTC ACG |
| WNV-NS2A-B-3-S135A-FWD | CAC TGG AAC AGC CGG CTC ACC AA |
| WNV-NS2A-B-3-S135A-REV | GGG AAG TCC AAA GTC ACG |
| KFDV-NS2A-B-3-S138A-FWD | CAA AGG CAC CGC CGG AAG TCC TAT C |
| KFDV-NS2A-B-3-S138A-REV | CTC AGG TCA ATA GGG ACT G |
| LGTV-NS2A-B-3-S138A-FWD | CAA AGG AAC AGC CGG CAG TCC AA |
| LGTV-NS2A-B-3-S138A-REV | GCG AGA TCA ATG GGA ATG |
| POWV-NS2A-B-3-S138A-FWD | ACG TGG TAC TGC CGG TAG CCC AA |
| POWV-NS2A-B-3-S138A-REV | GGG AGG TCT ATC GGG ATG |
| TBEV-NS2A-B-3-S138A-FWD | AAA AGG GAC AGC CGG CAG TCC CA |
| TBEV-NS2A-B-3-S138A-REV | GCC AAA TCA ATT GGC ACT G |
| DENV-NS2A-FWD | TAA CTC GAG TCT AGA GGG |
| DENV-NS2A-REV | CCT TTT CTT GCT GGT TCT TG |
| YFV-NS2A-FWD | TAA CTC GAG TCT AGA GGG |
| YFV-NS2A-REV | CCT TCG CCC AAA TAT GCG |
| JEV-NS2A-FWD | TAA CTC GAG TCT AGA GGG |
| JEV-NS2A-REV | TCT CTT CTT GTT TGG GTT G |
| MVEV-NS2A-FWD | TAA CTC GAG TCT AGA GGG |
| MVEV-NS2A-REV | TCT CTT CTT GTT AGG GTT G |
| WNV-NS2A-FWD | TAA CTC GAG TCT AGA GGG |
| WNV-NS2A-REV | GCG TTT ACG GTT GGG ATC |
| KFDV-NS2A-FWD | TAA CTC GAG TCT AGA GGG C |
| KFDV-NS2A-REV | CCG TCT GTT CCT TCG CTC |
| LGTV-NS2A-FWD | TAA CTC GAG TCT AGA GGG CCC |
| LGTV-NS2A-REV | ACG TCT CCC CCT GGA GGC |
| POWV-NS2A-FWD | TAA CTC GAG TCT AGA GGG CCC G |
| POWV-NS2A-REV | CCG TCT GCC ACG ACC CCC |
| TBEV-NS2A-FWD | TAA CTC GAG TCT AGA GGG |
| TBEV-NS2A-REV | CCG TCT CCT TCC ATG GAT |
