# Supplementary material for: Zika virus produces two isoforms of the NS2A protein that play mutually exclusive roles in viral replication and virion assembly
Source: PLoS Pathog. 2026 Oct 12;22(10):e1014609. doi: 10.1371/journal.ppat.1014609 (PMC13671652; doi:10.1371/journal.ppat.1014609)
Supplement: Supplementary file 4 [file ppat.1014609.s003.docx]

**Table S3. gBlock sequence used to clone NS2A-2B-3 overexpression vector.**

| **gBlock** | **Sequence (5’-3’)** |
| --- | --- |
| SPG-NS1(16)-HA-NS2A gBlock | aatctaGCTAGCatgggcgtgaaggtgctgttcgccctgatctgcatcgccgtggccgaggccCCCAGGAAAGAACCAGAAAGCAACTTAGTAAGGTCAATGGTGACTGCAGGCAGCGGCTACCCATACGACGTGCCAGACTACGCCGGCGGCGGCGGATCAACTGATCACATGGACCACTTC |
