# Supplementary material for: Zika virus produces two isoforms of the NS2A protein that play mutually exclusive roles in viral replication and virion assembly
Source: PLoS Pathog. 2026 Oct 12;22(10):e1014609. doi: 10.1371/journal.ppat.1014609 (PMC13671652; doi:10.1371/journal.ppat.1014609)
Supplement: Supplementary file 5 [file ppat.1014609.s004.docx]

**Table S4. RT-qPCR primers and probe sets.**

| **Primer Name** | **Sequence (5’-3’)** |
| --- | --- |
| (+)RNA FOR ZV-5895 FOR | AGA TGC CTA AAG CCG GTC ATA CT |
| (+)RNA REV (Tag5) | CTG GAG TCG TAG ATC CTA CCG C |
| (+)RNA probe (5938–5962) (Cy5) | TGG CTG GAC CCA TGC CTG TCA CAC A |
| Vero-betaactin-FWD | GTGGCATCCATGAAACTACCT |
| Vero-betaactin-REV | GTAATCTCCTTCTGCATCCTGTC |
| Vero-betaactin-probe (Hex) | ATGAAGTGTGACGTGGACATCCGT |
