# Supplementary material for: Zika virus produces two isoforms of the NS2A protein that play mutually exclusive roles in viral replication and virion assembly
Source: PLoS Pathog. 2026 Oct 12;22(10):e1014609. doi: 10.1371/journal.ppat.1014609 (PMC13671652; doi:10.1371/journal.ppat.1014609)
Supplement: Supplementary file 6 [file ppat.1014609.s012.docx]

**Supporting Information**

**Supplementary Methods**

***Western Blot for Polyprotein Processing***

Western blotting was completed as described in the main text (*see* Western Blots). Primary

antibodies of Rabbit anti-NS5 (GTX133312 from Genetex), Rabbit anti-NS1 (GTX133307 from

Genetex), Rabbit anti-NS2B (GTX133308 from Genetex), and Rat anti-NS3 (kindly provided by

Dr. Laurent Chatel-Chaix, INRS-Institut Armand-Frappier) were diluted 1:1000 in 2% milk in

PBS-0.1% Tween-20 while Rabbit anti-HA (3724S from Cell Signaling), and Mouse anti-

GAPDH (sc-365062 from Santa Cruz) were diluted at 1:2000. Secondaries include Goat anti-

Rabbit Peroxidase Conjugate (A6154-1ML from Sigma Aldrich), Goat anti-Rat Peroxidase

Conjugate (SAB3700541-2MG from Millipore), and Goat anti-Mouse Peroxidase Conjugate

(A4416-1ML from Sigma Aldrich) diluted 1:5000 in PBS-0.1% Tween-20. Membranes were

washed with PBS-0.1% Tween-20, incubated with Cytiva Amersham (RPN2235) for 5 minutes

(min), and then imaged using a BioRad ChemiDoc.

**Reverse transcription quantitative polymerase chain reaction (RT-qPCR) analyses**

At 10 h post-electroporation, Vero cells were washed 5 times with PBS before replacing with fresh DMEM media supplemented with 15 mM HEPES (Wisent, Inc.), 2 % FBS, 1% nonessential amino acids (Gibco), and 1% l-glutamine (Gibco). At 38 h post-electroporation, cellular supernatant was collected and spun at 3000 × g for 10 min at 4 °C to remove cellular debris before 250 μL of clarified supernatant was added to 750 µL of TRIzol LS Reagent (ThermoFisher). Meanwhile, cells were washed 1 time with PBS and harvested in 500 μL of TRIzol (ThermoFisher). RNA was extracted from the supernatant and cells according to the manufacturer’s instructions and were resuspended in 15 µL of RNase-free water. Plates for RT-qPCR were prepared according to iTaq Universal Probes One-Step kit (BioRad), using primer/probe sets for Vero actin and the ZIKV positive-strand at 300 nM and 75 nM, respectively (**Table S4**) [56]. For each well, either 1 µg of total cellular RNA or 7 µL of supernatant RNA was used, with each sample run in duplicate, with the data representative of three independent biological replicates. Runs were performed on a CFX96 Real-Time System (BioRad) with the following conditions: 50ºC for 10 min, 95ºC for 3 min, and then 40 cycles of 95º for 15 s and 64ºC for 30 s.

**Protein Detection in Supernatant and Cellular Lysates**

At 38 h post-electroporation, cellular supernatant was collected and spun at 3000 × g for 10 min

at 4 °C, before being passed through a 0.45 µm filter. Filtered supernatants were added to a 20%

sucrose cushion (5 mL) and centrifuged at 28,000 rpm for 4 h at 4 °C in a Beckman XE-90

ultracentrifuge with a SW28 Ti Swing-Bucket Rotor (Beckman). Following centrifugation, the

pellet was resuspended in 200 μL PBS overnight at 4°C. Cellular lysates were washed 1 time

with PBS before collection with 200 µL of n-dodecyl ß-D-maltoside (DDM) lysis buffer [0.5%

DDM (Sigma Aldrich), 0.05% 2 M NaCl, 0.04% 0.5 M Tris-Cl pH 7.5, with 1 cOmplete mini,

EDTA-free protease inhibitor cocktail tablet (Roche Diagnostics)] and spun head-over-tail for 1

h at 4 °C to clear the lysates. For SDS-PAGE, either 120,000 μg of cellular lysate or 100 µL of

supernatant sample was loaded. Data shown is representative of two independent biological

replicates.
