# Supplementary material for: Zika virus produces two isoforms of the NS2A protein that play mutually exclusive roles in viral replication and virion assembly
Source: PLoS Pathog. 2026 Oct 12;22(10):e1014609. doi: 10.1371/journal.ppat.1014609 (PMC13671652; doi:10.1371/journal.ppat.1014609)
Supplement: Supplementary file 7 [file ppat.1014609.s013.docx]

**Response to Reviewers' Comments:**

**Reviewer #1:**

***Summary:*** This manuscript describes the presence of a novel version of the orthoflavirus NS2A protein. This protein has been difficult to study because there are no antibodies to it. Previous work by others has shown that NS2A has roles in both viral replication and assembly. This manuscript uses an NS2A-tagged genomic ZIKV RNA to show that NS2A is actually present in two forms in cells, one full-length and one C-terminal truncated isoform that they term NS2Aalpha. This truncated isoform of NS2A has previously been identified by others in YFV, JEV, and TMUV, suggesting conservation. Indeed, this study proves conservation for NS2Aalpha among a number of orthoflaviviruses. This study also shows that the NS2Aalpha form is made by the NS2B-NS3 protease, and this, along with cleavage site amino acids, was carefully demonstrated using protease cleavage assays in cells. Then, convincing virologic assays using subgenomic and genomic ZIKV RNA molecules were presented that reveal that preventing formation of NS2Aalpha does NOT alter replication but does prevent the production of infectious virions. Overall, the manuscript is well-written and the data convincing, with the conclusions appropriately supported.

***Major comments:***

• While the work showed that NS2Aalpha is important for viral spread, a little bit more experimental insight into how this happening would significantly increase the impact of this work. While clear that it is not required for formation of replication organelles, what is it required for? For example, do the different versions of NS2A bind viral RNA different? Or is it required for Capsid-RNA binding?

We appreciate the insightful inquiry from the reviewer, and we have addressed this in the revised manuscript. Briefly, we used RT-qPCR to assess the amount of viral genomic RNA in the supernatant of cells electroporated with wild-type and NS2Aα biogenesis mutant viral RNAs (**Figure S6**). Moreover, we also attempted to assess subviral particle production by assessing capsid and envelope levels in cell lysates at 38 h post-electroporation in Vero cells. Briefly, while we were able to detect viral RNA, capsid and envelope proteins in the cell lysates and supernatants from WT viral RNA electroporated cells, we were unable to detect viral RNA or capsid proteins in the cell supernatants of any of the NS2Aα biogenesis mutants. However, in some cases, we were able to detect envelope proteins in the cell supernatants. This was not due to a lack of active viral RNA replication since both capsid and envelope proteins could be detected in the cell lysates from all the replication-competent mutants. This data suggests that the defects in cell spread and infectious viral particle production are likely due to a defect in genome packaging, rather than a defect in specific infectivity. This data are now included in the manuscript as **Figure S6**.

• Also, insights into when this version is made during the lifecycle would be informative. If you prevent assembly, for example by making a mutation in NS3 that ablates assembly, is it still made? I wonder if the protease in the vRO or outside the vRO is important for this process

This is an interesting question and one that we have also considered ourselves. Based on our data in Vero cells (**Figure 1**) and in A549 and C6/26 cells (**Supplementary Figure 2**), the NS2Aα isoform is the predominant isoform of the protein throughout infection and is present well before (presumably) viral assembly and/or assembly sites are established. As such, NS2Aα appears to be present throughout the life cycle, and forms independent of assembly site formation. In future, it may be interesting to understand a reciprocal question - what *prevents* NS2Aα formation? As presumably, a pool of full-length NS2A is required for viral RNA replication at the replication organelle and it may be that preventing NS2Aα formation is how this pool is established.

***Minor Comments:***

 • Figure 2 would be a little easier to follow if the stars in Figure 2A had arrows and labels indicating R226 and S1D.

We have updated this figure accordingly.

• For Fig 5B and Fig 5C, showing the individual data points of the independent biological replicates in the bar graphs would be helpful.

Thanks for the suggestion, we have included these in the updated bar graphs for this figure (*see* **Figure 6B and 6C**).

**Reviewer #2:**

***Summary:*** In the manuscript Landry et al the authors analyze the role of Zika virus (ZIKV) NS2A and it’s C-terminally truncated variant NS2Aα in both virus replication and virion assembly. The authors used a ZIKV full-length clone expressing NS2A with an internal HA-tag in the NS2A N-terminal region to show that during infection both the NS2A and NS2Aα variant are produced. Further, they showed that the NS2Aα cleavage site is also present in over 60 other orthoflaviviruses and that the production of NS2Aα is dependent on the NS2B-3 protease activity. They analyzed ZIKV NS2Aα cleavage site mutants as well as further ZIKV mutants, some of which had been described previously, for production of replication organelles, RNA replication and production of infectious particles.
Overall, the presented work represents a nice set of experiment, which in most cases were well thought out and carried out. However, several of the results presented here have already been shown either for other orthoflaviviruses like YFV or DENV and also for ZIKV itself, which minimizes the novelty of the story. To make the story more complete, the following points should be addressed.

1. The title of the manuscript is too general. The statements in the title describe what is already known. Relate the title more to ZIKV-specific data.

Thank you for the suggestion, we have updated the manuscript title accordingly.

2. This applies also to the abstract: Line 27: we reveal that two NS2A isoforms are present during orthoflavivirus infection, including both full-length NS2A and a C-terminally truncated isoform, termed NS2Aα – this is already known.

Thank you for the suggestion, we have updated this accordingly.

Same applies to Line 35: suggests that each NS2A isoform plays a unique role in the orthoflaviviral life cycle – also already known. As mentioned above: relate more to ZIKV-specific data.

While we thank the reviewer for the suggestion, but we believe that this indeed a novel finding given that previous studies in YFV (Kummerer and Rice, 2002) demonstrate that NS2A cleavage (and subsequent effects on infectious particle production) do not affect viral RNA accumulation, but they did not directly implicate the full-length NS2A protein to this stage of the viral life cycle. Similarly, research in dengue virus (Xie et al. 2014) suggests that there are “two distinct sets of NS2A molecules” but do not assign specific NS2A isoforms to the stages of viral RNA replication and virion assembly, respectively. Herein, we uniquely demonstrate that preventing formation of full-length NS2A abolishes viral RNA replication in ZIKV; and excitingly, that this impacts the morphology of viral ROs.

3. Line 127: for the pcDNA3.1 expression constructs: is the HA-tag within the N-terminal part of NS2A like it is the case in the full-length clone or at the far N-terminus of NS2A? How is correct insertion into the ER membrane ensured?

We apologize for the confusion here, as indicated in the Materials & Methods section, correct insertion into the ER membrane is ensured through the insertion of a *Gaussia* luciferase signal sequence (SPG) followed by a GSG linker, the HA-tag, and a GGG linker at the N-terminus of the NS2A protein. In the context of the genome, the signal sequence is not required as the C-terminus of NS1 ensures proper insertion of the NS2A protein into the ER membrane.

4. Line 175: the authors state that for 61 out of 62 orthoflaviviruses a putative NS2Aα cleavage motif was identified. Which is the orthoflavivirus for which they did not find the motif?

We did not have significant confidence in the alignment of the Bouboui virus (BOUV, GenBank accession number: NC_033693.1) NS2Aα cleavage site because the cleavage motif that aligned with the other NS2Aα cleavage sites was an “KR | S” motif, while we also observed an upstream “QK | S” motif. While the latter upstream motif seemed more likely given what we know about the NS2Aα cleavage site, since the downstream “KR | S” motif aligned better with the consensus, we didn’t feel confident enough to assign either of these as the definitive NS2Aα cleavage site. As such, we omitted this sequence from our analyses.

5. Analyses of replication organelles: to my opinion this part needs extensive revision.
A general note first: I suggest to describe the studies concerning the interplay of NS2A mutants and replication before describing the RO experiments. This way the phenotypes of the mutants analyzed for RO formation are already known with regard to their replication potential. Thus, please change order of experiments accordingly.

Thank you for this suggestion and we agree that this might make the manuscript easier to digest. In the revised manuscript, we have swapped the order of these experiments such that replication in the context of the subgenomic replicon is carried out first, and then the RO biogenesis phenotypes are described using the pIRO system.

6. Was the pIRO-Z system self-made by the authors based on the information in reference 37 or obtained from the authors of reference 37? Does it contain the 5’ UTR or the GDD mutation? Either one of this (GDD mutation or lack of 5’ UTR) should be the case to ensure block of replication (the authors state in line 251 that analyses can be done in the absence of RNA replication). The respective feature(s) (no 5’ UTR/GDD mutant) should be mentioned in the text/Figure legend (Fig. 5)

Our co-author, Dr. Chris J. Neufeldt, performed his postdoctoral fellowship in the Bartenschlager Lab prior to starting his own research group at Emory University and was a co-author on the original pIRO study (reference 37, Cerikan et al. Cell Rep 2020). As outlined in the original study, pIRO-Z refers to the specific construct that expresses the viral non-structural proteins with a full 3´ UTR but only the CS element from the 5´ UTR (SLA and SLB are deleted, see Figure 3 in Cerikan *et al. Cell Rep* 2020). The construct does encode a wild-type NS5 protein, but as discussed in the reference study and demonstrated in others, the lack of SLA makes this construct replication-incompetent. We have added additional text to the Materials & Methods and in **Figure 6** to clarify these points.

8. Major point: Fig. 5. 7/ Line 254: The authors state that the N130A mutant was severely attenuated in vRO biogenesis. However, Fig. 5c shows that the transfection efficiency for N130A is much lower compared to the other constructs. Hence, this should already result in lower RO formation and the conclusion that RO formation for the N130A is severely attenuated (line 254) cannot be drawn.

We have subsequently performed additional experiments that had higher transfection efficiency for N130A and this is now incorporated into the revised manuscript (*see* **Figure 6**). Briefly, these data demonstrate that even with comparable transfection efficiencies, expression of pIRO constructs with the N130A mutant does not lead to significant vRO formation.

9. The authors state in Mat/Met that they count the number of RO positive cells per 40 cells. However, they rather should count the number of vesicles per cell. Groups might be possible like in reference 37: No vesicles, 2-5 vesicle particles or more than 5 vesicle particles. Counting the number of positive cells per specified number of cells is too much dependent on the transfection efficiency/could simply reflect the transfection efficiency.

Evaluating the number of positive cells was also the metric we used in the original study describing the pIRO system (Cerikan *et al. Cell Rep* 2020). The reason to avoid counting the number of vesicles per cell in the original study and subsequent studies is that the pIRO-Z system shows significant heterogeneity in the number of vesicles per positive cell, even in the wild-type construct. This is likely dependent on several variables that are difficult to control, including the number of plasmids introduced into each cell, leading to different levels of expression and the fact that TEM only looks at a ~80 nm slice of the cell, which may not be representative of the entire cell volume. Given the heterogeneity, reporting vesicle numbers per cell has the potential to create artifacts that do not represent the biology. Thus, through extensive analyses using different versions of this system, we have determined that counting positive cells as those containing vROs or not, is the most practical use of the pIRO system. In the current study, cells were only counted as positive if they had more than 4 vesicles, eliminating the need for separation into groups of 2-5 or 5 or more. Notably, no cells were found with precisely 3 vROs in this data set, and while a handful of cells that contained only 2 vROs were counted as negative, these cells were removed because visualization by two authors could not confirm that both vesicles were *bona fide* vROs. We have added additional text to the methods section to clarify our scoring system.

10. Statistics for Fig 5C and 5D is missing.

We apologize for this oversight and now include the statistics in this Figure (*see* **Figure 6**).

11. Is the vesicle diameter different for the vesicles observed from the different constructs?

To further evaluate differences in vRO formation between NS2A mutants, we have now calculated the average vesicle diameter for each mutant and we have now included this data as well as a discussion of this in the revised manuscript (*see* **Figure 6**).

12. Lines 308ff: The authors use V5-NS2A for trans-complementation analyses of RNA replication. Since trans-complementation was negative in all cases - how can the authors ensure that the V5-NS2A is functional with the V5 tag at the N-terminus? How is the correct insertion of NS2A into the ER membrane ensured? This is important to know – actually, the authors state this critical point by themselves in the discussion: Line 413 ff: It is possible that expression of NS2A alone does not result in proper folding and/or topology of NS2A on the ER membrane, resulting in host protease mediated decay intermediates.

We thank the reviewer for this point. While we find that internally V5-tagged NS2A virus is viable with a stable V5-tag, it is indeed not clear that SPG-V5-NS2A expression constructs (and hence our stable cells) allows for the proper NS2A folding and/or topology when expressed in isolation. To this end, we have removed the *trans*-complementation experiments from the revised manuscript.

13. In line with point 12: experiments need to be performed to test whether the assembly defect of YFV NS2Aα cleavage site mutants can be complemented in trans by NS2A. Can the V5-NS2A cell line established here be used to complement the assembly defect for the ZIKV NS2Aα cleavage site mutants (K192A, K193A)? If this is the case this would also show the functionality of the expressed V5-NS2A. If NS2A on it’s own does not allow trans-complementation of the assembly defect, trans-complementation shall be performed with sigNS1-NS2A.

Interestingly, we do not observe any effects on subgenomic replicon-mediated viral RNA replication in the V5-NS2A stable cells. Moreover, we have shown that V5-NS2A can be cleaved *in trans* via co-transfection of HA-NS2A-2B-3 polyprotein. However, our data indicates that V5-NS2A stable cells act as a dominant negative for viral infection, suggesting that they are a dominant negative for virion assembly. As we have not been able to disentangle these results, we have omitted all *trans*-complementation experiments from the current manuscript. This will be the focus of future work.

14. Besides trans-complementation analyses of the assembly deficient ZIKV mutants with full-length NS2A, the authors should also perform trans-complementation analyses with NS2Aα. Both WT NS2Aα as well as with NS2Aα K192A and NS2Aα K193A should be tested (if necessary based on sigNS1-NS2A). This will indicate whether only the cleavage is important for assembly or also the nature of the amino acids in the NS2Aα cleavage site region.

As discussed above, this will be the focus of future research.

15. Are the assembly mutants (K192A, K193A) still able to form subviral particles? The authors should perform WB analyses to detect the envelope proteins in the supernatant (and lack of capsid in comparison to WT virus).

This is an important question and we have now included **Figure S6** where we measured viral RNA, capsid, and envelope proteins in cell supernatants at 38 h post-electroporation.

16. Discussion: Line 433: Interestingly, we found that the NS2A N130A mutation attenuates ZIKV vRO biogenesis, providing support for the importance of NS2A in this process. As mentioned above: I think this conclusion cannot be made since the transfection efficiency was very low for this construct.

We thank the reviewer for pointing this out and believe that the addition of new replicates with higher transfection efficiency negates this issue (*see* **Figure 6 and Figure S5**).

***Major Comments:***

1. Revalidation of the N130A mutant in the vRO assay. Due to the low transfection efficiency the statement made in the manuscript does not seem appropriate. The authors should either provide supporting experiments for their statement or rephrase their statement.

As discussed above, we have provided additional replicates that support the conclusions made herein.

2. The authors should perform trans-complementation analyses of the assembly-deficient ZIKV mutants (K192A, K193A) to see whether the assembly defect can be complemented by full-length NS2A, NS2Aα or NS2Aα with K192A or K193A. To do this, they should try their V5-NS2A expressing cell line. However, if this cell line does not allow trans-complementation (possibly due to improper NS2A folding/insertion into the ER membrane), the respective analyses should also be performed based on expression of sigNS1-NS2A or sigNS1-NS2Aα, for which proper insertion in the ER membrane is known (see also point 14 in summary).

As discussed above, we have removed the *trans*-complementation experiments from this study, and this will be the focus of future research.

3. The authors should analyse whether the assembly deficient ZIKV mutants are still able to produce subviral particles (see also point 15 in summary).

As discussed above, this is now included in the revised manuscript (**Figure S6**).

***Minor comments:***

1. Line: 58 and 59: little is known about form and function of NS2A – not sure that one can says this. Several NS2A studies have been described - rephrase it to tone it down a bit.

We have rephrased accordingly.

2. Fig.1 legend: add: using anti-HA antibody

This is now included in the Figure legend.

3. Fig. 6: E and F – use same color code and ideally also same order.

We have fixed the order and colour coding herein.

4. Fig. S4 Legend: please explain used abbreviation CTD also in legend (that it corresponds to C terminal domain of NS2A in this case)

Thanks, we have now made sure this was clear throughout and defined the abbreviation in the figure legend.

5. Add supplementary figure showing IF pictures (staining against NS3) after transfection of the constructs shown in Fig 5C/D.

These data have now been included (*see* **Figure S5**).

5. Discussion: when talking about virus assembly, the insights of the following paper should be included, especially also the interaction between NS2 An and UFL1.
<https://journals.asm.org/doi/10.1128/jvi.00654-25>

Thanks for the suggestion, we have incorporated this into the discussion.

6. Line 382 ff: NS2A remains one of the least characterized orthoflavivirus proteins, both biochemically and functionally, precluding a detailed mechanistic understanding of its contribution(s) to the viral life cycle – this seems also a little bit overstated.

We have adjusted this statement accordingly.

7. Legend Figure 5: (C) Transfection efficiency and (D) percentage of vRO positive cells based on immunofluorescence staining for viral NS3 of pIRO-Z-transected cells.
> Shouldn't IF staining only refer to transfection efficiency (Fig. 5c)? Weren‘t the vRO postive cells determined by electron microscopy? Please rephrase accordingly.

Thank you for pointing out this oversight. We have adjusted the text accordingly.

8. Line 248: We generated a series of mutations – based on what have they been chosen? Previous publications? What was the formerly described phenotype for them?

Thanks for bringing this to our attention, we have done our best to make sure this is clearer in the current revision.

**Reviewer #3:**

***Summary:*** Landry et al. identified a truncated version of NS2A, termed NS2A⍺ in orthoflavivirus infected cells, especially those transmitted by Aedes sp of mosquito including Zika virus (ZIKV). The authors characterized the NS2A⍺ isoform expression with a HA-tagged NS2A (HA-NS2A) in the context of ZIKV genome as well as exogenous expression of NS2A-2B-3 in different cell lines. Further studies established the role of the viral protease NS2B-3 in the generation of NS2A⍺. The cleavage of NS2A⍺ was independent of the NS2A-2B cleavage and the cleavage motif is conserved across other orthoflaviviruses. The authors examined the function of NS2A⍺ in ZIKV life cycle and identified its role in infectious virus particle production by plaque assays.

Overall, this is a novel study, and a well-written manuscript. The study demonstrates novel findings for ZIKV non-structural protein NS2A and its isoform, NS2A⍺. All the claims of the authors are backed up with high quality data. However, the mechanistic insight into the role of NS2A⍺ in infectious particle production is poorly developed. Addressing the following issues would improve the overall quality of the study and elucidate the role of NS2A⍺ in infectious virus production.

**Major Comments:**

1. The authors show that mutations abrogating the production of NS2A⍺ leading to non-detectable levels of infectious virus particle production. However, it is unclear what is the underlying mechanism, whether particle assembly, release of infectious virions, or the specific infectivity of the virus particles are affected by abolishing NS2A⍺ processing. We strongly encourage the authors to address this important point and examine how K192A and K192A affect the levels of intracellular virions (freeze and thaw assay) and the ratio of released viral RNA to viral titres. Use of TEM to examine the number and morphology of virus particles in cells electroporated with the mutants would also give a better understanding regarding the role of NS2A⍺ during virus assembly.

Thanks for the suggestion. To better elucidate a mechanism, we assessed viral RNA, capsid and envelope levels in cells and supernatants at 38 h post-electroporation. This data is now included in **Figure S6**.

2. The replication kinetics of HA-NS2A ZIKV, is shown only in the context of replicons in Figure 6. Including the replication kinetics of the constructs used for viral spread assay in fig 7 will provide a direct comparison of replication vs particle production in a full-length virus context (as only vRO positive cell number not actual replication levels were measured in figure 5). Negative strand specific qPCR can be utilized to discriminate viral replication from input RNA.

As discussed above, we have now assessed viral RNA, capsid and envelope levels in cells and supernatants at 38 h post-electroporation. This data is now included in **Figure S6**.

3. The TEM data in figure 5 does not have any quantification on the size of the replication organelles or their number in the mutants vs wild type. According to data in figure 5D there is only a 33% reduction in vRO positive cells in K192A and K192A mutant infected cells, which even disappears if we normalize the transfection efficiency. This data does not correlate well with the TEM data which shows a more drastic phenotype. Proper quantification of the TEM data might put all the data in proper context.

This data has now been added to the manuscript (**Figure 6**). To clarify, we do not propose that the K192A mutant affects vRO formation. When taking transfection efficiency into account, only the N130A mutant has a meaningful impact on the number of vRO-positive cells (**Figure 6**).

4. While the authors show that NS2A⍺ is also produced in C6/36 cells (Fig S2), no replication or particle production data is shown for insect cells. This would be of interest, to see if the functional role of NS2A⍺ in infectious virus production is extended to insect cells as well.

This is an excellent suggestion and will be the focus of future studies.

***Minor Comments:***

 1. In figure 6, writing the cell line used for each sub-figure will make it clear to the reader about the conditions of the experiment, such as trans-complementation of NS2A in 6C.

Thanks for the suggestion, we have done our best to make it clear what cell line is used from each experiment throughout the revised manuscript.

2. A schematic diagram of the sub-genomic replicons and WT constructs should be included wherever used (Figure 6, 7), specially including the location of the luciferase reporter gene.

Great suggestion, we have now included this throughout the manuscript.

3. In fig 6, it will help the reader if the y-axis is clearly defined, rather than just %, it would be beneficial to mention % of or relative to what.

Apologies for this oversight, we have now better defined the axes in **Figure 6.**

4. In figure 4A, some viruses such as JEV, MVEV, DENV, LGTV do not show production of NS2A at all, and only of NS2A⍺. Is this a representative of three biological repeats? Similarly, in fig S2-3, NS2A production in HA-NS2A ZIKV infected A549 cells is very low specially compared to C6/36 cells and NS2A-2B-3 over pression in cells. Can you speculate if that is a cell specific phenotype and if it is consistently seen with HA-NS2A ZIKV infection?

We appreciate the detailed thought that the reviewer put into this question. The displayed Western blots are indeed representative of 3 independent biological replicates and in some cases there does seem to be more “complete cleavage” of NS2A to the NS2Aα isoform. While we have observed some cell type-dependent differences in the NS2A to NS2Aα ratio, we are hesitant to comment on cell-specific differences with regards to the other viruses as the polyprotein over-expression experiments were completed in HEK 293T cells (due to their high transfection efficiency), while we primarily used Vero and BHK-21 cells for ZIKV replication and/or infection experiments (as well as C6/36 and A549 cells, *see* **Figures 1, 5, 7** and **Figure S2**). Nonetheless, in all cases, we observe that NS2Aα is the predominant isoform during ZIKV infection and it remains unclear whether full-length NS2A is required either only transiently or perhaps only in small quantities (i.e. only within ROs). We anticipate that further precise mechanistic insight into each of the NS2A isoforms will help clarify this in future studies.
