# Supplementary material for: Zika virus produces two isoforms of the NS2A protein that play mutually exclusive roles in viral replication and virion assembly
Source: PLoS Pathog. 2026 Oct 12;22(10):e1014609. doi: 10.1371/journal.ppat.1014609 (PMC13671652; doi:10.1371/journal.ppat.1014609)
Supplement: Peer review history

# Author comment: Author response to Decision Letter 0

**DOI:** 10.1371/journal.ppat.1014609.r001

**Transfer Alert**

This paper was transferred from another journal. As a result, its full editorial history (including decision letters, peer reviews and author responses) may not be present.

11 Jan 2026

# Peer review: Decision Letter 0

**DOI:** 10.1371/journal.ppat.1014609.r002

**Authors:** Andrea V. Gamarnik, Sonja M. Best

11 Feb 2026

Orthoflaviviruses produce two isoforms of the NS2A protein that play disparate roles in viral replication and virion assembly

PLOS Pathogens

Dear Dr. Sagan,

Thank you for submitting your manuscript to PLOS Pathogens. After careful consideration, we feel that it has merit but does not fully meet PLOS Pathogens's publication criteria as it currently stands. Therefore, we invite you to submit a revised version of the manuscript that addresses the points raised during the review process.

Please submit your revised manuscript by Apr 12 2026 11:59PM. If you will need more time than this to complete your revisions, please reply to this message or contact the journal office at plospathogens@plos.org. When you're ready to submit your revision, log on to https://www.editorialmanager.com/ppathogens/ and select the 'Submissions Needing Revision' folder to locate your manuscript file.

Please include the following items when submitting your revised manuscript:

* A letter that responds to each point raised by the editor and reviewer(s). You should upload this letter as a separate file labeled 'Response to Reviewers'. This file does not need to include responses to any formatting updates and technical items listed in the 'Journal Requirements' section below.

* A marked-up copy of your manuscript that highlights changes made to the original version. You should upload this as a separate file labeled 'Revised Manuscript with Track Changes'.

* An unmarked version of your revised paper without tracked changes. You should upload this as a separate file labeled 'Manuscript'.

If you would like to make changes to your financial disclosure, competing interests statement, or data availability statement, please make these updates within the submission form at the time of resubmission. Guidelines for resubmitting your figure files are available below the reviewer comments at the end of this letter.

We look forward to receiving your revised manuscript.

Kind regards,

Andrea Gamarnik

Academic Editor

PLOS Pathogens

Sonja Best

Section Editor

PLOS Pathogens

Editor-in-Chief

PLOS Pathogens

orcid.org/0000-0003-2946-9497

Editor-in-Chief

PLOS Pathogens

orcid.org/0000-0002-7699-2064

**Additional Editor Comments:**

The manuscript examines the function of NS2A⍺ in the ZIKV life cycle and identifies its role in the production of infectious virus particles. The study provides novel insights into the non-structural protein NS2A and its isoform NS2A⍺.

Overall, all three reviewers find the work well executed, with conclusions that are appropriately supported by the data, and they provide important suggestions to further strengthen the manuscript. It will be important that the authors carefully address the comments of the reviewers. In particular, the suggestions made by Reviewer 1, as well as the major points raised by Reviewers 2 and 3. I do not think it is necessary to specifically discriminate minus-strand RNA synthesis as indicated by reviewer 3.

**Journal Requirements:**

If the reviewer comments include a recommendation to cite specific previously published works, please review and evaluate these publications to determine whether they are relevant and should be cited. There is no requirement to cite these works unless the editor has indicated otherwise.

1) We ask that a manuscript source file is provided at Revision. Please upload your manuscript file as a .doc, .docx, .rtf or .tex. If you are providing a .tex file, please upload it under the item type u2018LaTeX Source Fileu2019 and leave your .pdf version as the item type u2018Manuscriptu2019.

2) Please provide an Author Summary. This should appear in your manuscript between the Abstract (if applicable) and the Introduction, and should be 150-200 words long. The aim should be to make your findings accessible to a wide audience that includes both scientists and non-scientists. Sample summaries can be found on our website under Submission Guidelines:

https://journals.plos.org/plospathogens/s/submission-guidelines#loc-parts-of-a-submission

3) We do not publish any copyright or trademark symbols that usually accompany proprietary names, eg ©,  ®, or TM  (e.g. next to drug or reagent names). Therefore please remove all instances of trademark/copyright symbols throughout the text, including:

- TM on page: 30.

4) Please upload all main figures as separate Figure files in .tif or .eps format. For more information about how to convert and format your figure files please see our guidelines:

https://journals.plos.org/plospathogens/s/figures

5) We notice that your supplementary Figures, and Tables are included in the manuscript file. Please remove them and upload them with the file type 'Supporting Information'. Please ensure that each Supporting Information file has a legend listed in the manuscript after the references list.

6) Some material included in your submission may be copyrighted. According to PLOSu2019s copyright policy, authors who use figures or other material (e.g., graphics, clipart, maps) from another author or copyright holder must demonstrate or obtain permission to publish this material under the Creative Commons Attribution 4.0 International (CC BY 4.0) License used by PLOS journals. Please closely review the details of PLOSu2019s copyright requirements here: PLOS Licenses and Copyright. If you need to request permissions from a copyright holder, you may use PLOS's Copyright Content Permission form.

Please respond directly to this email and provide any known details concerning your material's license terms and permissions required for reuse, even if you have not yet obtained copyright permissions or are unsure of your material's copyright compatibility. Once you have responded and addressed all other outstanding technical requirements, you may resubmit your manuscript within Editorial Manager.

Potential Copyright Issues:

i) Figure 2A. Please confirm whether you drew the images / clip-art within the figure panels by hand. If you did not draw the images, please provide (a) a link to the source of the images or icons and their license / terms of use; or (b) written permission from the copyright holder to publish the images or icons under our CC BY 4.0 license. Alternatively, you may replace the images with open source alternatives. See these open source resources you may use to replace images / clip-art:

- https://commons.wikimedia.org

- https://openclipart.org/.

7) In the online submission form, you indicated that All data needed to evaluate the conclusions in the paper are present in the paper and/or the Supplementary Materials. Requests for resources/materials should be directed to S.M.S (selena.sagan@ubc.ca) and C.J.N (c.neufeldt@emory.edu).. All PLOS journals now require all data underlying the findings described in their manuscript to be freely available to other researchers, either

1. In a public repository

2. Within the manuscript itself

3. Uploaded as supplementary information.

This policy applies to all data except where public deposition would breach compliance with the protocol approved by your research ethics board. If your data cannot be made publicly available for ethical or legal reasons (e.g., public availability would compromise patient privacy), please explain your reasons by return email and your exemption request will be escalated to the editor for approval. Your exemption request will be handled independently and will not hold up the peer review process, but will need to be resolved should your manuscript be accepted for publication. One of the Editorial team will then be in touch if there are any issues.

8) Please amend your detailed Financial Disclosure statement. This is published with the article. It must therefore be completed in full sentences and contain the exact wording you wish to be published.

1) State the initials, alongside each funding source, of each author to receive each grant. For example: "This work was supported by the National Institutes of Health (####### to AM; ###### to CJ) and the National Science Foundation (###### to AM)."

2) State what role the funders took in the study. If the funders had no role in your study, please state: "The funders had no role in study design, data collection and analysis, decision to publish, or preparation of the manuscript."

3) If any authors received a salary from any of your funders, please state which authors and which funders..

If you did not receive any funding for this study, please simply state: u201cThe authors received no specific funding for this work.u201d

**Reviewers' Comments:**

Reviewer's Responses to Questions

**Part I - Summary**

Please use this section to discuss strengths/weaknesses of study, novelty/significance, general execution and scholarship.

Reviewer #1: This manuscript describes the presence of a novel version of the orthoflavirus NS2A protein. This protein has been difficult to study because there are no antibodies to it. Previous work by others has shown that NS2A has roles in both viral replication and assembly. This manuscript uses an NS2A-tagged genomic ZIKV RNA to show that NS2A is actually present in two forms in cells, one full-length and one C-terminal truncated isoform that they term NS2Aalpha. This truncated isoform of NS2A has previously been identified by others in YFV, JEV, and TMUV, suggesting conservation. Indeed, this study proves conservation for NS2Aalpha among a number of orthoflaviviruses. This study also shows that the NS2Aalpha form is made by the NS2B-NS3 protease, and this, along with cleavage site amino acids, was carefully demonstrated using protease cleavage assays in cells. Then, convincing virologic assays using subgenomic and genomic ZIKV RNA molecules were presented that reveal that preventing formation of NS2Aalpha does NOT alter replication but does prevent the production of infectious virions. Overall, the manuscript is well-written and the data convincing, with the conclusions appropriately supported.

Reviewer #2: In the manuscript Landry et al the authors analyze the role of Zika virus (ZIKV) NS2A and it’s C-terminally truncated variant NS2Aα in both virus replication and virion assembly.

The authors used a ZIKV full-length clone expressing NS2A with an internal HA-tag in the NS2A N-terminal region to show that during infection both the NS2A and NS2Aα variant are produced. Further, they showed that the NS2Aα cleavage site is also present in over 60 other orthoflaviviruses and that the production of NS2Aα is dependent on the NS2B-3 protease activity. They analyzed ZIKV NS2Aα cleavage site mutants as well as further ZIKV mutants, some of which had been described previously, for production of replication organelles, RNA replication and production of infectious particles.

Overall, the presented work represents a nice set of experiment, which in most cases were well thought out and carried out. However, several of the results presented here have already been shown either for other orthoflaviviruses like YFV or DENV and also for ZIKV itself, which minimizes the novelty of the story. To make the story more complete, the following points should be addressed.

1. The title of the manuscript is too general. The statements in the title describe what is already known. Relate the title more to ZIKV-specific data.

2. This applies also to the abstract: Line 27: we reveal that two NS2A isoforms are present during orthoflavivirus infection, including both full-length NS2A and a C-terminally truncated isoform, termed NS2Aα – this is already known.

Same applies to Line 35: suggests that each NS2A isoform plays a unique role in the orthoflaviviral life cycle – also already known.

As mentioned above: relate more to ZIKV-specific data.

3. Line 127: for the pcDNA3.1 expression constructs: is the HA-tag within the N-terminal part of NS2A like it is the case in the full-length clone or at the far N-terminus of NS2A? How is correct insertion into the ER membrane ensured?

4. Line 175: the authors state that for 61 out of 62 orthoflaviviruses a putative NS2Aα cleavage motif was identified. Which is the orthoflavivirus for which they did not find the motif?

5. Analyses of replication organelles: to my opinion this part needs extensive revision.

A general note first: I suggest to describe the studies concerning the interplay of NS2A mutants and replication before describing the RO experiments. This way the phenotypes of the mutants analyzed for RO formation are already known with regard to their replication potential. Thus, please change order of experiments accordingly.

6. Was the pIRO-Z system self-made by the authors based on the information in reference 37 or obtained from the authors of reference 37? Does it contain the 5’ UTR or the GDD mutation? Either one of this (GDD mutation or lack of 5’ UTR) should be the case to ensure block of replication (the authors state in line 251 that analyses can be done in the absence of RNA replication). The respective feature(s) (no 5’ UTR/GDD mutant) should be mentioned in the text/Figure legend (Fig. 5)

8. Major point: Fig. 5. 7/ Line 254: The authors state that the N130A mutant was severely attenuated in vRO biogenesis. However, Fig. 5c shows that the transfection efficiency for N130A is much lower compared to the other constructs. Hence, this should already result in lower RO formation and the conclusion that RO formation for the N130A is severely attenuated (line 254) cannot be drawn.

9. The authors state in Mat/Met that they count the number of RO positive cells per 40 cells. However, they rather should count the number of vesicles per cell. Groups might be possible like in reference 37: No vesicles, 2-5 vesicle particles or more than 5 vesicle particles. Counting the number of positive cells per specified number of cells is too much dependent on the transfection efficiency/could simply reflect the transfection efficiency.

10. Statistics for Fig 5C and 5D is missing.

11. Is the vesicle diameter different for the vesicles observed from the different constructs?

12. Lines 308ff: The authors use V5-NS2A for trans-complementation analyses of RNA replication. Since trans-complementation was negative in all cases - how can the authors ensure that the V5-NS2A is functional with the V5 tag at the N-terminus? How is the correct insertion of NS2A into the ER membrane ensured?

This is important to know – actually, the authors state this critical point by themselves in the discussion: Line 413 ff: It is possible that expression of NS2A alone does not result in

proper folding and/or topology of NS2A on the ER membrane, resulting in host protease

mediated decay intermediates.

13. In line with point 12: experiments need to be performed to test whether the assembly defect of YFV NS2Aα cleavage site mutants can be complemented in trans by NS2A. Can the V5-NS2A cell line established here be used to complement the assembly defect for the ZIKV NS2Aα cleavage site mutants (K192A, K193A)? If this is the case this would also show the functionality of the expressed V5-NS2A. If NS2A on it’s own does not allow trans-complementation of the assembly defect, trans-complementation shall be performed with sigNS1-NS2A.

14. Besides trans-complementation analyses of the assembly deficient ZIKV mutants with full-length NS2A, the authors should also perform trans-complementation analyses with NS2Aα. Both WT NS2Aα as well as with NS2Aα K192A and NS2Aα K193A should be tested (if necessary based on sigNS1-NS2A). This will indicate whether only the cleavage is important for assembly or also the nature of the amino acids in the NS2Aα cleavage site region.

15. Are the assembly mutants (K192A, K193A) still able to form subviral particles? The authors should perform WB analyses to detect the envelope proteins in the supernatant (and lack of capsid in comparison to WT virus).

16. Discussion: Line 433: Interestingly, we found that the NS2A N130A mutation attenuates ZIKV vRO biogenesis, providing support for the importance of NS2A in this process

As mentioned above: I think this conclusion cannot be made since the transfection efficiency was very low for this construct.

Reviewer #3: Landry et al. identified a truncated version of NS2A, termed NS2A⍺ in orthoflavivirus infected cells, especially those transmitted by Aedes sp of mosquito including Zika virus (ZIKV). The authors characterized the NS2A⍺ isoform expression with a HA-tagged NS2A (HA-NS2A) in the context of ZIKV genome as well as exogenous expression of NS2A-2B-3 in different cell lines. Further studies established the role of the viral protease NS2B-3 in the generation of NS2A⍺. The cleavage of NS2A⍺ was independent of the NS2A-2B cleavage and the cleavage motif is conserved across other orthoflaviviruses. The authors examined the function of NS2A⍺ in ZIKV life cycle and identified its role in infectious virus particle production by plaque assays.

Overall, this is a novel study, and a well-written manuscript. The study demonstrates novel findings for ZIKV non-structural protein NS2A and its isoform, NS2A⍺. All the claims of the authors are backed up with high quality data. However, the mechanistic insight into the role of NS2A⍺ in infectious particle production is poorly developed. Addressing the following issues would improve the overall quality of the study and elucidate the role of NS2A⍺ in infectious virus production.

**********

**Part II – Major Issues: Key Experiments Required for Acceptance**

Please use this section to detail the key new experiments or modifications of existing experiments that should be absolutely required to validate study conclusions.

Generally, there should be no more than 3 such required experiments or major modifications for a "Major Revision" recommendation. If more than 3 experiments are necessary to validate the study conclusions, then you are encouraged to recommend "Reject".

Reviewer #1: • While the work showed that NS2Aalpha is important for viral spread, a little bit more experimental insight into how this happening would significantly increase the impact of this work. While clear that it is not required for formation of replication organelles, what is it required for? For example, do the different versions of NS2A bind viral RNA different? Or is it required for Capsid-RNA binding?

• Also, insights into when this version is made during the lifecycle would be informative. If you prevent assembly, for example by making a mutation in NS3 that ablates assembly, is it still made? I wonder if the protease in the vRO or outside the vRO is important for this process

Reviewer #2: 1. Revalidation of the N130A mutant in the vRO assay. Due to the low transfection efficiency the statement made in the manuscript does not seem appropriate. The authors should either provide supporting experiments for their statement or rephrase their statement.

2. The authors should perform trans-complementation analyses of the assembly-deficient ZIKV mutants (K192A, K193A) to see whether the assembly defect can be complemented by full-length NS2A, NS2Aα or NS2Aα with K192A or K193A. To do this, they should try their V5-NS2A expressing cell line. However, if this cell line does not allow trans-complementation (possibly due to improper NS2A folding/insertion into the ER membrane), the respective analyses should also be performed based on expression of sigNS1-NS2A or sigNS1-NS2Aα, for which proper insertion in the ER membrane is known (see also point 14 in summary).

3. The authors should analyse whether the assembly deficient ZIKV mutants are still able to produce subviral particles (see also point 15 in summary).

Reviewer #3: 1. The authors show that mutations abrogating the production of NS2A⍺ leading to non-detectable levels of infectious virus particle production. However, it is unclear what is the underlying mechanism, whether particle assembly, release of infectious virions, or the specific infectivity of the virus particles are affected by abolishing NS2A⍺ processing. We strongly encourage the authors to address this important point and examine how K192A and K192A affect the levels of intracellular virions (freeze and thaw assay) and the ratio of released viral RNA to viral titres. Use of TEM to examine the number and morphology of virus particles in cells electroporated with the mutants would also give a better understanding regarding the role of NS2A⍺ during virus assembly.

2. The replication kinetics of HA-NS2A ZIKV, is shown only in the context of replicons in Figure 6. Including the replication kinetics of the constructs used for viral spread assay in fig 7 will provide a direct comparison of replication vs particle production in a full-length virus context (as only vRO positive cell number not actual replication levels were measured in figure 5). Negative strand specific qPCR can be utilized to discriminate viral replication from input RNA.

3. The TEM data in figure 5 does not have any quantification on the size of the replication organelles or their number in the mutants vs wild type. According to data in figure 5D there is only a 33% reduction in vRO positive cells in K192A and K192A mutant infected cells, which even disappears if we normalize the transfection efficiency. This data does not correlate well with the TEM data which shows a more drastic phenotype. Proper quantification of the TEM data might put all the data in proper context.

4. While the authors show that NS2A⍺ is also produced in C6/36 cells (Fig S2), no replication or particle production data is shown for insect cells. This would be of interest, to see if the functional role of NS2A⍺ in infectious virus production is extended to insect cells as well.

**********

**Part III – Minor Issues: Editorial and Data Presentation Modifications**

Please use this section for editorial suggestions as well as relatively minor modifications of existing data that would enhance clarity.

Reviewer #1: • Figure 2 would be a little easier to follow if the stars in Figure 2A had arrows and labels indicating R226 and S1D.

• For Fig 5B and Fig 5C, showing the individual data points of the independent biological replicates in the bar graphs would be helpful.

Reviewer #2: Minor comments.

1. Line: 58 and 59: little is known about form and function of NS2A – not sure that one can says this. Several NS2A studies have been described - rephrase it to tone it down a bit.

2. Fig.1 legend: add: using anti-HA antibody

3. Fig. 6: E and F – use same color code and ideally also same order..

4. Fig. S4 Legend: please explain used abbreviation CTD also in legend (that it corresponds to C terminal domain of NS2A in this case)

5. Add supplementary figure showing IF pictures (staining against NS3) after transfection of the constructs shown in Fig 5C/D.

5. Discussion: when talking about virus assembly, the insights of the following paper should be included, especially also the interaction between NS2 An and UFL1.

https://journals.asm.org/doi/10.1128/jvi.00654-25

6. Line 382 ff: NS2A remains one of the least characterized orthoflavivirus proteins, both biochemically and functionally, precluding a detailed mechanistic understanding of its contribution(s) to the viral life cycle – this seems also a little bit overstated.

7. Legend Figure 5: (C) Transfection efficiency and (D) percentage of vRO positive cells based on immunofluorescence staining for viral NS3 of pIRO-Z-transected cells.

> Shouldn't IF staining only refer to transfection efficiency (Fig. 5c)? Weren‘t the vRO postive cells determined by electron microscopy? Please rephrase accordingly.

8. Line 248: We generated a series of mutations – based on what have they been chosen? Previous publications? What was the formerly described phenotype for them?

Reviewer #3: 1. In figure 6, writing the cell line used for each sub-figure will make it clear to the reader about the conditions of the experiment, such as trans-complementation of NS2A in 6C.

2. A schematic diagram of the sub-genomic replicons and WT constructs should be included wherever used (Figure 6, 7), specially including the location of the luciferase reporter gene.

3. In fig 6, it will help the reader if the y-axis is clearly defined, rather than just %, it would be beneficial to mention % of or relative to what.

4. In figure 4A, some viruses such as JEV, MVEV, DENV, LGTV do not show production of NS2A at all, and only of NS2A⍺. Is this a representative of three biological repeats? Similarly, in fig S2-3, NS2A production in HA-NS2A ZIKV infected A549 cells is very low specially compared to C6/36 cells and NS2A-2B-3 over pression in cells. Can you speculate if that is a cell specific phenotype and if it is consistently seen with HA-NS2A ZIKV infection?

**********

PLOS authors have the option to publish the peer review history of their article (what does this mean?). If published, this will include your full peer review and any attached files.

If you choose “no”, your identity will remain anonymous but your review may still be made public.

**Do you want your identity to be public for this peer review?**  For information about this choice, including consent withdrawal, please see our Privacy Policy.

Reviewer #1: No

Reviewer #2: No

Reviewer #3: No

**Figure resubmission:**

**Reproducibility:**

?>

# Author comment: Author response to Decision Letter 1

**DOI:** 10.1371/journal.ppat.1014609.r003

14 Aug 2026

Attachment Submitted filename: Response to Reviewers.10Aug2026.docx

# Peer review: Decision Letter 1

**DOI:** 10.1371/journal.ppat.1014609.r004

**Authors:** Andrea V. Gamarnik, Sonja M. Best

8 Sep 2026

Dear Dr. Sagan,

We are pleased to inform that your manuscript, "Zika virus produces two isoforms of the NS2A protein that play mutually exclusive roles in viral replication and virion assembly.", has been editorially accepted for publication at PLOS Pathogens.

Before your manuscript can be formally accepted and sent to production, you will need to complete our formatting changes, which you will receive by email within a week. Please note that your manuscript will not be scheduled for publication until you have made the required changes.

IMPORTANT NOTES

(1) Copyediting and Proofreading: The corresponding author will receive a typeset proof for review, to ensure errors have not been introduced during production. Please review the PDF proof of your manuscript carefully, as this is the last chance to correct any errors. Please note that major changes, or those which affect the scientific understanding of the work, will likely cause delays to the publication date of your manuscript.

(2) Appropriate Figure Files: Please remove all name and figure # text from your figure files. Please also take this time to check that your figures are of high resolution, which will improve the readbility of your figures and help expedite your manuscript's publication. Please note that figures must have been originally created at 300dpi or higher. Do not manually increase the resolution of your files. For instructions on how to properly obtain high quality images, please review our Figure Guidelines, with examples at: http://journals.plos.org/plospathogens/s/figures.

We encourage you to use PACE (http://pace.apexcovantage.com/), a figure-checking tool which helps authors ensure all figures meet PLOS requirements so that figures can publish in the highest quality. PACE is a digital diagnostic and conversion tool for figure files. It will provide information about any failed check(s) and, if able, will automatically convert the figure into an acceptable file that passes quality checks.

To use PACE, you must first register as a user. Then, login and navigate to the UPLOAD tab, where you will find detailed instructions on how to use the tool.

(3) Striking Image: Please upload a striking still image to accompany your article if one is available (you can include a new image or an existing one from within your manuscript). Should your paper be accepted, this image will be considered for our monthly issue image and may also appear on our website to feature your article. Please upload this as a separate file, selecting "striking image" as the file type upon upload. Please also include a separate "Other" file with a caption, including credits and any potential copyright information. Please do not include the caption in the main article file. If your image is from someone other than yourself, please ensure that the artist has read and agreed to the terms and conditions of the Creative Commons Attribution License at http://journals.plos.org/plospathogens/s/content-license. Please note that PLOS cannot publish copyrighted images.

(4) Press Release or Related Media: If you or your institution will be preparing press materials for this manuscript, please inform our press team in advance at plospathogens@plos.org as soon as possible. We ask that you contact us within one week to plan ahead of our fast Production schedule. If you need to know your paper's publication date for related media purposes, you must coordinate with our press team, and your manuscript will remain under a strict press embargo until the publication date and time.

(5) Update your Profile Information: Now that your manuscript has been provisionally accepted, please log into Editorial Manager and update your profile, if needed. Go to http://www.editorialmanager.com/ppathogens, log in, and click on the "Update My Information" link at the top of the page. Please update your user information to ensure an efficient production and billing process.

(6) LaTeX users only: Our staff will ask you to upload a TEX file in addition to the PDF before the paper can be sent to typesetting, so please carefully review our Latex Guidelines http://journals.plos.org/plospathogens/s/latex in the meantime.

Thank you again for supporting Open Access publishing; we are looking forward to publishing your work in PLOS Pathogens.

Best regards,

Andrea V. Gamarnik

Academic Editor

PLOS Pathogens

Sonja Best

Section Editor

PLOS Pathogens

Dominique Soldati-Favre

Editor-in-Chief

PLOS Pathogens

***********************************************************

The authors have addressed the reviewers comments and questions.

Reviewer's Responses to Questions

**Part I - Summary**

Please use this section to discuss strengths/weaknesses of study, novelty/significance, general execution and scholarship.<br/><br/>

Reviewer #1: The authors have very nicely addressed all of my previous comments.

Reviewer #3: This work examines the roles of two isoforms of Zika virus NS2A protein in virus replication and particle production. The authors demonstrate that the full length NS2A is required for virus replication, while the truncated NS2Aalpha isoform affects virus assembly. This study reveals the existence distinct isoforms of NS2A and the distinct roles they play in ZIKV replication and virus assembly.

--------------------

**Part II – Major Issues: Key Experiments Required for Acceptance**

Please use this section to detail the key new experiments or modifications of existing experiments that should be absolutely required to validate study conclusions.<br/><br/>Generally, there should be no more than 3 such required experiments or major modifications for a "Major Revision" recommendation. If more than 3 experiments are necessary to validate the study conclusions, then you are encouraged to recommend "Reject".<br/><br/>

Reviewer #1: (No Response)

Reviewer #3: The authors have addressed all the major and minor points raised by this reviewer in their response to the reviewer and modified their manuscript to incorporate the suggested changes. No additional changes required.

--------------------

**Part III – Minor Issues: Editorial and Data Presentation Modifications**

Please use this section for editorial suggestions as well as relatively minor modifications of existing data that would enhance clarity.<br/><br/>

Reviewer #1: (No Response)

Reviewer #3: The authors have made all the requested changes.

--------------------

PLOS authors have the option to publish the peer review history of their article (what does this mean?). If published, this will include your full peer review and any attached files.

If you choose “no”, your identity will remain anonymous but your review may still be made public.

**Do you want your identity to be public for this peer review?**  For information about this choice, including consent withdrawal, please see our Privacy Policy.

Reviewer #1: No

Reviewer #3: No

# Editor report: Acceptance letter

**DOI:** 10.1371/journal.ppat.1014609.r005

**Authors:** Andrea V. Gamarnik, Sonja M. Best

Dear Dr. Sagan,

We are delighted to inform you that your manuscript, "Zika virus produces two isoforms of the NS2A protein that play mutually exclusive roles in viral replication and virion assembly.," has been formally accepted for publication in PLOS Pathogens.

We have now passed your article onto the PLOS Production Department who will complete the rest of the pre-publication process. All authors will receive a confirmation email upon publication.

Thank you again for supporting open-access publishing; we are looking forward to publishing your work in PLOS Pathogens.

Best regards,

Dominique Soldati-Favre

Editor-in-Chief

PLOS Pathogens
